# Migraine, chronic kidney disease and kidney function: observational and genetic analyses

**DOI:** 10.1007/s00439-023-02575-9

**Published:** 2023-06-12

**Authors:** Wenqiang Zhang, Li Zhang, Luo Yang, Chenghan Xiao, Xueyao Wu, Peijing Yan, Huijie Cui, Chao Yang, Jingwei Zhu, Xuan Wu, Mingshuang Tang, Yutong Wang, Lin Chen, Yunjie Liu, Yanqiu Zou, Ling Zhang, Chunxia Yang, Yuqin Yao, Jiayuan Li, Zhenmi Liu, Ben Zhang, Xia Jiang, International Headache Genetics Consortium, Verneri Anttila, Ville Artto, Andrea C. Belin, Anna Bjornsdottir, Gyda Bjornsdottir, Dorret I. Boomsma, Sigrid Børte, Mona A. Chalmer, Daniel I. Chasman, Bru Cormand, Ester Cuenca-Leon, George Davey-Smith, Irene de Boer, Martin Dichgans, Tonu Esko, Tobias Freilinger, Padhraig Gormley, Lyn R. Griffiths, Eija Hämäläinen, Thomas F. Hansen, Aster V. E. Harder, Heidi Hautakangas, Marjo Hiekkala, Maria G. Hrafnsdottir, M. Arfan Ikram, Marjo-Riitta Järvelin, Risto Kajanne, Mikko Kallela, Jaakko Kaprio, Mari Kaunisto, Lisette J. A. Kogelman, Espen S. Kristoffersen, Christian Kubisch, Mitja Kurki, Tobias Kurth, Lenore Launer, Terho Lehtimäki, Davor Lessel, Lannie Ligthart, Sigurdur H. Magnusson, Rainer Malik, Bertram Müller-Myhsok, Carrie Northover, Dale R. Nyholt, Jes Olesen, Aarno Palotie, Priit Palta, Linda M. Pedersen, Nancy Pedersen, Matti Pirinen, Danielle Posthuma, Patricia Pozo-Rosich, Alice Pressman, Olli Raitakari, Caroline Ran, Gudrun R. Sigurdardottir, Hreinn Stefansson, Kari Stefansson, Olafur A. Sveinsson, Gisela M. Terwindt, Thorgeir E. Thorgeirsson, Arn M. J. M. van den Maagdenberg, Cornelia van Duijn, Maija Wessman, Bendik S. Winsvold, John-Anker Zwart

**Affiliations:** 1 Department of Epidemiology and Biostatistics, Institute of Systems Epidemiology, West China-PUMC C. C. Chen Institute of Health, West China School of Public Health and West China Fourth Hospital, Sichuan University, No. 16, Section 3, South Renmin Road, Wuhou District, Chengdu, 610041 China; 2 Department of Urology, West China School of Public Health and West China Fourth Hospital, Sichuan University, Chengdu, China; 3 Department of Maternal, Child and Adolescent Health, West China School of Public Health and West China Fourth Hospital, Sichuan University, Chengdu, China; 4 Department of Iatrical Polymer Material and Artificial Apparatus, School of Polymer Science and Engineering, Sichuan University, Chengdu, China; 5 Department of Occupational and Environmental Health, West China School of Public Health and West China Fourth Hospital, Sichuan University, Chengdu, China; 6 Department of Nutrition and Food Hygiene, West China School of Public Health and West China Fourth Hospital, Sichuan University, Chengdu, China; 7 Department of Clinical Neuroscience, Karolinska Institutet, Stockholm, Sweden

## Abstract

Epidemiological studies demonstrate an association between migraine and chronic kidney disease (CKD), while the genetic basis underlying the phenotypic association has not been investigated. We aimed to help avoid unnecessary interventions in individuals with migraine through the investigation of phenotypic and genetic relationships underlying migraine, CKD, and kidney function. We first evaluated phenotypic associations using observational data from UK Biobank (*N* = 255,896). We then investigated genetic relationships leveraging genomic data in European ancestry for migraine (*N*_case_/*N*_control_ = 48,975/540,381), CKD (*N*_case_/*N*_control_ = 41,395/439,303), and two traits of kidney function (estimated glomerular filtration rate [eGFR, *N* = 567,460] and urinary albumin-to-creatinine ratio [UACR, *N* = 547,361]). Observational analyses suggested no significant association of migraine with the risk of CKD (HR = 1.13, 95% CI = 0.85–1.50). While we did not find any global genetic correlation in general, we identified four specific genomic regions showing significant for migraine with eGFR. Cross-trait meta-analysis identified one candidate causal variant (rs1047891) underlying migraine, CKD, and kidney function. Transcriptome-wide association study detected 28 shared expression–trait associations between migraine and kidney function. Mendelian randomization analysis suggested no causal effect of migraine on CKD (OR = 1.03, 95% CI = 0.98–1.09; *P* = 0.28). Despite a putative causal effect of migraine on an increased level of UACR (log-scale-beta = 0.02, 95% CI = 0.01–0.04;* P* = 1.92 × 10^−3^), it attenuated to null when accounting for both correlated and uncorrelated pleiotropy. Our work does not find evidence supporting a causal association between migraine and CKD. However, our study highlights significant biological pleiotropy between migraine and kidney function. The value of a migraine prophylactic treatment for reducing future CKD in people with migraine is likely limited.

**Supplementary Information:**

The online version contains supplementary material available at 10.1007/s00439-023-02575-9.

## Introduction

The vascular dysfunction basis of migraine, a common neurological disorder, is well established (Gormley et al. 2016; Hautakangas et al. 2022). Accumulating evidence from observational studies has demonstrated a long-term risk of macrovascular complications among individuals with migraine, including a 30% significantly increased risk of stroke and a 36% significantly increased risk of myocardial infarction compared to the general population according to results from the most updated meta-analysis (Ng et al. 2022). Such a link, however, stays weak for microvascular complications with most evidence restricted to retinal abnormalities (Al-Moujahed et al. 2021; Lin et al. 2021). Despite studies having identified an association of migraine with endothelial dysfunction (Tietjen 2009), which might lead to abnormal kidney function (Chauhan et al. 2019), only one nationwide population-based cohort study reports individuals with migraine to be at a 22% increased risk of chronic kidney disease (CKD) compared to individuals without migraine, independent of migraine medications (Weng et al. 2017).

The brain–kidney interconnection has long been identified, as both display similar anatomical and functional microvascular regulations, and are regarded as end organs on parallel trajectories. Both also share cardiometabolic risk factors, with inflammation- and oxidative stress- induced microvascular dysfunctions usually starting in low-resistance vascular beds and endothelial structures (Murray 2009; Seliger and Longstreth 2008). Current progresses from genomic and proteomic studies have highlighted common pathogenic mechanisms underlying migraine and CKD involving vascular development and endothelial function (Carlsson et al. 2017; Gormley et al. 2016; Guo et al. 2020), and loci mapping to genes *CPS1* (Choquet et al. 2021; Wuttke et al. 2019) and *SMG6* (Hautakangas et al. 2022; Wuttke et al. 2019) are found to affect both migraine and kidney function. All these results suggest the observed phenotypic association be, at least in part, attributable to shared genetic basis.

Investigating the genetic contributions to the epidemiological associations helps to elucidate intrinsic biological mechanisms underlying migraine and CKD, which may aid clinical and public health practice, for example, to help doctors cut rates of unnecessary interventions for individuals with migraine. A genome-wide cross-trait analysis is an efficient approach to understand the intrinsic relationship across complex traits (Zhu et al. 2021). Such analysis leverages summary statistics from genome-wide association studies (GWAS) and cutting-edge statistical methods, including a genetic correlation analysis to quantify global and local genetic overlap, a cross-trait meta-analysis to identify specific shared variants, a transcriptome-wide association study to detect tissue-specific shared genes, and a Mendelian randomization analysis to make causal inference. To the best of our knowledge, no such study has been performed to systematically investigate the shared etiology underlying migraine and CKD.

**Fig. 1:**
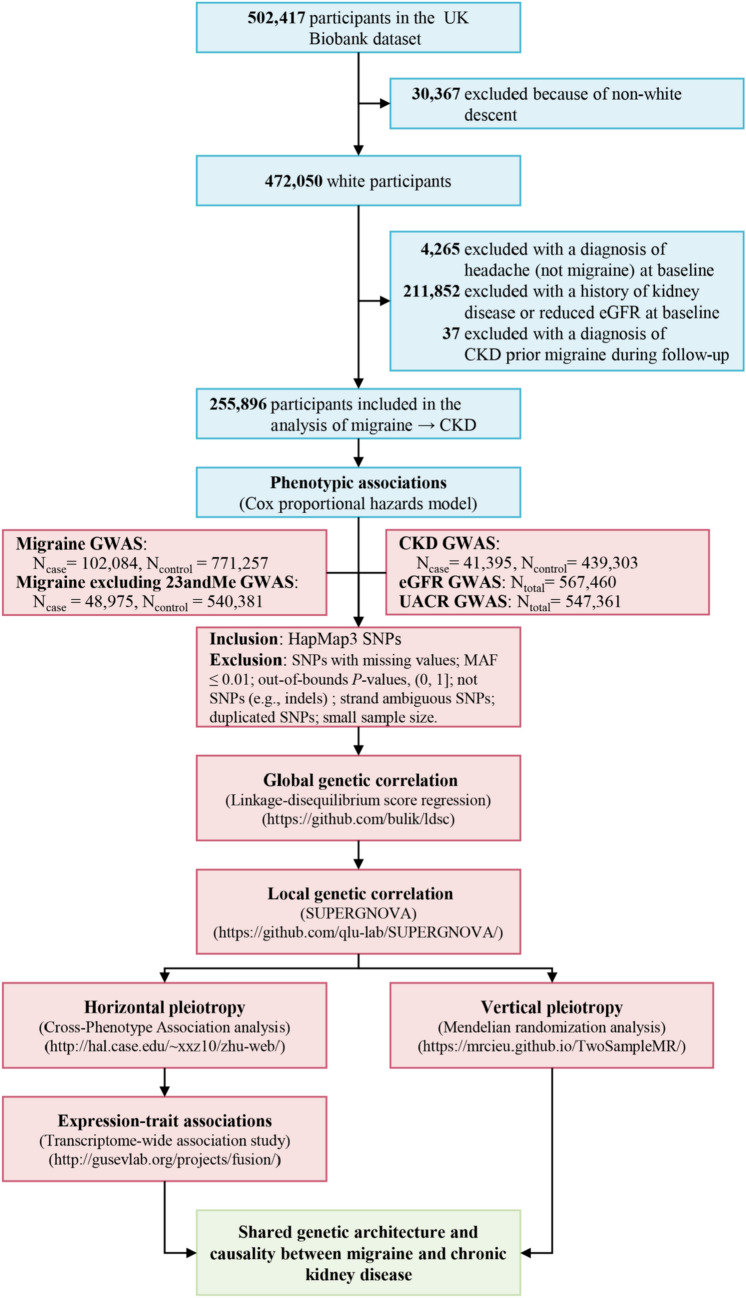
Flowchart of the overall study design in European ancestry individuals. *CKD* chronic kidney disease; *eGFR* estimated glomerular filtration rate; *UACR* urinary albumin-to-creatinine ratio

Therefore, we aim to comprehensively dissect the migraine–CKD relationship, leveraging the hitherto largest observational and genetic data. We first evaluated the phenotypic association using individual-level data from 255,896 participants of UK Biobank (UKB). We next conducted a genome-wide cross-trait analysis to characterize the shared genetic architecture and causality. In addition to the binary diagnostic outcome, we further incorporated two continuous measures on kidney function. The overarching goal of our study was to gain insight into mechanistic links underpinning migraine and CKD. The overall study design is shown in Fig. 1.

## Methods

### Data sources

#### UK Biobank data

UKB is a large population-based prospective cohort study with over 500,000 individuals aged 40–69 years at baseline, recruited in 22 study assessment centers throughout UK between 2006 and 2010 (Sudlow et al. 2015). All participants provided written informed consent, and ethical approval was granted by the National Health Service North West Multi-Centre Research Ethics Committee. We restricted the sample set to 472,050 participants of white descent (data-field 21,000). We defined migraine as self-reported medical conditions code 1265 at baseline (data-field 20,002), the International Classification of Diseases, Ninth Revision (ICD-9) code 346 (data-field 41,271) and ICD-10 code G43 (data-field 41,270), and CKD as ICD-10 code N18. We excluded participants with a diagnosis of other headaches at baseline (self-reported medical conditions code 1436; ICD-10 code G44) or a history of kidney disease at baseline (ICD-9 codes 580–589; ICD-10 codes N00-N29) or reduced estimated glomerular filtration rate (eGFR < 90 mL/min per 1.73 m^2^) at baseline or had a diagnosis of CKD before migraine during follow-up. In total, 255,896 participants were included.

#### GWAS summary statistics for migraine and CKD

The hitherto largest GWAS of migraine was conducted by the International Headache Genetics Consortium (IHGC), aggregating five study collections (IHGC, 23andMe, UK Biobank, GeneRISK, Nord-Trøndelag Health Study) totaling ~ 870,000 individuals of European ancestry (102,084 cases and 771,257 controls) (Hautakangas et al. 2022). Migraine in IHGC was ascertained based on clinical phenotyping, while migraine in other studies was verified on self-report. An inverse-variance weighted fixed-effect meta-analysis was performed to combine effect sizes for each variant across studies, adjusted for sex and at least four leading principal components of the genetic population structure. We extracted the information of 123 genome-wide significant (*P* < 5 × 10^–8^) independent SNPs and used these SNPs as IVs (Supplementary Tables 1, 2 and 3). We also applied and obtained GWAS summary statistics (48,975 cases and 540,381 controls, excluding 23andMe) for other analyses.

Given the heterogeneity of migraine subtypes, to better reflect the subtype specificity of migraine and to improve the robustness of findings, additional 4 independent SNPs associated with migraine with aura (MA) and 15 independent SNPs associated with migraine without aura (MO) were used as IVs (Hautakangas et al. 2022).

The hitherto largest GWAS of CKD was conducted by the CKD Genetics (CKDGen) Consortium, aggregating 23 participating studies totaling ~ 480,000 individuals of European ancestry (41,395 cases and 439,303 controls) (Wuttke et al. 2019). CKD was defined as an eGFR below 60 ml/min per 1.73 m^2^. An inverse-variance weighted fixed-effect meta-analysis was conducted to combine effect sizes for each variant across studies. As CKD-associated SNPs were not reported by the original GWAS, we thus identified 27 genome-wide significant independent SNPs using PLINK clumping function (parameters: clump-p1 = 5e−8, clump-p2 = 1e−5, clump-r2 = 0.1, clump-kb = 500, pop = “EUR”) (Hemani et al. 2018).

While CKD represents the disease status, to better reflect different stages of CKD, we also included two critical measures of kidney function, eGFR (Wuttke et al. 2019) and urinary albumin-to-creatinine ratio (UACR) (Teumer et al. 2019). The hitherto largest GWAS of eGFR and UACR were conducted by the CKDGen Consortium, aggregating 42 participating studies totaling 567,460 individuals for eGFR and 18 participating studies totaling 547,361 individuals for UACR. An inverse-variance weighted fixed-effect meta-analysis was conducted to combine effect sizes in log (eGFR) and log (UACR) for each variant across studies, adjusted for sex and age. A total of 256 independent eGFR-associated SNPs and 61 independent UACR-associated SNPs were identified and used as IVs. We extracted the effect size and relevant information of these IVs (Supplementary Tables 4, 5 and 6), as well as downloaded full set GWAS summary statistics for other analyses.

### Statistical analysis

#### Observational analysis

Baseline characteristics of UKB participants were presented as mean ± standard deviation (SD) for continuous variables, and as counts and percentages for categorical variables. Person-years at risk for the migraine-free category (unexposed) were accumulated from baseline until migraine diagnosis, CKD diagnosis, death, loss to follow-up, or end of follow-up, whichever came first. For the migraine category (exposed), person-years at risk were accumulated from baseline or migraine diagnosis during follow-up until CKD diagnosis, death, loss to follow-up, or end of follow-up, whichever came first. We constructed a Cox proportional hazards regression model with exposure to migraine modeled as a time-dependent variable. We used three sets of adjustments. Estimates in model 1 (basic model) were adjusted only for sex and age. Estimates in model 2 (antimigraine use model) were further adjusted for antimigraine medication usage (Anatomical Therapeutic Chemical classification code N02C; data-field 20,003), in addition to sex and age. Estimates in model 3 (full model) were further adjusted for assessment center, income, Townsend deprivation index, smoking, drinking, physical activity, sleep duration, BMI, type 2 diabetes mellitus, hypertension, and dyslipidemia on top of model 2. In the sensitivity analysis, we excluded participants with less than a year of follow-up or a diagnosis of CKD within a year after developing migraine. All statistical analyses were done using SAS version 9.4 (SAS Institute, Cary, NC). A two-sided *P* value of less than 0.05 was considered statistically significant.

#### Genome-wide genetic correlation analysis

Genetic correlation represents an average sharing of genetic effect between two traits that is independent of environmental confounders. We first quantified the global genetic correlation (*r*_*g*_) across the genome using software linkage-disequilibrium score regression (LDSC) (Bulik-Sullivan et al. 2015). The method uses only GWAS summary statistics, relying on the fact that the effect size estimate for a given SNP aggregates the effects of all SNPs in LD with that SNP. Genetic correlation ranges between − 1 and + 1. We used pre-calculated HapMap3 LD scores computed from ~ 1.2 million common SNPs in European ancestry, commonly acknowledged as well imputed. A Bonferroni-corrected P-threshold (*P* < 0.017 = 0.05/3) was used to define statistical significance.

While global genetic correlation depicts an average of shared signal across the whole genome, it may fail to identify scenarios where the signal is restricted to particular genomic regions or in opposing directions at different loci. We thus estimated the pairwise local genetic correlation using software SUPERGNOVA (Zhang et al. 2021). This algorithm partitions the whole genome into 2353 approximate LD-independent regions with an average length of 1.6 centimorgans and quantifies genetic correlation confined to these genomic regions. A Bonferroni-corrected P-threshold (*P* < 2.12 × 10^–5^ = 0.05/2,353) was used to define statistical significance.

#### Cross-trait meta-analysis

Genetic correlation reflects either horizontal pleiotropy (pleiotropy) or vertical pleiotropy (causality). In horizontal pleiotropy, a genetic variant has independent effects on multiple traits, whereas in vertical pleiotropy, a genetic variant has an effect on a trait through its genetic effect on an intermediate trait. We next conducted a cross-trait meta-analysis to identify pleiotropic variants that simultaneously influence both traits using software Cross-Phenotype Association (CPASSOC) (Zhu et al. 2015). CPASSOC combines GWAS summary statistics to test the association of each SNP with at least two traits, controlling for population structure or cryptic relatedness. We calculated pairwise S_Het_ based on a fixed-effect model, a test statistic that is more powerful when heterogeneity (including opposite directional allelic effects) exists.

After CPASSOC, we obtained independent shared variants via PLINK clumping (parameters: –clump-p1 5e−8 –clump-p2 1e−5 –clump-r2 0.2 –clump-kb 500). Significant index SNP was defined as *P*_CPASSOC_ < 5 × 10^–8^ and *P*_migraine_ _or_ _CKD_ _or_ _kidney function_ < 1 × 10^–5^. Novel index SNP was defined only if all following three criteria were met: (1) the SNP reached genome-wide significance (*P*_CPASSOC_ < 5 × 10^–8^) in CPASSOC; (2) the SNP did not reach genome-wide significance (5 × 10^–8^ < *P*_migraine or CKD or kidney function_ < 1 × 10^–5^) in original single-trait GWAS; (3) the SNP was not in LD (${r}^{2}$ < 0.05) with any of those previously reported genome-wide significant SNPs from both single-trait GWAS.

Ensembl Variant Effect Predictor (VEP, https://grch37.ensembl.org/info/docs/tools/vep/index.html) was used to map the shared SNPs identified by CPASSOC to its nearest gene.

#### Fine-mapping credible set analysis and colocalization analysis

An index SNP does not represent a causal SNP. We further identified a credible set of variants with a 99% probability of containing the causal variant at each of the identified pleiotropic loci using FM-summary (Huang et al. 2017). Specifically, we extracted variants within 500 kb of the index SNP at each locus and estimated posterior inclusion probability (PIP, the probability of including a SNP as causal) for each variant by setting a flat prior with the steepest descent approximation. A 99% credible set corresponds to ranking SNPs from largest to smallest PIPs and taking the cumulative sum of PIPs until it is at least 99%.

We also conducted Coloc (Giambartolomei et al. 2014) to examine whether cross-trait meta-analysis identified shared loci colocalized at the same causal variant. Coloc is a Bayesian algorithm calculating the posterior probabilities of different causal variant configurations under the hypothesis of a single causal variant at each locus for each trait, i.e., H0 (no causal variant), H1 (causal variant for trait 1 only), H2 (causal variant for trait 2 only), H3 (two different causal variants), and H4 (a common causal variant). A shared locus was considered colocalized if the posterior probability for H4 (PPH4) was greater than 0.7.

#### Transcriptome-wide association study analysis

Cross-trait meta-analysis identifies pleiotropic variants without considering gene expression and tissue specificity while many genetic variants lead to complex traits via regulating tissue-specific gene expressions. We performed a transcriptome-wide association study (TWAS) analysis using FUSION (Gusev et al. 2016) to identify regulated genes whose expression pattern across tissues implicates shared biological mechanisms. We first conducted single-trait TWAS using the expression weights from 48 post-mortem Genotype–Tissue Expression project (GTEx) tissues and CommonMind Consortium (CMC) brain tissue. The Bonferroni correction (*P*_Bonferroni_ < 0.05) within each tissue was used to identify significant expression–trait associations. We then performed joint/conditional tests for loci with multiple associated features to determine independent genes at each locus. Colocalization analysis was further conducted to examine whether GWAS signals and GTEx expression quantitative trait loci (eQTL) signals were colocalized at the same causal variant. We then integrated these results across traits to identify shared gene–tissue pairs.

#### Mendelian randomization analysis

We finally performed a bidirectional two-sample Mendelian randomization (MR) analysis to assess putative causal relationships via software TwoSampleMR (Hemani et al. 2018). We applied the inverse-variance weighted (IVW) method (Burgess et al. 2015) as our primary analytical method assuming all IVs to be valid, which provided the greatest statistical power. We next performed sensitivity analyses using MR-Egger regression (Bowden et al. 2015) and weighted-median method (Bowden et al. 2016) to examine the robustness of primary results. We also repeated IVW excluding palindromic IVs (i.e., A/T or G/C alleles) or pleiotropic IVs (SNPs associated with potential confounding traits according to NHGRI-EBI GWAS Catalog, https://www.ebi.ac.uk/gwas/). We further conducted CAUSE (Causal Analysis Using Summary Effect estimates) as a complementary analysis to detect causal relationships while accounting for both correlated and uncorrelated pleiotropic effects (Morrison et al. 2020). We then performed multivariable MR to account for the effect of blood pressure (Evangelou et al. 2018), as a shared causal factor for migraine and CKD (Guo et al. 2020; Zheng et al. 2022).

We computed the phenotypic variance explained by IVs (${r}^{2}$) (Shim et al. 2015) and calculated *F*-statistics (Pierce et al. 2011) to assess the strength of IVs (Supplementary Table 7). We also computed the statistical power of MR (https://shiny.cnsgenomics.com/mRnd/).

## Results

### Phenotypic association

The baseline characteristics of UKB participants included in the observational analysis are presented in Supplementary Table 8. Participants were followed for 3,122,499 person-years (12.1 ± 2.0 years), during which 97 migraine patients and 2,461 migraine-free individuals developed CKD (Table 1). After adjusting for sex and age, migraine patients showed a significantly increased hazard of CKD (HR = 1.32, 95% CI = 1.07–1.61). With further adjustment of antimigraine medication usage, the effect attenuated to some extent (10.33%), but remained statistically significant (HR = 1.28, 95% CI = 1.02–1.60). In fully adjusted model, the effect attenuated to null (HR = 1.13, 95% CI = 0.85–1.50). No significant association (HR = 1.05, 95% CI = 0.78–1.40) was observed in sensitivity analysis.

**Table 1:** Observational associations between migraine and the risk of subsequent chronic kidney disease

Exposure status during follow-up	Cases/person-years	Primary analysis			Sensitivity analysis
		Basic model	Basic model + antimigraine use	Full model	Full model
Migraine					
No	2461/3,013,908	1.00 (ref)	1.00 (ref)	1.00 (ref)	1.00 (ref)
Yes	97/108,591	1.32 (1.07–1.61)	1.28 (1.02–1.60)	1.13 (0.85–1.50)	1.05 (0.78–1.40)

Basic model: adjusted for sex and age

Full model: adjusted for sex, age, antimigraine medication usage, assessment center, income, Townsend deprivation index, smoking, drinking, physical activity (IPAQ), sleep duration, BMI, type 2 diabetes mellitus, hypertension, and dyslipidemia

*IPAQ* International Physical Activity Questionnaire; *BMI* body mass index

### Global and local genetic correlation

**Fig. 2:**
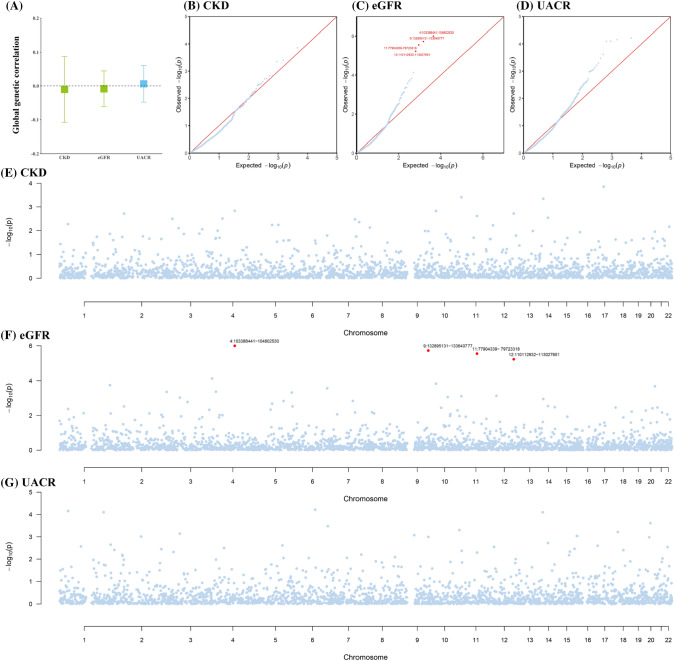
Genome-wide genetic correlation between migraine and chronic kidney disease. The boxes (**A**) denote point estimates of the global genetic correlation, and the error bars denote 95% confidence intervals (CI). The blue color indicates a positive genetic corralation and the green color indicates a negative genetic corralation. In the QQ plots (**B–D**) and Manhattan plots (**E–G**), each point presents a specific genomic region, while red points represent genomic regions that contribute significant local genetic correlation as estimated by SUPERGNOVA (*P* < 0.05/2353). CKD, chronic kidney disease; eGFR, estimated glomerular filtration rate; UACR, urinary albumin-to-creatinine ratio

As shown in Fig. 2A, no significant global genetic correlation between migraine and CKD (${r}_{g}$ = − 0.01, *P* = 0.84) was found. For kidney function, no significant global genetic correlation was observed for migraine with either eGFR (${r}_{g}$ = − 0.01, *P* = 0.75) or UACR (${r}_{g}$ = 0.01, *P* = 0.82).

Partitioning the whole genome into 2353 LD-independent regions and after correcting for multiple testing (*P* < 2.12 × 10^–5^), no significant local signal was identified between migraine and CKD (Fig. 2B–G and Supplementary Table 9). For kidney function, significant local signal was observed for migraine with eGFR at four genomic regions (4q24, 9q34.1, 11q14.1, 12q24.1). Of note, 4q24 (chromosome 4: 103,388,441–104,802,530), with the strongest local effect, harbors gene *NFKB1* encoding the subunits of nuclear factor‑κB (NF‑κB) transcription factor, known to associate with migraine (Reuter et al. 2002), kidney function, and CKD (O'Brown et al. 2015).

### Cross-trait meta-analysis

Given the evidence of significant local genetic overlap, we further performed pairwise CPASSOC to identify pleiotropic loci (Table 2 and Supplementary Table 10). In total, 11 independent pleiotropic SNPs reached genome-wide significance (*P*_CPASSOC_ < 5 × 10^–8^) in cross-traits and suggestive significance (*P*_migraine/CKD/kidney function_ < 1 × 10^–5^) in single traits, including one locus shared between migraine and CKD (rs1047891), seven loci shared between migraine and eGFR (rs1566225, rs41272663, rs1047891, rs13099628, rs6776700, rs62576116, rs9894634), and three loci shared between migraine and UACR (rs1047891, rs1971819, rs4909945). Of note, rs1047891 was shared across all traits. This SNP is located near *CPS1*, previously reported to associate with migraine in women (Choquet et al. 2021) and eGFR (Kottgen et al. 2010).

**Table 2:** Pleiotropic SNPs identified by cross-trait meta-analysis between migraine and chronic kidney disease

SNP	Novel	A1	A2	Beta		P-migraine	P-kidney	P-CPASSOC	Mapped genes
				Migraine	CKD				
Migraine and CKD									
rs1047891	No	A	C	0.041	0.055	8.22 × 10^−07^	2.28 × 10^−07^	6.13 × 10^−12^	*CPS1*
Migraine and eGFR									
rs1566225	No	G	C	0.035	− 0.002	7.90 × 10^−06^	2.81 × 10^−12^	2.61 × 10^−15^	*RPRD2*
rs41272663	Yes	A	C	− 0.043	0.002	1.34 × 10^−06^	1.08 × 10^−07^	2.91 × 10^−12^	*LANCL1, AC007970.1*
rs1047891	No	A	C	0.041	− 0.007	8.22 × 10^−07^	3.59 × 10^−64^	9.35 × 10^−65^	*CPS1*
rs13099628	Yes	G	T	0.040	− 0.002	4.86 × 10^−06^	4.82 × 10^−06^	4.18 × 10^−10^	*SCN11A*
rs6776700	No	A	G	0.036	0.002	2.50 × 10^−06^	1.83 × 10^−11^	2.95 × 10^−15^	*ATRIP*
rs62576116	No	A	G	0.055	0.004	3.54 × 10^−07^	4.28 × 10^−12^	8.05 × 10^−17^	*ASTN2, RP11-67K19.3*
rs9894634	No	C	T	0.034	0.002	9.13 × 10^−06^	1.69 × 10^−09^	6.16 × 10^−13^	*SMG6, HIC1*
Migraine and UACR									
rs1971819	No	G	C	− 0.054	− 0.019	6.62 × 10^−08^	4.66 × 10^−14^	1.03 × 10^−19^	*ICA1L, KRT8P15*
rs1047891	No	A	C	0.041	− 0.019	8.22 × 10^−07^	2.55 × 10^−18^	1.06 × 10^−21^	*CPS1*
rs4909945	No	T	C	− 0.067	− 0.010	5.08 × 10^−16^	6.46 × 10^−06^	2.28 × 10^−19^	*MRVI1*

Novel: novel SNPs only if all following criteria were satisfied: (1) the SNP reached genome-wide significance (*P*_CPASSOC_ < 5 × 10^−8^) in CPASSOC; (2) the SNP did not reach genome-wide significance (5 × 10^−8^ < *P*_GWAS_ < 10^−5^) in both single-trait GWAS(s); (3) the SNP was not in LD (*r*^2^ < 0.05) with any of those previously reported genome-wide significant SNPs of single traits

*CKD* chronic kidney disease; *eGFR* estimated glomerular filtration rate; *UACR* urinary albumin-to-creatinine ratio

After excluding SNPs that reached genome-wide significance in single traits (*P*_migraine/CKD/kidney function_ < 5 × 10^–8^) or were in LD (${r}^{2}$ ≥ 0.05) with any of the previously reported genome-wide significant SNPs (Supplementary Table 11), two novel SNPs (rs41272663, rs13099628) were identified to be shared between migraine and eGFR. SNP rs41272663 was mapped to *LANCL1*, an antioxidant gene protecting neurons from oxidant damage (Huang et al. 2014). SNP rs13099628 was mapped to *SCN11A*, encoding a voltage-gated sodium ion channel (Na_v_1.9) associated with pain perception (Leipold et al. 2013). Detailed annotations for all 11 shared loci are shown in Supplementary Table 12.

For each of the 11 shared loci, a 99% credible set of the causal variant (Supplementary Table 13) was determined, including a median of 22 variants (ranges: 1–197). Notably, at the locus of index SNP rs1047891, the 99% credible set consisted of only one single variant (rs1047891 itself) for migraine–eGFR and migraine–UACR, and of only two variants (rs1047891 and rs715) for migraine–CKD, stressing again its putative causal role.

Furthermore, for the 11 shared loci, a majority (seven, 64%) colocalized at the same candidate causal variant (PPH4 > 0.7), while four colocalized at different candidate causal variants (PPH3 > 0.7). Of note, both fine-mapping and colocalization analyses supported a shared causal variant for migraine with CKD, eGFR, and UACR at rs1047891 (Supplementary Table 14).

### Transcriptome-wide association study

To investigate specific tissue–gene pairs shared by migraine, CKD, and kidney function, we performed TWAS using two different data sources of gene expression (GTEx and CMC). No overlapping tissue–gene pair was found for migraine and CKD using either tissue. For kidney function, 19 significant tissue–gene pairs were found for migraine and eGFR using GTEx tissues, including four genes (***TREX1***, *SHISA5*, *PRR13*, *TMA7*) mainly expressed in tissues of the nervous and cardiovascular system (Table 3). Among these genes, ***TREX1*** remained significant using CMC brain tissues, previously reported to associate with migraine (Sutherland and Griffiths 2017). Furthermore, nine significant tissue–gene pairs were found for migraine and UACR using GTEx tissues, including four genes (*NBEAL1*, *FAM117B*, ***ICA1L***) mainly expressed in tissues of the nervous and cardiovascular system. Among these genes, ***ICA1L*** remained significant using CMC brain tissues, which was also identified by our cross-trait meta-analysis and the previous UACR GWAS (Teumer et al. 2019).

**Table 3:** Shared TWAS significant genes between migraine and chronic kidney disease across 48 GTEx tissues (version 7) and CMC brain tissue

Tissue	Gene	CHR	N_SNP_	Migraine			CKD related phenotypes		
				BEST.GWAS.ID	TWAS.P	PPH4	BEST.GWAS.ID	TWAS.P	PPH4
Migraine and eGFR									
GTEx adipose subcutaneous	*TREX1*	3	257	rs6776700	3.91 × 10^−06^	0.95	rs6776700	2.28 × 10^−10^	0.89
GTEx adrenal gland	*TREX1*	3	257	rs6776700	3.73 × 10^−06^	0.95	rs6776700	3.61 × 10^−11^	0.98
GTEx artery aorta	*TREX1*	3	257	rs6776700	3.91 × 10^−06^	0.95	rs6776700	2.28 × 10^−10^	0.89
GTEx artery coronary	*PRR13*	12	408	rs3816806	2.58 × 10^−06^	0.98	rs10876470	1.48 × 10^−05^	0.84
	*TREX1*	3	257	rs6776700	3.91 × 10^−06^	0.94	rs6776700	2.28 × 10^−10^	0.88
GTEx artery tibial	*TREX1*	3	257	rs6776700	4.20 × 10^−06^	0.94	rs6776700	3.36 × 10^−11^	0.98
GTEx brain anterior cingulate cortex BA24	*TMA7*	3	258	rs6776700	2.46 × 10^−06^	0.95	rs6776700	1.21 × 10^−10^	0.97
GTEx brain cerebellar hemisphere	*TMA7*	3	258	rs6776700	3.42 × 10^−06^	0.95	rs6776700	9.58 × 10^−11^	0.97
GTEx brain cortex	*TMA7*	3	257	rs6776700	2.46 × 10^−06^	0.96	rs6776700	1.86 × 10^−11^	0.99
GTEx brain frontal cortex BA9	*TMA7*	3	258	rs6776700	2.76 × 10^−06^	0.95	rs6776700	3.37 × 10^−11^	0.98
GTEx cells transformed fibroblasts	*TREX1*	3	257	rs6776700	3.91 × 10^−06^	0.95	rs6776700	2.28 × 10^−10^	0.89
GTEx colon sigmoid	*TREX1*	3	257	rs6776700	3.00 × 10^−06^	0.95	rs6776700	4.94 × 10^−10^	0.77
GTEx esophagus gastroesophageal junction	*TREX1*	3	257	rs6776700	2.46 × 10^−06^	0.96	rs6776700	1.86 × 10^−11^	0.99
GTEx esophagus mucosa	*TREX1*	3	257	rs6776700	3.72 × 10^−07^	0.94	rs6776700	4.26 × 10^−11^	0.90
GTEx heart atrial appendage	*TMA7*	3	258	rs6776700	1.64 × 10^−06^	0.95	rs6776700	1.20 × 10^−10^	0.98
GTEx heart left ventricle	*TMA7*	3	258	rs6776700	2.85 × 10^−06^	0.96	rs6776700	3.80 × 10^−11^	0.98
GTEx muscle skeletal	*TMA7*	3	258	rs6776700	2.85 × 10^−06^	0.96	rs6776700	3.80 × 10^−11^	0.98
GTEx skin not sun exposed suprapubic	*SHISA5*	3	254	rs6776700	2.29 × 10^−06^	0.95	rs6776700	8.97 × 10^−11^	0.91
GTEx spleen	*TREX1*	3	256	rs6776700	6.26 × 10^−06^	0.94	rs6776700	1.38 × 10^–10^	0.98
CMC brain	*TREX1*	3	257	rs6776700	2.48 × 10^−06^	0.94	rs6776700	5.49 × 10^−12^	0.97
Migraine and UACR									
GTEx adipose subcutaneous	*NBEAL1*	2	262	rs934287	2.19 × 10^−06^	0.95	rs934287	1.54 × 10^−12^	0.97
GTEx adipose visceral omentum	*NBEAL1*	2	262	rs934287	3.68 × 10^−06^	0.94	rs934287	1.06 × 10^−12^	0.98
GTEx artery aorta	*FAM117B*	2	241	rs934287	3.68 × 10^−06^	0.93	rs934287	1.06 × 10^−12^	0.97
GTEx artery coronary	*NBEAL1*	2	262	rs934287	2.73 × 10^−06^	0.96	rs934287	1.67 × 10^−12^	0.98
GTEx artery tibial	*ICA1L*	2	244	rs934287	2.62 × 10^−06^	0.79	rs934287	1.93 × 10^−12^	0.80
GTEx nerve tibial	*ICA1L*	2	244	rs934287	2.62 × 10^−06^	0.96	rs934287	1.16 × 10^−12^	0.98
GTEx pancreas	*ICA1L*	2	244	rs934287	3.42 × 10^−06^	0.95	rs934287	1.30 × 10^−12^	0.97
GTEx skin not sun exposed suprapubic	*ICA1L*	2	244	rs934287	2.73 × 10^−06^	0.96	rs934287	1.67 × 10^−12^	0.97
GTEx skin sun exposed lower leg	*NBEAL1*	2	262	rs934287	3.74 × 10^−06^	0.93	rs934287	1.10 × 10^−12^	0.97
CMC brain	*ICA1L*	2	267	rs934287	2.73 × 10^−06^	0.96	rs934287	1.67 × 10^−12^	0.97

*GTEx* Genotype–Tissue Expression Project, *CMC* CommonMind Consortium, *TWAS* transcriptome-wide association studies, *CKD*, chronic kidney disease, *eGFR* estimated glomerular filtration rate, *UACR* urinary albumin-to-creatinine ratio

### Mendelian randomization analysis

**Fig. 3:**
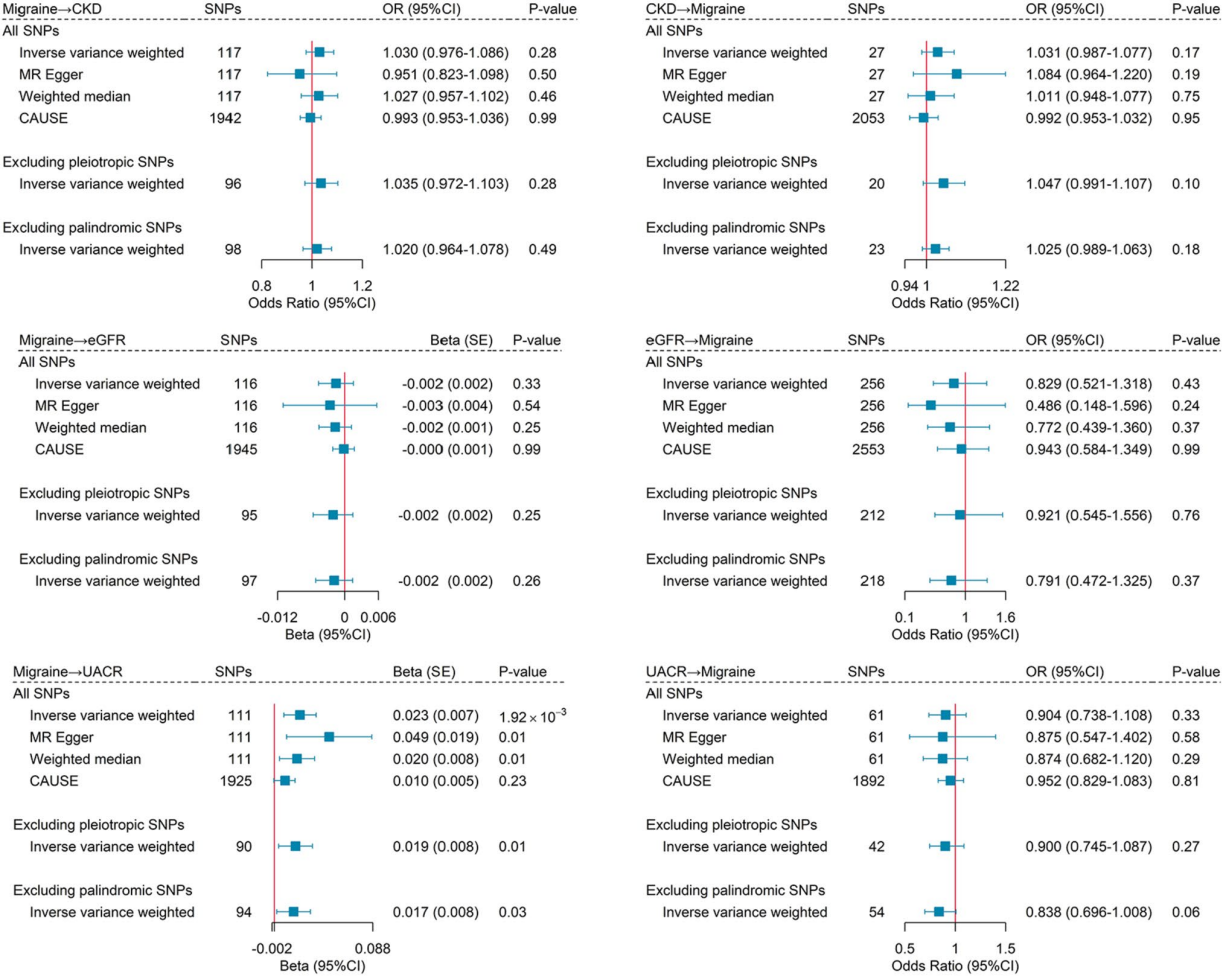
Bidirectional mendelian randomization analysis between migraine and chronic kidney disease. The blue boxes denote point estimates of the causal effects, and the error bars denote 95% confidence intervals (CI). *CKD* chronic kidney disease; *eGFR* estimated glomerular filtration rate; *UACR* urinary albumin-to-creatinine ratio

Finally, we performed a bidirectional two-sample MR to make causal inference (Fig. 3). Genetically predisposed migraine did not seem to affect CKD risk (OR = 1.03, 95% CI = 0.98–1.09; *P* = 0.28). Conversely, genetically predisposed CKD also did not seem to influence migraine onset (OR = 1.03, 95% CI = 0.99–1.08; *P* = 0.17). For kidney function, genetically predisposed migraine was significantly associated with a higher level of UACR (beta = 0.02, 95% CI = 0.01–0.04;* P* = 1.92 × 10^–3^), while the effect attenuated to null when accounting for correlated and uncorrelated pleiotropy (beta = 0.01, 95% CI = − 0.001–0.02; *P* = 0.23) or adjusting for systolic blood pressure (beta = 0.01, 95% CI = − 0.01–0.02; *P* = 0.29; Supplementary Table 15). Conversely, genetically predicted UACR was not associated with migraine (OR = 0.90, 95% CI = 0.74–1.11; *P* = 0.33). There was no significant association between migraine and eGFR. The results remained consistent when restricting analyses for MA and MO (Supplementary Fig. 1).

The mean F-statistics of our IVs were larger than 50 (Supplementary Table 7), indicating strong instruments. With the current sample size of outcome, assuming 0.81% (migraine) and 0.32% (CKD) of phenotypic variance explained by IVs based on the data we used, our study had more than 80% statistical power to detect an OR of 1.16 for migraine on CKD and 1.24 for CKD on migraine, respectively.

## Discussion

To the best of our knowledge, this is the most comprehensive observational and genetic analysis that systematically investigates the phenotypic association, genetic correlation, pleiotropic loci, expression–trait association, and causal relationship between migraine, CKD, and kidney function. In observational analysis, we found no strong phenotypic link between migraine and CKD. Genetic analysis suggested a weak genetic relationship between migraine and CKD with merely one candidate pleiotropic variant identified, while four specific genomic regions showing significant local signals were found for migraine and eGFR, relaxing the binary disease status to the continuous physiological measure. Such genetic overlap was further substantiated by 10 pleiotropic loci and 28 shared expression–trait associations for migraine and kidney function, primarily tagging tissues of nervous and cardiovascular systems. However, we found limited evidence to support a causal effect of migraine on CKD and kidney function. These findings advance our understanding of the relationship underlying migraine and CKD, providing potential implications for disease prevention strategies.

In contrast to previous observational studies, we found a minimal phenotypic and genetic link underlying migraine and CKD. In observational analysis, the age and sex-adjusted hazard of CKD was 1.32 (95% CI = 1.07–1.61), which was largely consistent with one (and the only) existing observational study reporting a relatively small effect (HR = 1.22, 95% CI = 1.02–1.47) (Weng et al. 2017). When we further adjusted for other covariates in the subset of participants with more complete data on risk factors, the effect attenuated with adjustment for antimigraine medication usage and was null in fully adjusted model. Such a weak phenotypic link was corroborated by genetic findings of neither significant a genetic correlation nor a putative causal association, with merely one pleiotropic causal variant (rs1047891) identified. These findings are perhaps not very surprising as similar patterns have been observed for migraine with macrovascular diseases, while observational studies consistently indicate strong phenotypic associations between migraine, coronary artery disease, and stroke (Ng et al. 2022), genetic analyses show minimal shared basis (Pickrell et al. 2016; Siewert et al. 2020) with significant results only found at the individual variant level.

Despite the ambiguous findings for migraine with CKD, we found consistent and robust evidence supporting for a shared genetic architecture between migraine and kidney function. This is not against our expectation as the latter reflects a continuous physiological measure, a measurement of higher granularity that could be used to determine a binary disease outcome. We identified four significant local signals for migraine and eGFR, supporting a brain–kidney interconnection driven by a shared genetic basis. The strongest local signal was at 4q24 harboring *NFKB1*, a gene encoding the subunits of NFκB transcription factor, activation of which contributes to local inflammation and headaches (Reuter et al. 2002), as well as to age-related decline in kidney function (O'Brown et al. 2015).

Results from cross-trait meta-analysis suggest that the intrinsic link observed for migraine and kidney function may largely be explained by biological pleiotropy. We highlight a candidate causal variant (rs1047891) shared for migraine with kidney function as well as with CKD. This pleiotropic SNP is located near ***CPS1***, encoding a mitochondrial enzyme named carbamoyl-phosphate synthase 1 that controls the synthesis of carbamoyl phosphate from ammonia in the initial step of urea cycle. Individuals with a ***CPS1*** deficiency typically present hyperammonemia with a wide range of clinical manifestations, including migraine, abdominal pain, vomiting, and decreased urea production (Haberle et al. 2019; Raina et al. 2020). Follow-up experimental studies are needed to validate the role of the identified candidate causal variant. Of note, we found several shared genetic variants whose signs of effect estimates are opposite to the direction that would be predicted from the global genetic correlation. This likely implies the heterogeneous pathways shared by migraine and kidney function.

TWAS analysis takes us one level down from the variant-based association to the gene-based association in disease-related or potentially pathological tissues. We found multiple shared expression–trait associations between migraine and kidney function. Here, we highlight two pleiotropic genes (***TREX1*** and ***ICA1L***) replicated by using the CMC brain tissue. ***TREX1***, shared by migraine and eGFR, and expressed in the brain, artery, spleen, and many other tissues, encodes a 3’ to 5’ DNA exonuclease known to regulate immunity and repair DNA. In human neural cells, deficiency of ***TREX1*** contributes to the accumulation of extranuclear DNA, thereby inducing neurotoxicity through increased type I interferon secretion (Thomas et al. 2017), which may play a role in the etiology of migraine (Yazgan and Naziroglu 2021). Furthermore, mutations in ***TREX1*** may contribute to renal thrombotic microangiopathy through increased type I interferon secretion and concurrent autoimmune activation (Gulati et al. 2018). In addition, ***ICA1L***, shared by migraine and UACR, and expressed in the brain, artery, pancreas, and skin tissues, encodes islet cell autoantigen 1-like (ICA1L) protein that is involved in protein domain specific binding activity and regulation of transport. Findings from cell-type specificity analysis in the brain have identified enrichment in the ICA1L expression in glutamatergic excitatory neurons (Ou et al. 2021), while malfunctioning of the glutamatergic system may cause symptoms of migraine (Gasparini et al. 2016). Previous proteome-wide association studies have discovered the brain protein abundance of ***ICA1L*** to affect cerebral small vessel diseases (Cullell et al. 2022) and Alzheimer’s disease (Ou et al. 2021). To date, little is known regarding the exact biological functions of ***TREX1*** and ***ICA1L*** in migraine and kidney function, and future functional studies are worth revealing their roles.

Our findings provide potential public health as well as clinical implications. First, migraine does not seem to cause CKD. The excess risk of CKD among individuals with migraine is probably attributed to shared environmental factors. For people with migraine to be identified as high CKD risk, the coexistence of shared risk factors is required and such established risk factors (e.g., hypertension) should be treated as clinical guideline recommendations. Avoiding unnecessary intervention makes sense for individuals with migraine because triptans as a first-line treatment for moderate or severe migraine attacks have the potential kidney toxicity to do harm (Mobasheran et al. 2020). Second, migraine, CKD, and kidney function are inherently linked through biological pleiotropy. SNP rs1047891 is a candidate causal variant shared by migraine, CKD, and kidney function. Our findings may provide implications for the design of future functional experiments. Prospectively, the identification of specific pleiotropic loci modulating common biological pathways may facilitate the discovery of broad-spectrum therapeutic targets that would benefit both the precision prevention and treatment of chronic comorbidities.

We acknowledge several potential limitations. First, our findings were restricted to individuals of European ancestry, which may not be generalizable to other ancestral populations. Further research of this topic leveraging data from other ethnicities is warranted. Second, a substantial sample overlap (59.2%) exists across GWAS of migraine and GWAS of UACR. However, the lower end of the one-sided 95% CI for F-statistic remained high (55.6 for migraine IVs and 48.1 for UACR IVs), thus considerable weak instrument bias in our two-sample MR was not expected (Burgess et al. 2016). Sample overlap is another issue that might introduce bias due to an inflated type 1 error rate (false positive findings). This, however, was not a concern in our two-sample MR analysis as all findings were null (negative findings). Future research with independent samples to derive IV-exposure and IV-outcome associations is needed. Nevertheless, the genome-wide cross-trait analytical approaches we have applied (e.g., LDSC, SUPERGNOVA, CPASSOC) are all robust to sample overlap (Bulik-Sullivan et al. 2015; Zhang et al. 2021; Zhu et al. 2015). Third, our cross-phenotype results were restricted to overall migraine without subtyping, as the sample size of the hitherto available genetic studies on migraine subtypes was too small to withstand the statistical burdens of genome-wide cross-trait analysis. Future GWAS of migraine subtypes with larger sample sizes are required to understand the subtype-specific effect. Fourth, notwithstanding substantial efforts to enhance the total sample size, we still had limited power to detect the causal effect of migraine on CKD through traditional MR, perhaps due to a small phenotypic variance explained by IVs. However, we replicated the results with an increased power by relaxing the outcome from a binary disease status (CKD) to a continuous physiological measure (eGFR and UACR), and obtained consistent findings.

## Conclusions

To conclude, leveraging large-scale observational and genetic data of European ancestry, our work does not find evidence to support a causal association between migraine and CKD. However, our study highlights significant biological pleiotropy between migraine and kidney function. Our findings provide novel insights into precision prevention and medicine for cardiovascular comorbidities with migraine.

## Supplementary Information

Below is the link to the electronic supplementary material.

Supplementary file1 (PDF 2309 KB)

## Data Availability

Data from UK Biobank is available for open access to scientific researchers (www.biobank.ac.uk). The UK Biobank analysis was conducted within the application 50,538. GWAS summary statistics for migraine excluding 23 and Me are available for bona fide researchers (contact Dale R Nyholt, d.nyholt@qut.edu.au), and GWAS summary statistics for CKD, eGFR, and UACR are available through the CKDGen Consortium (https://ckdgen.imbi.uni-freiburg.de/).

## Abbreviations

BMI: Body mass index
CKD: Chronic kidney disease
CMC: CommonMind Consortium
CPASSOC: Cross-Phenotype Association
eGFR: Estimated glomerular filtration rate
GTEx: Genotype-Tissue Expression Project
GWAS: Genome-wide association study
IHGC: International Headache Genetics Consortium
IV: Instrumental variable
IVW: Inverse-variance weighted
MA: Migraine with aura
MO: Migraine without aura
MR: Mendelian randomization
PIP: Posterior inclusion probability
TWAS: Transcriptome-wide association study
UACR: Urinary albumin-to-creatinine ratio
UKB: UK Biobank
VEP: Variant effect predictor

## References

[CR1] Al-Moujahed A, Tran EM, Azad A, Vail D, Ludwig CA, Pasricha MV, Rosenblatt TR, Callaway NF, Moshfeghi DM (2021). Risk of Retinal Artery Occlusion in Patients with Migraine. Am J Ophthalmol.

[CR2] Bowden J, Davey Smith G, Burgess S (2015). Mendelian randomization with invalid instruments: effect estimation and bias detection through Egger regression. Int J Epidemiol.

[CR3] Bowden J, Davey Smith G, Haycock PC, Burgess S (2016). Consistent estimation in mendelian randomization with some invalid instruments using a weighted median estimator. Genet Epidemiol.

[CR4] Bulik-Sullivan B, Finucane HK, Anttila V, Gusev A, Day FR, Loh PR, ReproGen C, Psychiatric Genomics C, Duncan L, Perry JR, Patterson N, Robinson EB, Daly MJ, Price AL, Neale BM, Genetic Consortium for Anorexia Nervosa of the Wellcome Trust Case Control C (2015). An atlas of genetic correlations across human diseases and traits. Nat Genet.

[CR5] Burgess S, Scott RA, Timpson NJ, Davey Smith G, Thompson SG, Consortium E-I (2015). Using published data in Mendelian randomization: a blueprint for efficient identification of causal risk factors. Eur J Epidemiol.

[CR6] Burgess S, Davies NM, Thompson SG (2016). Bias due to participant overlap in two-sample Mendelian randomization. Genet Epidemiol.

[CR7] Carlsson AC, Ingelsson E, Sundstrom J, Carrero JJ, Gustafsson S, Feldreich T, Stenemo M, Larsson A, Lind L, Arnlov J (2017). Use of proteomics to investigate kidney function decline over 5 years. Clin J Am Soc Nephrol.

[CR8] Chauhan K, Verghese DA, Rao V, Chan L, Parikh CR, Coca SG, Nadkarni GN (2019). Plasma endostatin predicts kidney outcomes in patients with type 2 diabetes. Kidney Int.

[CR9] Choquet H, Yin J, Jacobson AS, Horton BH, Hoffmann TJ, Jorgenson E, Avins AL, Pressman AR (2021). New and sex-specific migraine susceptibility loci identified from a multiethnic genome-wide meta-analysis. Commun Biol.

[CR10] Cullell N, Gallego-Fabrega C, Carcel-Marquez J, Muino E, Llucia-Carol L, Lledos M, Martin-Campos JM, Molina J, Casas L, Almeria M, Fernandez-Cadenas I, Krupinski J (2022). ICA1L is associated with small vessel disease: a proteome-wide association study in small vessel stroke and intracerebral haemorrhage. Int J Mol Sci.

[CR11] Evangelou E, Warren HR, Mosen-Ansorena D, Mifsud B, Pazoki R, Gao H, Ntritsos G, Dimou N, Cabrera CP, Karaman I, Ng FL, Evangelou M, Witkowska K, Tzanis E, Hellwege JN, Giri A, Velez Edwards DR, Sun YV, Cho K, Gaziano JM, Wilson PWF, Tsao PS, Kovesdy CP, Esko T, Magi R, Milani L, Almgren P, Boutin T, Debette S, Ding J, Giulianini F, Holliday EG, Jackson AU, Li-Gao R, Lin WY, Luan J, Mangino M, Oldmeadow C, Prins BP, Qian Y, Sargurupremraj M, Shah N, Surendran P, Theriault S, Verweij N, Willems SM, Zhao JH, Amouyel P, Connell J, de Mutsert R, Doney ASF, Farrall M, Menni C, Morris AD, Noordam R, Pare G, Poulter NR, Shields DC, Stanton A, Thom S, Abecasis G, Amin N, Arking DE, Ayers KL, Barbieri CM, Batini C, Bis JC, Blake T, Bochud M, Boehnke M, Boerwinkle E, Boomsma DI, Bottinger EP, Braund PS, Brumat M, Campbell A, Campbell H, Chakravarti A, Chambers JC, Chauhan G, Ciullo M, Cocca M, Collins F, Cordell HJ, Davies G, de Borst MH, de Geus EJ, Deary IJ, Deelen J, Del Greco MF, Demirkale CY, Dorr M, Ehret GB, Elosua R, Enroth S, Erzurumluoglu AM, Ferreira T, Franberg M, Franco OH, Gandin I (2018). Genetic analysis of over 1 million people identifies 535 new loci associated with blood pressure traits. Nat Genet.

[CR12] Gasparini CF, Smith RA, Griffiths LR (2016). Genetic insights into migraine and glutamate: a protagonist driving the headache. J Neurol Sci.

[CR13] Giambartolomei C, Vukcevic D, Schadt EE, Franke L, Hingorani AD, Wallace C, Plagnol V (2014). Bayesian test for colocalisation between pairs of genetic association studies using summary statistics. PLoS Genet.

[CR14] Gormley P, Anttila V, Winsvold BS, Palta P, Esko T, Pers TH, Farh KH, Cuenca-Leon E, Muona M, Furlotte NA, Kurth T, Ingason A, McMahon G, Ligthart L, Terwindt GM, Kallela M, Freilinger TM, Ran C, Gordon SG, Stam AH, Steinberg S, Borck G, Koiranen M, Quaye L, Adams HH, Lehtimaki T, Sarin AP, Wedenoja J, Hinds DA, Buring JE, Schurks M, Ridker PM, Hrafnsdottir MG, Stefansson H, Ring SM, Hottenga JJ, Penninx BW, Farkkila M, Artto V, Kaunisto M, Vepsalainen S, Malik R, Heath AC, Madden PA, Martin NG, Montgomery GW, Kurki MI, Kals M, Magi R, Parn K, Hamalainen E, Huang H, Byrnes AE, Franke L, Huang J, Stergiakouli E, Lee PH, Sandor C, Webber C, Cader Z, Muller-Myhsok B, Schreiber S, Meitinger T, Eriksson JG, Salomaa V, Heikkila K, Loehrer E, Uitterlinden AG, Hofman A, van Duijn CM, Cherkas L, Pedersen LM, Stubhaug A, Nielsen CS, Mannikko M, Mihailov E, Milani L, Gobel H, Esserlind AL, Christensen AF, Hansen TF, Werge T, Kaprio J, Aromaa AJ, Raitakari O, Ikram MA, Spector T, Jarvelin MR, Metspalu A, Kubisch C, Strachan DP, Ferrari MD, Belin AC, Dichgans M, Wessman M, van den Maagdenberg AM, Zwart JA, Boomsma DI, Smith GD, International Headache Genetics C (2016). Meta-analysis of 375,000 individuals identifies 38 susceptibility loci for migraine. Nat Genet.

[CR15] Gulati A, Bale AE, Dykas DJ, Bia MJ, Danovitch GM, Moeckel GW, Somlo S, Dahl NK (2018). TREX1 mutation causing autosomal dominant thrombotic microangiopathy and CKD-A novel presentation. Am J Kidney Dis.

[CR16] Guo Y, Rist PM, Daghlas I, Giulianini F, Kurth T, Chasman DI, International Headache Genetics C, Me Research T (2020). A genome-wide cross-phenotype meta-analysis of the association of blood pressure with migraine. Nat Commun.

[CR17] Gusev A, Ko A, Shi H, Bhatia G, Chung W, Penninx BW, Jansen R, de Geus EJ, Boomsma DI, Wright FA, Sullivan PF, Nikkola E, Alvarez M, Civelek M, Lusis AJ, Lehtimaki T, Raitoharju E, Kahonen M, Seppala I, Raitakari OT, Kuusisto J, Laakso M, Price AL, Pajukanta P, Pasaniuc B (2016). Integrative approaches for large-scale transcriptome-wide association studies. Nat Genet.

[CR18] Haberle J, Burlina A, Chakrapani A, Dixon M, Karall D, Lindner M, Mandel H, Martinelli D, Pintos-Morell G, Santer R, Skouma A, Servais A, Tal G, Rubio V, Huemer M, Dionisi-Vici C (2019). Suggested guidelines for the diagnosis and management of urea cycle disorders: first revision. J Inherit Metab Dis.

[CR19] Hautakangas H, Winsvold BS, Ruotsalainen SE, Bjornsdottir G, Harder AVE, Kogelman LJA, Thomas LF, Noordam R, Benner C, Gormley P, Artto V, Banasik K, Bjornsdottir A, Boomsma DI, Brumpton BM, Burgdorf KS, Buring JE, Chalmer MA, de Boer I, Dichgans M, Erikstrup C, Farkkila M, Garbrielsen ME, Ghanbari M, Hagen K, Happola P, Hottenga JJ, Hrafnsdottir MG, Hveem K, Johnsen MB, Kahonen M, Kristoffersen ES, Kurth T, Lehtimaki T, Lighart L, Magnusson SH, Malik R, Pedersen OB, Pelzer N, Penninx B, Ran C, Ridker PM, Rosendaal FR, Sigurdardottir GR, Skogholt AH, Sveinsson OA, Thorgeirsson TE, Ullum H, Vijfhuizen LS, Widen E, van Dijk KW, Headache HA-I, Aromaa A, Belin AC, Freilinger T, Ikram MA, Jarvelin MR, Raitakari OT, Terwindt GM, Kallela M, Wessman M, Olesen J, Chasman DI, Nyholt DR, Stefansson H, Stefansson K, van den Maagdenberg A, Hansen TF, Ripatti S, Zwart JA, Palotie A, Pirinen M, International Headache Genetics C, Danish Blood Donor Study Genomic C (2022). Genome-wide analysis of 102,084 migraine cases identifies 123 risk loci and subtype-specific risk alleles. Nat Genet.

[CR20] Hemani G, Zheng J, Elsworth B, Wade KH, Haberland V, Baird D, Laurin C, Burgess S, Bowden J, Langdon R, Tan VY, Yarmolinsky J, Shihab HA, Timpson NJ, Evans DM, Relton C, Martin RM, Davey Smith G, Gaunt TR, Haycock PC (2018). The MR-base platform supports systematic causal inference across the human phenome. Elife.

[CR21] Huang C, Chen M, Pang D, Bi D, Zou Y, Xia X, Yang W, Luo L, Deng R, Tan H, Zhou L, Yu S, Guo L, Du X, Cui Y, Hu J, Mao Q, Worley PF, Xiao B (2014). Developmental and activity-dependent expression of LanCL1 confers antioxidant activity required for neuronal survival. Dev Cell.

[CR22] Huang H, Fang M, Jostins L, Umicevic Mirkov M, Boucher G, Anderson CA, Andersen V, Cleynen I, Cortes A, Crins F, D'Amato M, Deffontaine V, Dmitrieva J, Docampo E, Elansary M, Farh KK, Franke A, Gori AS, Goyette P, Halfvarson J, Haritunians T, Knight J, Lawrance IC, Lees CW, Louis E, Mariman R, Meuwissen T, Mni M, Momozawa Y, Parkes M, Spain SL, Theatre E, Trynka G, Satsangi J, van Sommeren S, Vermeire S, Xavier RJ, Weersma RK, Duerr RH, Mathew CG, Rioux JD, McGovern DPB, Cho JH, Georges M, Daly MJ, Barrett JC, International Inflammatory Bowel Disease Genetics C (2017). Fine-mapping inflammatory bowel disease loci to single-variant resolution. Nature.

[CR23] Kottgen A, Pattaro C, Boger CA, Fuchsberger C, Olden M, Glazer NL, Parsa A, Gao X, Yang Q, Smith AV, O'Connell JR, Li M, Schmidt H, Tanaka T, Isaacs A, Ketkar S, Hwang SJ, Johnson AD, Dehghan A, Teumer A, Pare G, Atkinson EJ, Zeller T, Lohman K, Cornelis MC, Probst-Hensch NM, Kronenberg F, Tonjes A, Hayward C, Aspelund T, Eiriksdottir G, Launer LJ, Harris TB, Rampersaud E, Mitchell BD, Arking DE, Boerwinkle E, Struchalin M, Cavalieri M, Singleton A, Giallauria F, Metter J, de Boer IH, Haritunians T, Lumley T, Siscovick D, Psaty BM, Zillikens MC, Oostra BA, Feitosa M, Province M, de Andrade M, Turner ST, Schillert A, Ziegler A, Wild PS, Schnabel RB, Wilde S, Munzel TF, Leak TS, Illig T, Klopp N, Meisinger C, Wichmann HE, Koenig W, Zgaga L, Zemunik T, Kolcic I, Minelli C, Hu FB, Johansson A, Igl W, Zaboli G, Wild SH, Wright AF, Campbell H, Ellinghaus D, Schreiber S, Aulchenko YS, Felix JF, Rivadeneira F, Uitterlinden AG, Hofman A, Imboden M, Nitsch D, Brandstatter A, Kollerits B, Kedenko L, Magi R, Stumvoll M, Kovacs P, Boban M, Campbell S, Endlich K, Volzke H, Kroemer HK, Nauck M, Volker U, Polasek O, Vitart V (2010). New loci associated with kidney function and chronic kidney disease. Nat Genet.

[CR24] Leipold E, Liebmann L, Korenke GC, Heinrich T, Giesselmann S, Baets J, Ebbinghaus M, Goral RO, Stodberg T, Hennings JC, Bergmann M, Altmuller J, Thiele H, Wetzel A, Nurnberg P, Timmerman V, De Jonghe P, Blum R, Schaible HG, Weis J, Heinemann SH, Hubner CA, Kurth I (2013). A de novo gain-of-function mutation in SCN11A causes loss of pain perception. Nat Genet.

[CR25] Lin X, Yi Z, Zhang X, Liu Q, Zhang H, Cai R, Chen C, Zhang H, Zhao P, Pan P (2021). Retinal nerve fiber layer changes in migraine: a systematic review and meta-analysis. Neurol Sci.

[CR26] Mobasheran P, Rajai N, Kohansal P, Dehpour AR, Shafaroodi H (2020). The effects of acute sumatriptan treatment on renal ischemia/reperfusion injury in rat and the possible involvement of nitric oxide. Can J Physiol Pharmacol.

[CR27] Morrison J, Knoblauch N, Marcus JH, Stephens M, He X (2020). Mendelian randomization accounting for correlated and uncorrelated pleiotropic effects using genome-wide summary statistics. Nat Genet.

[CR28] Murray AM (2009). The brain and the kidney connection: a model of accelerated vascular cognitive impairment. Neurology.

[CR29] Ng CYH, Tan BYQ, Teo YN, Teo YH, Syn NLX, Leow AST, Ho JSY, Chan MY, Wong RCC, Chai P, Chan ACY, Sharma VK, Yeo LLL, Sia CH, Ong JJY (2022). Myocardial infarction, stroke and cardiovascular mortality among migraine patients: a systematic review and meta-analysis. J Neurol.

[CR30] O'Brown ZK, Van Nostrand EL, Higgins JP, Kim SK (2015). The inflammatory transcription factors NFkappaB, STAT1 and STAT3 drive age-associated transcriptional changes in the human kidney. PLoS Genet.

[CR31] Ou YN, Yang YX, Deng YT, Zhang C, Hu H, Wu BS, Liu Y, Wang YJ, Zhu Y, Suckling J, Tan L, Yu JT (2021). Identification of novel drug targets for Alzheimer's disease by integrating genetics and proteomes from brain and blood. Mol Psychiatry.

[CR32] Pickrell JK, Berisa T, Liu JZ, Segurel L, Tung JY, Hinds DA (2016). Detection and interpretation of shared genetic influences on 42 human traits. Nat Genet.

[CR33] Pierce BL, Ahsan H, Vanderweele TJ (2011). Power and instrument strength requirements for Mendelian randomization studies using multiple genetic variants. Int J Epidemiol.

[CR34] Raina R, Bedoyan JK, Lichter-Konecki U, Jouvet P, Picca S, Mew NA, Machado MC, Chakraborty R, Vemuganti M, Grewal MK, Bunchman T, Sethi SK, Krishnappa V, McCulloch M, Alhasan K, Bagga A, Basu RK, Schaefer F, Filler G, Warady BA (2020). Consensus guidelines for management of hyperammonaemia in paediatric patients receiving continuous kidney replacement therapy. Nat Rev Nephrol.

[CR35] Reuter U, Chiarugi A, Bolay H, Moskowitz MA (2002). Nuclear factor-kappaB as a molecular target for migraine therapy. Ann Neurol.

[CR36] Seliger SL, Longstreth WT (2008). Lessons about brain vascular disease from another pulsating organ, the kidney. Stroke.

[CR37] Shim H, Chasman DI, Smith JD, Mora S, Ridker PM, Nickerson DA, Krauss RM, Stephens M (2015). A multivariate genome-wide association analysis of 10 LDL subfractions, and their response to statin treatment, in 1868 Caucasians. PLoS ONE.

[CR38] Siewert KM, Klarin D, Damrauer SM, Chang KM, Tsao PS, Assimes TL, Davey Smith G, Voight BF, The International Headache Genetics C (2020). Cross-trait analyses with migraine reveal widespread pleiotropy and suggest a vascular component to migraine headache. Int J Epidemiol.

[CR39] Sudlow C, Gallacher J, Allen N, Beral V, Burton P, Danesh J, Downey P, Elliott P, Green J, Landray M, Liu B, Matthews P, Ong G, Pell J, Silman A, Young A, Sprosen T, Peakman T, Collins R (2015). UK biobank: an open access resource for identifying the causes of a wide range of complex diseases of middle and old age. PLoS Med.

[CR40] Sutherland HG, Griffiths LR (2017). Genetics of migraine: insights into the molecular basis of migraine disorders. Headache.

[CR41] Teumer A, Li Y, Ghasemi S, Prins BP, Wuttke M, Hermle T, Giri A, Sieber KB, Qiu C, Kirsten H, Tin A, Chu AY, Bansal N, Feitosa MF, Wang L, Chai JF, Cocca M, Fuchsberger C, Gorski M, Hoppmann A, Horn K, Li M, Marten J, Noce D, Nutile T, Sedaghat S, Sveinbjornsson G, Tayo BO, van der Most PJ, Xu Y, Yu Z, Gerstner L, Arnlov J, Bakker SJL, Baptista D, Biggs ML, Boerwinkle E, Brenner H, Burkhardt R, Carroll RJ, Chee ML, Chee ML, Chen M, Cheng CY, Cook JP, Coresh J, Corre T, Danesh J, de Borst MH, De Grandi A, de Mutsert R, de Vries APJ, Degenhardt F, Dittrich K, Divers J, Eckardt KU, Ehret G, Endlich K, Felix JF, Franco OH, Franke A, Freedman BI, Freitag-Wolf S, Gansevoort RT, Giedraitis V, Gogele M, Grundner-Culemann F, Gudbjartsson DF, Gudnason V, Hamet P, Harris TB, Hicks AA, Holm H, Foo VHX, Hwang SJ, Ikram MA, Ingelsson E, Jaddoe VWV, Jakobsdottir J, Josyula NS, Jung B, Kahonen M, Khor CC, Kiess W, Koenig W, Korner A, Kovacs P, Kramer H, Kramer BK, Kronenberg F, Lange LA, Langefeld CD, Lee JJ, Lehtimaki T, Lieb W, Lim SC, Lind L, Lindgren CM, Liu J, Loeffler M (2019). Genome-wide association meta-analyses and fine-mapping elucidate pathways influencing albuminuria. Nat Commun.

[CR42] Thomas CA, Tejwani L, Trujillo CA, Negraes PD, Herai RH, Mesci P, Macia A, Crow YJ, Muotri AR (2017). Modeling of TREX1-dependent autoimmune disease using human stem cells highlights L1 accumulation as a source of neuroinflammation. Cell Stem Cell.

[CR43] Tietjen GE (2009). Migraine as a systemic vasculopathy. Cephalalgia.

[CR44] Weng SC, Wu CL, Kor CT, Chiu PF, Wu MJ, Chang CC, Tarng DC (2017). Migraine and subsequent chronic kidney disease risk: a nationwide population-based cohort study. BMJ Open.

[CR45] Wuttke M, Li Y, Li M, Sieber KB, Feitosa MF, Gorski M, Tin A, Wang L, Chu AY, Hoppmann A, Kirsten H, Giri A, Chai JF, Sveinbjornsson G, Tayo BO, Nutile T, Fuchsberger C, Marten J, Cocca M, Ghasemi S, Xu Y, Horn K, Noce D, van der Most PJ, Sedaghat S, Yu Z, Akiyama M, Afaq S, Ahluwalia TS, Almgren P, Amin N, Arnlov J, Bakker SJL, Bansal N, Baptista D, Bergmann S, Biggs ML, Biino G, Boehnke M, Boerwinkle E, Boissel M, Bottinger EP, Boutin TS, Brenner H, Brumat M, Burkhardt R, Butterworth AS, Campana E, Campbell A, Campbell H, Canouil M, Carroll RJ, Catamo E, Chambers JC, Chee ML, Chee ML, Chen X, Cheng CY, Cheng Y, Christensen K, Cifkova R, Ciullo M, Concas MP, Cook JP, Coresh J, Corre T, Sala CF, Cusi D, Danesh J, Daw EW, de Borst MH, De Grandi A, de Mutsert R, de Vries APJ, Degenhardt F, Delgado G, Demirkan A, Di Angelantonio E, Dittrich K, Divers J, Dorajoo R, Eckardt KU, Ehret G, Elliott P, Endlich K, Evans MK, Felix JF, Foo VHX, Franco OH, Franke A, Freedman BI, Freitag-Wolf S, Friedlander Y, Froguel P, Gansevoort RT, Gao H, Gasparini P, Gaziano JM, Giedraitis V, Gieger C (2019). A catalog of genetic loci associated with kidney function from analyses of a million individuals. Nat Genet.

[CR46] Yazgan Y, Naziroglu M (2021). Involvement of TRPM2 in the neurobiology of experimental migraine: focus on oxidative stress and apoptosis. Mol Neurobiol.

[CR47] Zhang Y, Lu Q, Ye Y, Huang K, Liu W, Wu Y, Zhong X, Li B, Yu Z, Travers BG, Werling DM, Li JJ, Zhao H (2021). SUPERGNOVA: local genetic correlation analysis reveals heterogeneous etiologic sharing of complex traits. Genome Biol.

[CR48] Zheng J, Zhang Y, Rasheed H, Walker V, Sugawara Y, Li J, Leng Y, Elsworth B, Wootton RE, Fang S, Yang Q, Burgess S, Haycock PC, Borges MC, Cho Y, Carnegie R, Howell A, Robinson J, Thomas LF, Brumpton BM, Hveem K, Hallan S, Franceschini N, Morris AP, Kottgen A, Pattaro C, Wuttke M, Yamamoto M, Kashihara N, Akiyama M, Kanai M, Matsuda K, Kamatani Y, Okada Y, Walters R, Millwood IY, Chen Z, Davey Smith G, Barbour S, Yu C, Asvold BO, Zhang H, Gaunt TR (2022). Trans-ethnic Mendelian-randomization study reveals causal relationships between cardiometabolic factors and chronic kidney disease. Int J Epidemiol.

[CR49] Zhu X, Feng T, Tayo BO, Liang J, Young JH, Franceschini N, Smith JA, Yanek LR, Sun YV, Edwards TL, Chen W, Nalls M, Fox E, Sale M, Bottinger E, Rotimi C, Consortium CB, Liu Y, McKnight B, Liu K, Arnett DK, Chakravati A, Cooper RS, Redline S (2015). Meta-analysis of correlated traits via summary statistics from GWASs with an application in hypertension. Am J Hum Genet.

[CR50] Zhu Z, Hasegawa K, Camargo CA, Liang L (2021). Investigating asthma heterogeneity through shared and distinct genetics: Insights from genome-wide cross-trait analysis. J Allergy Clin Immunol.

