# Supplementary material for: Migraine, chronic kidney disease and kidney function: observational and genetic analyses
Source: Hum Genet. 2023 Jun 12;142(8):1185–200. doi: 10.1007/s00439-023-02575-9 (PMC10449948; doi:10.1007/s00439-023-02575-9)
Supplement: Supplementary file 1 — Supplementary file1 (PDF 2309 KB) [file 439_2023_2575_MOESM1_ESM.pdf]

Supplementary materials

Supplementary Figure 1. Mendelian randomization analysis between migraine subtypes and chronic kidney disease.

Supplementary Table 1. Characteristics of genetic instruments of migraine and their effect sizes with chronic kidney disease.

Supplementary Table 2. Characteristics of genetic instruments of migraine and their effect sizes with estimated glomerular filtration rate.

Supplementary Table 3. Characteristics of genetic instruments of migraine and their effect sizes with urinary albumin-to-creatinine ratio.

Supplementary Table 4. Characteristics of genetic instruments of chronic kidney disease and their effect sizes with migraine.

Supplementary Table 5. Characteristics of genetic instruments of estimated glomerular filtration rate and their effect sizes with migraine.

Supplementary Table 6. Characteristics of genetic instruments of urinary albumin-to-creatinine ratio and their effect sizes with migraine.

Supplementary Table 7. Data sources, sample sizes, number of instruments and F-statistics.

Supplementary Table 8. Baseline Characteristics of UK Biobank participants by migraine status at the baseline.

Supplementary Table 9. Local heritability of migraine and estimated glomerular filtration rate, and regions that contribute significant genetic correlation as estimated by SUPERGNOVA ($P < 0.05/2353$).

Supplementary Table 10. Results from cross-trait meta-analysis of migraine and chronic kidney disease (SNPs with P-CPASSOC $< 5\times10^{-8}$ and single trait P-value $< 1\times10^{-5}$ are shown).

Supplementary Table 11. Previously reported genome-wide significant variants for migraine and chronic kidney disease among European ancestry.

Supplementary Table 12. Detailed annotation of genome-wide significant SNPs identified by cross-trait meta-analysis.

Supplementary Table 13. Fine-mapping 99% credible-set of index SNP from cross-trait meta-analysis between migraine and chronic kidney disease.

Supplementary Table 14. Colocalization analysis of index SNPs from cross-trait meta-analysis between migraine and chronic kidney disease.

Supplementary Table 15. Multivariable Mendelian randomization analysis between migraine, blood pressure, and urinary albumin-to-creatinine ratio.

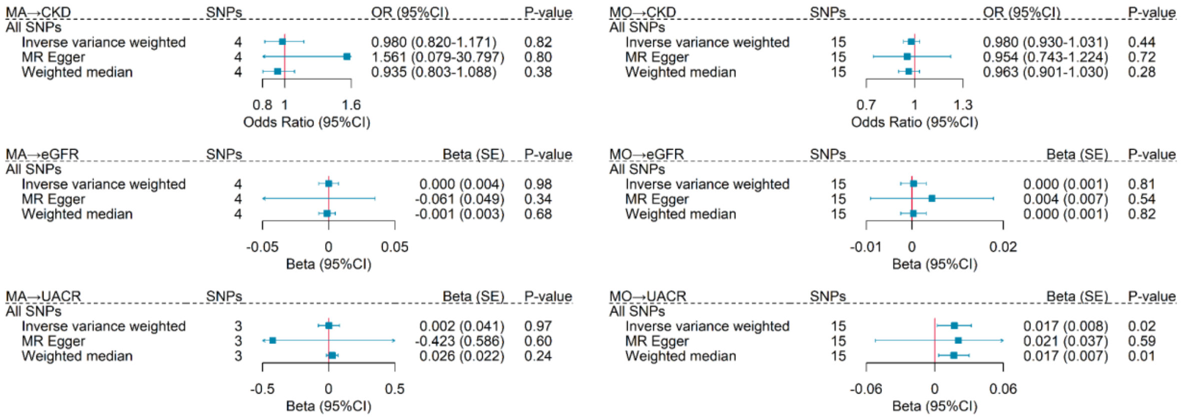

Supplementary Figure 1. Mendelian randomization analysis between migraine subtypes and chronic kidney disease. The blue boxes denote point estimate of the causal effects, and the error bars denote 95% confidence intervals (CI). MA, migraine with aura; MO, migraine without aura; CKD, chronic kidney disease; eGFR, estimated glomerular filtration rate; UACR, urinary albumin-to-creatinine ratio.

Supplementary Table 1. Characteristics of genetic instruments of migraine and their effect sizes with chronic kidney disease.

SNP	CHR	BP	A1	A2	EAF	Exposure			Outcome			Pleiotropic traits
						Beta	SE	P-value	Beta	SE	P-value	
rs1003194	11	15126085	A	G	0.383044	0.034346	0.00542	2.43E-10	-0.0085	0.01	0.3959	
rs10038882	5	145752008	T	C	0.748493	0.042486	0.005987	1.33E-12	0.0021	0.0113	0.8505	
rs10128028	1	7055843	T	C	0.518501	0.029818	0.005159	7.66E-09	0.002	0.0091	0.8289	
rs10156578	9	29372501	C	G	0.432923	0.036622	0.005256	3.34E-12	0.0113	0.0098	0.248	
rs10166942	2	234825093	T	C	0.805077	0.099232	0.006619	9.35E-51	0.0038	0.0121	0.756	
rs1019990	18	44866736	C	T	0.704318	0.038687	0.00568	1E-11	0.003	0.0099	0.7657	
rs10218452	1	3075597	G	A	0.228718	0.110043	0.00618	7.26E-71	0.0029	0.0125	0.8157	
rs10234636	7	40427617	T	C	0.110988	0.088941	0.00809	4.43E-28	-0.0179	0.0148	0.227	
rs10405121	19	13339128	G	A	0.548175	0.033232	0.005332	4.74E-10	-0.0044	0.0099	0.6584	
rs10456100	6	39183470	T	C	0.280916	0.050686	0.005726	9.16E-19	-0.0158	0.0102	0.1219	Coronary artery disease
rs10777902	12	98498223	A	C	0.497816	0.03305	0.005134	1.25E-10	-0.007	0.0091	0.4445	
rs10828247	10	21822856	G	A	0.347206	0.034003	0.005879	7.51E-09	0.0096	0.0115	0.4049	
rs10866704	5	176676461	A	T	0.738677	0.037919	0.006762	2.1E-08	0.0122	0.0132	0.3544	
rs10894756	11	133745852	G	A	0.57297	0.02947	0.005304	2.83E-08	-0.0118	0.0097	0.2247	
rs11031122	11	30547438	C	T	0.242951	0.036645	0.005937	6.91E-10	0.0153	0.0108	0.1575	
rs11153082	6	97059666	G	A	0.330752	0.084038	0.005436	7.26E-54	-0.0152	0.0097	0.1171	
rs11165300	1	92177663	G	T	0.238911	0.032899	0.006019	4.72E-08	0.01	0.0106	0.3439	
rs11172113	12	57527283	T	C	0.585472	0.106774	0.005289	1.38E-90	-0.0107	0.0098	0.2733	Pulmonary function (smoking interaction), Cervical artery dissection, Peak expiratory flow, Medication use (anilides), Coronary artery disease, Medication use (antimigraine preparations), Lung function (FEV1/FVC), Headache
rs11248546	10	125242283	C	T	0.563664	0.036787	0.005202	1.59E-12	0.0126	0.0092	0.1706	
rs11578492	1	60529980	C	A	0.42011	0.031385	0.005398	6.25E-09	-0.0074	0.0112	0.5084	
rs11624776	14	93595591	A	C	0.683572	0.04984	0.005635	9.75E-19	0.0025	0.0106	0.8159	Thyroid hormone levels
rs11652860	17	46632679	G	C	0.5082	0.031226	0.005456	1.07E-08	0.0014	0.0097	0.8884	
rs11782789	8	27266287	A	T	0.836279	0.041373	0.006972	3.03E-09	0.0239	0.0124	0.05423	
rs11957829	5	121515195	G	A	0.17189	0.041098	0.006803	1.58E-09	0.01	0.0134	0.4554	Ischemic stroke, Stroke, Ischemic stroke (small-vessel)
rs12057629	1	15538493	C	T	0.356375	0.04	0.005365	9.38E-14	0.0056	0.0101	0.5787	
rs12226331	11	102070976	T	A	0.34776	0.039628	0.005384	1.92E-13	0.0072	0.0102	0.4804	
rs12260159	10	100702737	G	A	0.924845	0.083238	0.010313	7.33E-16	-0.0074	0.0201	0.7133	
rs12260436	10	104741114	C	A	0.256544	0.035997	0.005839	7.29E-10	0.0084	0.0102	0.4092	Worry

rs12295710	11	3249984	T	C	0.47141	0.044736	0.005465	2.86E-16	0.0066	0.0094	0.4835	
rs12452590	17	60720058	G	T	0.375475	0.037525	0.005897	2.03E-10	0.0039	0.0107	0.7122	Lung function (FVC), Heel bone mineral density
rs1245463	14	27661650	A	G	0.388029	0.039621	0.005269	5.72E-14	-0.0004	0.0094	0.9656	
rs12598836	16	4534482	G	A	0.304153	0.037775	0.005948	2.21E-10	0.0109	0.0122	0.3715	
rs12653216	5	81129663	T	C	0.21193	0.037031	0.006416	8.08E-09	-0.0133	0.0112	0.2324	
rs12708529	15	81022364	A	G	0.72977	0.03575	0.005815	8.11E-10	0.0058	0.0109	0.5959	
rs12712881	2	43649780	A	C	0.434536	0.032774	0.005219	3.5E-10	-0.0074	0.0097	0.4456	
rs1271309	12	124820705	G	A	0.835968	0.040283	0.007315	3.74E-08	0.0059	0.0134	0.6577	Diastolic blood pressure, Male-pattern baldness
rs12787928	11	61697078	A	T	0.482089	0.030484	0.005257	6.85E-09	0.0077	0.0092	0.4012	
rs1285294	17	77925681	C	T	0.615671	0.030049	0.005482	4.32E-08	0.0205	0.011	0.06241	
rs13078967	3	154289946	A	C	0.973267	0.145952	0.017759	2.16E-16	-0.027	0.0293	0.3565	
rs13235543	7	73013901	C	T	0.873257	0.058115	0.007964	3.06E-13	-0.0077	0.0139	0.5803	
rs138556413	2	203832867	C	T	0.963567	0.129319	0.015887	4.15E-16	-0.0167	0.0267	0.5322	
rs1458170	12	41901277	C	T	0.844283	0.041557	0.00713	5.75E-09	0.0011	0.0126	0.9294	
rs1472662	1	39590409	T	G	0.215808	0.035213	0.006245	1.75E-08	0.0139	0.0116	0.2302	
rs1499963	3	124607055	C	T	0.679113	0.032139	0.005556	7.48E-09	0.0182	0.0103	0.07713	
rs1542668	14	42548912	G	A	0.671485	0.030724	0.00551	2.53E-08	0.0071	0.0097	0.4651	
rs17723637	9	109687403	G	A	0.149827	0.041475	0.007201	8.63E-09	-0.0117	0.0127	0.3565	
rs1982072	19	41864509	A	T	0.700223	0.037194	0.005634	4.22E-11	-0.0009	0.01	0.9259	
rs2000660	13	110788441	A	G	0.092377	0.049566	0.009083	4.95E-08	0.0247	0.0172	0.1503	
rs2078371	1	115677183	C	T	0.117396	0.106181	0.007819	5.87E-42	-0.0001	0.0143	0.9956	
rs2119930	17	47514039	G	T	0.405274	0.04088	0.005244	6.69E-15	0.0151	0.0099	0.1286	
rs2160875	12	4527322	C	T	0.477419	0.064863	0.005153	2.72E-36	-0.0108	0.0097	0.2643	
rs2274224	10	96039597	G	C	0.560933	0.055292	0.005217	3.28E-26	0.005	0.0092	0.5898	Body fat percentage
rs2274319	1	156450873	T	C	0.347186	0.072653	0.005395	2.74E-41	0.0131	0.0096	0.1718	
rs246326	5	122306398	T	C	0.125645	0.047567	0.007702	6.8E-10	0.002	0.0145	0.8906	
rs2672592	10	124230750	T	G	0.362778	0.038031	0.00535	1.22E-12	0.0056	0.01	0.576	
rs28451064	21	35593827	G	A	0.86898	0.063261	0.008031	3.52E-15	0.0216	0.0147	0.1409	Coronary artery disease, Pulse pressure, Myocardial infarction, Heel bone mineral density, Waist-hip ratio
rs28455731	6	121846038	T	G	0.157289	0.068955	0.007014	8.82E-23	0.0066	0.0124	0.5945	
rs28739509	1	38366907	C	T	0.272555	0.038592	0.006103	2.64E-10	-0.0093	0.0108	0.3877	
rs28756401	14	58761912	G	A	0.715445	0.033494	0.005764	6.4E-09	-0.0138	0.0102	0.1755	
rs28929474	14	94844947	T	C	0.019367	0.110981	0.018611	2.54E-09	-0.0286	0.0396	0.4709	Height, Glucagon levels in response to oral glucose tolerance test (fasting), Post bronchodilator FEV1/FVC ratio, Blood protein levels, Breast size, Alcohol consumption (drinks per

													week), Fat-free mass, Post bronchodilator percent predicted FEV1 in smoking, Antineutrophil cytoplasmic antibody-associated vasculitis, Systolic blood pressure, Alanine transaminase levels, Heel bone mineral density, Metabolite levels (small molecules and protein measures), Gallstone disease
rs34273564	6	72321017	T	C	0.480865	0.033603	0.005192	1E-10	0.0029	0.0096	0.7661	
rs34914463	17	7366619	T	C	0.870518	0.04919	0.008237	2.41E-09	-0.01	0.0157	0.5242	Heel bone mineral density
rs3891689	9	119258583	C	T	0.234046	0.057673	0.006072	2.28E-21	-0.023	0.0115	0.04423	
rs4278223	9	140743200	T	A	0.636303	0.045144	0.007294	6.24E-10	-0.0252	0.0123	0.04125	
rs42854	5	74963277	G	C	0.311652	0.039387	0.005513	9.4E-13	-0.0018	0.0098	0.8509	
rs4668251	2	171234235	G	C	0.709559	0.033415	0.005779	7.58E-09	0.0056	0.0107	0.6032	
rs4705403	5	149380493	A	G	0.103683	0.047078	0.00825	1.18E-08	0.0053	0.0155	0.7298	
rs4739105	8	64496159	T	C	0.210995	0.035642	0.006416	2.85E-08	-0.0156	0.0121	0.1976	
rs4814864	20	19469817	C	G	0.254392	0.064808	0.005842	1.44E-28	-0.0098	0.0104	0.3479	
rs4842676	12	90091782	C	G	0.81683	0.041025	0.006858	2.26E-09	0.0081	0.0125	0.5201	Mean arterial pressure x alcohol consumption (light vs heavy) interaction (2df test)
rs4907224	2	96576609	A	T	0.675732	0.035813	0.005933	1.63E-09	0.0437	0.011	7.71E-05	
rs4910165	11	10674044	G	C	0.677735	0.0568	0.005534	1.09E-24	0.0009	0.0102	0.9319	Respiratory diseases
rs55707505	14	75362552	T	C	0.675137	0.03071	0.005505	2.48E-08	0.0112	0.0098	0.2564	
rs56067931	7	120481569	C	T	0.799583	0.035535	0.006506	4.83E-08	0.01	0.0114	0.3809	
rs56140113	1	206843108	C	T	0.780053	0.036807	0.00637	7.76E-09	0.0085	0.0111	0.4443	
rs566673	11	66401373	G	T	0.461609	0.030245	0.005259	9.07E-09	-0.0077	0.0091	0.3962	Insomnia
rs580845	9	14103618	A	C	0.600745	0.029933	0.00546	4.3E-08	0.0018	0.01	0.8556	
rs6057599	20	31168439	T	C	0.335051	0.04127	0.005529	8.73E-14	0.0125	0.0102	0.2183	
rs625686	22	20142932	C	T	0.306442	0.034071	0.005908	8.26E-09	-0.0052	0.0105	0.6187	
rs6556059	5	172645766	T	C	0.362528	0.033553	0.005459	8.16E-10	0.0029	0.0102	0.7787	Infantile hypertrophic pyloric stenosis
rs6568677	6	111713302	A	G	0.209439	0.035211	0.006278	2.09E-08	-0.006	0.0112	0.5883	
rs6668908	1	186913055	G	T	0.669233	0.030687	0.005482	2.22E-08	0.0011	0.0098	0.9138	
rs6693567	1	150510660	C	T	0.268579	0.043464	0.00586	1.25E-13	0.0092	0.0104	0.3766	
rs6795209	3	88210464	A	G	0.191251	0.041261	0.007239	1.23E-08	-0.0042	0.0151	0.7801	
rs7034179	9	71746838	T	C	0.428569	0.043065	0.005217	1.6E-16	0.0199	0.0092	0.03138	
rs72764846	1	245847455	G	A	0.783771	0.037521	0.006426	5.41E-09	0.0055	0.0119	0.6421	
rs72923449	2	176978383	C	A	0.035696	0.077728	0.014215	4.66E-08	0.1448	0.0267	5.66E-08	
rs73138150	3	86149109	T	A	0.31676	0.032618	0.005803	1.95E-08	-0.0021	0.0105	0.8433	

rs7335684	13	47193696	G	A	0.246703	0.034248	0.00598	1.05E-08	-0.0106	0.0106	0.3204	
rs7371912	3	30472786	A	G	0.302852	0.043988	0.005685	1.06E-14	0.0021	0.0121	0.8592	
rs73805934	4	35469918	G	C	0.822895	0.041945	0.006879	1.11E-09	0.0129	0.012	0.2829	
rs74182632	19	19406126	A	G	0.054841	0.063801	0.011245	1.43E-08	-0.0179	0.0203	0.3784	
rs74434374	6	31850308	C	A	0.94897	0.073666	0.012552	4.52E-09	-0.0143	0.02	0.4761	
rs75002882	14	76496477	G	T	0.987756	0.15695	0.027301	9.22E-09	0.0707	0.05	0.1567	
rs7506921	18	20201527	A	T	0.532407	0.038986	0.005742	1.17E-11	0.0181	0.011	0.09997	
rs7511672	1	66178918	G	A	0.538291	0.031258	0.005161	1.43E-09	0.0123	0.0092	0.1835	
rs7564469	2	145258445	C	T	0.160786	0.041248	0.007051	5.06E-09	-0.0009	0.0132	0.9456	
rs7618883	3	48498456	T	A	0.457585	0.02845	0.005184	4.16E-08	-0.0269	0.0096	0.005242	
rs764508	21	36935896	C	T	0.369935	0.031505	0.00532	3.28E-09	-0.0105	0.0095	0.2658	
rs7684253	4	57727311	T	C	0.550329	0.039211	0.005187	4.21E-14	-0.0051	0.0092	0.5824	
rs7916911	10	8722944	T	G	0.283965	0.039808	0.005707	3.18E-12	-0.0155	0.0102	0.1283	
rs7932866	11	46548094	A	G	0.836281	0.043133	0.00722	2.38E-09	0.0026	0.0123	0.8337	Body mass index
rs7996252	13	78876537	T	C	0.595889	0.028861	0.005257	4.11E-08	0.0041	0.0093	0.6586	
rs8046696	16	75442143	T	G	0.427658	0.04115	0.005455	4.76E-14	-0.0006	0.0099	0.9513	Coronary artery disease
rs8052831	16	87578039	G	A	0.344377	0.04257	0.005479	8.25E-15	-0.0059	0.0097	0.5455	
rs8077768	17	78256432	C	T	0.47684	0.039696	0.005555	9.32E-13	-0.0189	0.01	0.0597	
rs8087942	18	55192245	A	G	0.666961	0.039215	0.005492	9.71E-13	0.0296	0.0097	0.002371	
rs843215	2	156416638	G	A	0.467709	0.028679	0.005149	2.61E-08	0.0125	0.0091	0.1719	
rs869432	10	112502662	A	C	0.583777	0.029294	0.00531	3.54E-08	-0.0081	0.0098	0.4058	
rs895219	2	146037564	C	T	0.300106	0.036982	0.005587	3.74E-11	-0.0146	0.0105	0.1668	
rs910187	20	45841052	G	A	0.628264	0.034898	0.005408	1.14E-10	0.0133	0.0095	0.1601	Depressive symptoms, Insomnia, Neuroticism, Well-being spectrum (multivariate analysis)
rs9295536	6	22131929	C	A	0.561698	0.035501	0.005184	7.75E-12	-0.0099	0.0093	0.2862	Neuroblastoma
rs9349379	6	12903957	A	G	0.590207	0.077238	0.005328	1.41E-47	0.0203	0.0099	0.0409	Systolic blood pressure, Pulse pressure, Hypertension, Coronary artery disease, Headache, Alcohol consumption (drinks per week), Cervical artery dissection, Medication use (antimigraine preparations), Diastolic blood pressure, Myocardial infarction, Estimated glomerular filtration rate
rs9383843	6	150133954	C	A	0.647085	0.033172	0.005468	1.35E-09	0.0198	0.0101	0.05033	
rs9468830	6	30749712	T	G	0.706336	0.035668	0.006385	2.38E-08	0.0033	0.0105	0.7517	
rs950570	3	80302512	T	C	0.070819	0.056693	0.009963	1.3E-08	0.0059	0.0184	0.7474	
rs9894634	17	1967501	C	T	0.403078	0.033879	0.00523	9.64E-11	-0.019	0.0093	0.0404	Estimated glomerular filtration rate

rs56019088	1	73891226	I	D	0.48364	0.045189	0.006295	7.32E-13
rs11487328	1	174601659	G	C	0.745764	0.048358	0.008568	1.7E-08
rs200314499	10	134479675	D	I	0.339944	0.044514	0.006484	6.92E-12
rs111404218	20	10684159	G	C	0.336878	0.043633	0.006857	2.04E-10
rs1507220	X	34102712	A	C	0.301482	0.027764	0.004988	2.67E-08
rs4403550	X	40746484	T	C	0.711795	0.030532	0.005147	3.07E-09

SNP, single nucleotide polymorphisms; CHR, chromosome; BP, physical position of SNP (base-pairs); A1, effect allele; A2, alternative allele; EAF: effect allele frequency; Beta, effect allele beta coefficient.

Supplementary Table 2. Characteristics of genetic instruments of migraine and their effect sizes with estimated glomerular filtration rate.

SNP	CHR	BP	A1	A2	EAF	Exposure			Outcome			Pleiotropic traits
						Beta	SE	P-value	Beta	SE	P-value	
rs1003194	11	15126085	A	G	0.383044	0.034346	0.00542	2.43E-10	-0.00015	0.000368	0.6813	
rs10038882	5	145752008	T	C	0.748493	0.042486	0.005987	1.33E-12	-1.3E-05	0.000406	0.9743	
rs10128028	1	7055843	T	C	0.518501	0.029818	0.005159	7.66E-09	0.000473	0.000343	0.1683	
rs10156578	9	29372501	C	G	0.432923	0.036622	0.005256	3.34E-12	-0.00069	0.000356	0.05139	
rs10166942	2	234825093	T	C	0.805077	0.099232	0.006619	9.35E-51	-0.00071	0.000446	0.1139	
rs1019990	18	44866736	C	T	0.704318	0.038687	0.00568	1E-11	-0.00066	0.000374	0.07918	
rs10218452	1	3075597	G	A	0.228718	0.110043	0.00618	7.26E-71	-0.00051	0.000435	0.2448	
rs10234636	7	40427617	T	C	0.110988	0.088941	0.00809	4.43E-28	0.000001	0.00055	0.9983	
rs10405121	19	13339128	G	A	0.548175	0.033232	0.005332	4.74E-10	0.000598	0.000359	0.09591	
rs10456100	6	39183470	T	C	0.280916	0.050686	0.005726	9.16E-19	-0.00029	0.000387	0.4526	Coronary artery disease
rs10777902	12	98498223	A	C	0.497816	0.03305	0.005134	1.25E-10	0.000182	0.000343	0.5967	
rs10828247	10	21822856	G	A	0.347206	0.034003	0.005879	7.51E-09	0.001239	0.000436	0.004479	
rs10866704	5	176676461	A	T	0.738677	0.037919	0.006762	2.1E-08	-0.0006	0.000518	0.2463	
rs10894756	11	133745852	G	A	0.57297	0.02947	0.005304	2.83E-08	0.000584	0.000354	0.09876	
rs11031122	11	30547438	C	T	0.242951	0.036645	0.005937	6.91E-10	-0.00054	0.000414	0.1908	
rs11153082	6	97059666	G	A	0.330752	0.084038	0.005436	7.26E-54	0.000108	0.000367	0.7675	
rs11165300	1	92177663	G	T	0.238911	0.032899	0.006019	4.72E-08	-0.00013	0.000404	0.7529	
rs11172113	12	57527283	T	C	0.585472	0.106774	0.005289	1.38E-90	0.000758	0.000363	0.03667	Pulmonary function (smoking interaction), Cervical artery dissection, Peak expiratory flow, Medication use (anilides), Coronary artery disease, Medication use (antimigraine preparations), Lung function (FEV1/FVC), Headache
rs11248546	10	125242283	C	T	0.563664	0.036787	0.005202	1.59E-12	-0.00062	0.000373	0.09706	
rs11578492	1	60529980	C	A	0.42011	0.031385	0.005398	6.25E-09	0.000284	0.000435	0.5142	
rs11624776	14	93595591	A	C	0.683572	0.04984	0.005635	9.75E-19	-0.00121	0.000388	0.001796	Thyroid hormone levels
rs11652860	17	46632679	G	C	0.5082	0.031226	0.005456	1.07E-08	-0.00048	0.000357	0.1809	
rs11782789	8	27266287	A	T	0.836279	0.041373	0.006972	3.03E-09	0.000171	0.000461	0.7105	
rs11957829	5	121515195	G	A	0.17189	0.041098	0.006803	1.58E-09	-0.00031	0.000476	0.5145	Ischemic stroke, Stroke, Ischemic stroke (small-vessel)

rs12057629	1	15538493	C	T	0.356375	0.04	0.005365	9.38E-14	-0.00063	0.000366	0.08449	
rs12226331	11	102070976	T	A	0.34776	0.039628	0.005384	1.92E-13	-0.00023	0.00037	0.5419	
rs12260159	10	100702737	G	A	0.924845	0.083238	0.010313	7.33E-16	0.00027	0.000793	0.7337	
rs12260436	10	104741114	C	A	0.256544	0.035997	0.005839	7.29E-10	-0.00082	0.000388	0.03427	Worry
rs12295710	11	3249984	T	C	0.47141	0.044736	0.005465	2.86E-16	0.000324	0.000356	0.3622	
rs12452590	17	60720058	G	T	0.375475	0.037525	0.005897	2.03E-10	-5.8E-05	0.000392	0.882	Lung function (FVC), Heel bone mineral density
rs1245463	14	27661650	A	G	0.388029	0.039621	0.005269	5.72E-14	0.00039	0.000354	0.2711	
rs12598836	16	4534482	G	A	0.304153	0.037775	0.005948	2.21E-10	-0.00063	0.000468	0.1761	
rs12653216	5	81129663	T	C	0.21193	0.037031	0.006416	8.08E-09	0.000995	0.000423	0.0187	
rs12708529	15	81022364	A	G	0.72977	0.03575	0.005815	8.11E-10	-0.00031	0.000393	0.4378	
rs12712881	2	43649780	A	C	0.434536	0.032774	0.005219	3.5E-10	0.001128	0.000352	0.001331	
rs1271309	12	124820705	G	A	0.835968	0.040283	0.007315	3.74E-08	0.000035	0.000486	0.9431	Diastolic blood pressure, Male-pattern baldness
rs12787928	11	61697078	A	T	0.482089	0.030484	0.005257	6.85E-09	-6E-06	0.000346	0.9856	
rs1285294	17	77925681	C	T	0.615671	0.030049	0.005482	4.32E-08	-0.00022	0.000412	0.5904	
rs13078967	3	154289946	A	C	0.973267	0.145952	0.017759	2.16E-16	-0.00111	0.0011	0.3138	
rs13235543	7	73013901	C	T	0.873257	0.058115	0.007964	3.06E-13	0.001041	0.00052	0.04526	
rs138556413	2	203832867	C	T	0.963567	0.129319	0.015887	4.15E-16	0.001529	0.00099	0.1223	
rs1458170	12	41901277	C	T	0.844283	0.041557	0.00713	5.75E-09	-0.00082	0.00047	0.08057	
rs1472662	1	39590409	T	G	0.215808	0.035213	0.006245	1.75E-08	-0.00122	0.000425	0.004031	
rs1499963	3	124607055	C	T	0.679113	0.032139	0.005556	7.48E-09	-0.00095	0.000374	0.01148	
rs1542668	14	42548912	G	A	0.671485	0.030724	0.00551	2.53E-08	-0.00017	0.000367	0.6537	
rs17723637	9	109687403	G	A	0.149827	0.041475	0.007201	8.63E-09	0.000116	0.000481	0.8089	
rs1982072	19	41864509	A	T	0.700223	0.037194	0.005634	4.22E-11	0.000152	0.000382	0.6909	
rs2000660	13	110788441	A	G	0.092377	0.049566	0.009083	4.95E-08	-0.00033	0.000635	0.6009	
rs2078371	1	115677183	C	T	0.117396	0.106181	0.007819	5.87E-42	0.000807	0.000535	0.131	
rs2119930	17	47514039	G	T	0.405274	0.04088	0.005244	6.69E-15	0.000113	0.000361	0.7539	
rs2160875	12	4527322	C	T	0.477419	0.064863	0.005153	2.72E-36	-0.00046	0.000352	0.1941	
rs2274224	10	96039597	G	C	0.560933	0.055292	0.005217	3.28E-26	0.000436	0.000348	0.2096	Body fat percentage
rs2274319	1	156450873	T	C	0.347186	0.072653	0.005395	2.74E-41	-0.00127	0.00036	0.000434	
rs246326	5	122306398	T	C	0.125645	0.047567	0.007702	6.8E-10	-0.00067	0.000524	0.2021	
rs2672592	10	124230750	T	G	0.362778	0.038031	0.00535	1.22E-12	0.000426	0.000367	0.2449	
rs28451064	21	35593827	G	A	0.86898	0.063261	0.008031	3.52E-15	-0.00012	0.000529	0.8142	Coronary artery disease, Pulse pressure, Myocardial infarction, Heel bone mineral density, Waist-hip ratio
rs28455731	6	121846038	T	G	0.157289	0.068955	0.007014	8.82E-23	0.00045	0.000477	0.346	
rs28739509	1	38366907	C	T	0.272555	0.038592	0.006103	2.64E-10	-0.00007	0.0004	0.8607	

rs28756401	14	58761912	G	A	0.715445	0.033494	0.005764	6.4E-09	0.000599	0.000382	0.117	
rs28929474	14	94844947	T	C	0.019367	0.110981	0.018611	2.54E-09	-0.00104	0.001338	0.4381	Height, Glucagon levels in response to oral glucose tolerance test (fasting), Post bronchodilator FEV1/FVC ratio, Blood protein levels, Breast size, Alcohol consumption (drinks per week), Fat-free mass, Post bronchodilator percent predicted FEV1 in smoking, Antineutrophil cytoplasmic antibody-associated vasculitis, Systolic blood pressure, Alanine transaminase levels, Heel bone mineral density, Metabolite levels (small molecules and protein measures), Gallstone disease
rs34273564	6	72321017	T	C	0.480865	0.033603	0.005192	1E-10	-3.3E-05	0.000351	0.9253	
rs34914463	17	7366619	T	C	0.870518	0.04919	0.008237	2.41E-09	0.000659	0.00056	0.2396	Heel bone mineral density
rs3891689	9	119258583	C	T	0.234046	0.057673	0.006072	2.28E-21	0.001351	0.000415	0.001129	
rs4278223	9	140743200	T	A	0.636303	0.045144	0.007294	6.24E-10	0.001566	0.00044	0.000375	
rs42854	5	74963277	G	C	0.311652	0.039387	0.005513	9.4E-13	0.000683	0.000368	0.06371	
rs4668251	2	171234235	G	C	0.709559	0.033415	0.005779	7.58E-09	-0.00027	0.000391	0.4913	
rs4705403	5	149380493	A	G	0.103683	0.047078	0.00825	1.18E-08	0.000941	0.000573	0.1002	
rs4739105	8	64496159	T	C	0.210995	0.035642	0.006416	2.85E-08	-0.00025	0.000439	0.5647	
rs4814864	20	19469817	C	G	0.254392	0.064808	0.005842	1.44E-28	-0.00017	0.000393	0.6686	
rs4842676	12	90091782	C	G	0.81683	0.041025	0.006858	2.26E-09	-0.00077	0.000533	0.149	Mean arterial pressure x alcohol consumption (light vs heavy) interaction (2df test)
rs4907224	2	96576609	A	T	0.675732	0.035813	0.005933	1.63E-09	-0.00116	0.000401	0.003939	
rs4910165	11	10674044	G	C	0.677735	0.0568	0.005534	1.09E-24	0.00004	0.000373	0.9142	Respiratory diseases
rs55707505	14	75362552	T	C	0.675137	0.03071	0.005505	2.48E-08	0.000106	0.000366	0.7723	
rs56067931	7	120481569	C	T	0.799583	0.035535	0.006506	4.83E-08	0.000472	0.000428	0.2704	
rs56140113	1	206843108	C	T	0.780053	0.036807	0.00637	7.76E-09	-0.00042	0.000418	0.3129	
rs566673	11	66401373	G	T	0.461609	0.030245	0.005259	9.07E-09	0.000876	0.00035	0.01232	Insomnia

rs580845	9	14103618	A	C	0.600745	0.029933	0.00546	4.3E-08	-0.00075	0.000365	0.03889	
rs6057599	20	31168439	T	C	0.335051	0.04127	0.005529	8.73E-14	-0.0006	0.000374	0.108	
rs625686	22	20142932	C	T	0.306442	0.034071	0.005908	8.26E-09	-0.00027	0.000388	0.4954	
rs6556059	5	172645766	T	C	0.362528	0.033553	0.005459	8.16E-10	-2.1E-05	0.00037	0.9539	Infantile hypertrophic pyloric stenosis
rs6568677	6	111713302	A	G	0.209439	0.035211	0.006278	2.09E-08	-0.00054	0.000419	0.1942	
rs6668908	1	186913055	G	T	0.669233	0.030687	0.005482	2.22E-08	0.001028	0.000365	0.004864	
rs6693567	1	150510660	C	T	0.268579	0.043464	0.00586	1.25E-13	-0.00206	0.000398	2.35E-07	
rs7034179	9	71746838	T	C	0.428569	0.043065	0.005217	1.6E-16	-0.00063	0.000355	0.07412	
rs72764846	1	245847455	G	A	0.783771	0.037521	0.006426	5.41E-09	-0.00023	0.000436	0.6054	
rs72923449	2	176978383	C	A	0.035696	0.077728	0.014215	4.66E-08	-0.00665	0.000958	3.85E-12	
rs73138150	3	86149109	T	A	0.31676	0.032618	0.005803	1.95E-08	0.000018	0.000385	0.9633	
rs7335684	13	47193696	G	A	0.246703	0.034248	0.00598	1.05E-08	0.000189	0.000401	0.638	
rs7371912	3	30472786	A	G	0.302852	0.043988	0.005685	1.06E-14	-0.00028	0.000463	0.5473	
rs73805934	4	35469918	G	C	0.822895	0.041945	0.006879	1.11E-09	-0.00099	0.000448	0.02658	
rs74182632	19	19406126	A	G	0.054841	0.063801	0.011245	1.43E-08	0.000687	0.000767	0.3707	
rs74434374	6	31850308	C	A	0.94897	0.073666	0.012552	4.52E-09	0.000274	0.0008	0.7322	
rs75002882	14	76496477	G	T	0.987756	0.15695	0.027301	9.22E-09	-0.00181	0.001718	0.293	
rs7506921	18	20201527	A	T	0.532407	0.038986	0.005742	1.17E-11	-0.00062	0.000442	0.1626	
rs7511672	1	66178918	G	A	0.538291	0.031258	0.005161	1.43E-09	-0.00113	0.000347	0.001118	
rs7564469	2	145258445	C	T	0.160786	0.041248	0.007051	5.06E-09	0.000801	0.000492	0.1035	
rs7618883	3	48498456	T	A	0.457585	0.02845	0.005184	4.16E-08	0.00227	0.000352	1.18E-10	
rs764508	21	36935896	C	T	0.369935	0.031505	0.00532	3.28E-09	-0.00028	0.000358	0.4356	
rs7684253	4	57727311	T	C	0.550329	0.039211	0.005187	4.21E-14	-3.5E-05	0.000348	0.919	
rs7916911	10	8722944	T	G	0.283965	0.039808	0.005707	3.18E-12	-0.00108	0.000382	0.004867	
rs7932866	11	46548094	A	G	0.836281	0.043133	0.00722	2.38E-09	-0.00111	0.000466	0.01729	Body mass index
rs7996252	13	78876537	T	C	0.595889	0.028861	0.005257	4.11E-08	0.00009	0.000353	0.7977	
rs8046696	16	75442143	T	G	0.427658	0.04115	0.005455	4.76E-14	-0.00037	0.000361	0.3064	Coronary artery disease
rs8052831	16	87578039	G	A	0.344377	0.04257	0.005479	8.25E-15	0.000405	0.000367	0.2696	
rs8077768	17	78256432	C	T	0.47684	0.039696	0.005555	9.32E-13	0.000759	0.000366	0.03799	
rs8087942	18	55192245	A	G	0.666961	0.039215	0.005492	9.71E-13	-0.00088	0.000362	0.01474	
rs843215	2	156416638	G	A	0.467709	0.028679	0.005149	2.61E-08	0.000041	0.000344	0.9055	
rs869432	10	112502662	A	C	0.583777	0.029294	0.00531	3.54E-08	0.000062	0.000358	0.8635	
rs895219	2	146037564	C	T	0.300106	0.036982	0.005587	3.74E-11	0.00108	0.000385	0.004993	
rs910187	20	45841052	G	A	0.628264	0.034898	0.005408	1.14E-10	-0.00064	0.000358	0.07458	Depressive symptoms, Insomnia, Neuroticism, Well-being spectrum (multivariate analysis)
rs9295536	6	22131929	C	A	0.561698	0.035501	0.005184	7.75E-12	0.000047	0.000356	0.8941	Neuroblastoma
rs9349379	6	12903957	A	G	0.590207	0.077238	0.005328	1.41E-47	-0.0004	0.000367	0.2754	Systolic blood pressure, Pulse pressure, Hypertension,

												Coronary artery disease, Headache, Alcohol consumption (drinks per week), Cervical artery dissection, Medication use (antimigraine preparations), Diastolic blood pressure, Myocardial infarction, Estimated glomerular filtration rate
rs9383843	6	150133954	C	A	0.647085	0.033172	0.005468	1.35E-09	-0.00151	0.000369	4.46E-05	
rs9468830	6	30749712	T	G	0.706336	0.035668	0.006385	2.38E-08	-9.4E-05	0.000406	0.8162	
rs950570	3	80302512	T	C	0.070819	0.056693	0.009963	1.3E-08	0.000507	0.00078	0.5157	
rs9894634	17	1967501	C	T	0.403078	0.033879	0.00523	9.64E-11	0.002107	0.00035	1.69E-09	Estimated glomerular filtration rate
rs56019088	1	73891226	I	D	0.48364	0.045189	0.006295	7.32E-13				
rs11487328	1	174601659	G	C	0.745764	0.048358	0.008568	1.7E-08				
rs6795209	3	88210464	A	G	0.191251	0.041261	0.007239	1.23E-08				
rs200314499	10	134479675	D	I	0.339944	0.044514	0.006484	6.92E-12				
rs111404218	20	10684159	G	C	0.336878	0.043633	0.006857	2.04E-10				
rs1507220	X	34102712	A	C	0.301482	0.027764	0.004988	2.67E-08				
rs4403550	X	40746484	T	C	0.711795	0.030532	0.005147	3.07E-09				

SNP, single nucleotide polymorphisms; CHR, chromosome; BP, physical position of SNP (base-pairs); A1, effect allele; A2, alternative allele; EAF: effect allele frequency; Beta, effect allele beta coefficient.

Supplementary Table 3. Characteristics of genetic instruments of migraine and their effect sizes with urinary albumin-to-creatinine ratio.

SNP	CHR	BP	A1	A2	EAF	Exposure			Outcome			Pleiotropic traits
						Beta	SE	P-value	Beta	SE	P-value	
rs1003194	11	15126085	A	G	0.383044	0.034346	0.00542	2.43E-10	-0.00182	0.002096	0.3847	
rs10038882	5	145752008	T	C	0.748493	0.042486	0.005987	1.33E-12	0.002287	0.00231	0.3223	
rs10128028	1	7055843	T	C	0.518501	0.029818	0.005159	7.66E-09	-0.00118	0.001987	0.5534	
rs10156578	9	29372501	C	G	0.432923	0.036622	0.005256	3.34E-12	0.004192	0.002037	0.0396	
rs10166942	2	234825093	T	C	0.805077	0.099232	0.006619	9.35E-51	0.000456	0.002534	0.8573	
rs1019990	18	44866736	C	T	0.704318	0.038687	0.00568	1E-11	0.004183	0.002177	0.05465	
rs10218452	1	3075597	G	A	0.228718	0.110043	0.00618	7.26E-71	0.00201	0.00243	0.4082	
rs10234636	7	40427617	T	C	0.110988	0.088941	0.00809	4.43E-28	-0.00246	0.003232	0.4472	
rs10405121	19	13339128	G	A	0.548175	0.033232	0.005332	4.74E-10	0.002722	0.002043	0.1828	
rs10456100	6	39183470	T	C	0.280916	0.050686	0.005726	9.16E-19	-0.00858	0.002205	9.98E-05	Coronary artery disease
rs10777902	12	98498223	A	C	0.497816	0.03305	0.005134	1.25E-10	0.001367	0.001984	0.491	
rs10828247	10	21822856	G	A	0.347206	0.034003	0.005879	7.51E-09	-0.00113	0.007026	0.872	
rs10894756	11	133745852	G	A	0.57297	0.02947	0.005304	2.83E-08	0.000731	0.002041	0.7204	
rs11031122	11	30547438	C	T	0.242951	0.036645	0.005937	6.91E-10	-0.00705	0.002312	0.002301	
rs11153082	6	97059666	G	A	0.330752	0.084038	0.005436	7.26E-54	0.00232	0.002129	0.2759	
rs11165300	1	92177663	G	T	0.238911	0.032899	0.006019	4.72E-08	0.002703	0.00233	0.2459	
rs11172113	12	57527283	T	C	0.585472	0.106774	0.005289	1.38E-90	0.004425	0.002057	0.03141	Pulmonary function (smoking interaction), Cervical artery dissection, Peak expiratory flow, Medication use (anilides), Coronary artery disease, Medication use (antimigraine preparations), Lung function (FEV1/FVC), Headache
rs11248546	10	125242283	C	T	0.563664	0.036787	0.005202	1.59E-12	-0.00215	0.002013	0.2863	
rs11624776	14	93595591	A	C	0.683572	0.04984	0.005635	9.75E-19	-3E-05	0.002189	0.9891	Thyroid hormone levels
rs11652860	17	46632679	G	C	0.5082	0.031226	0.005456	1.07E-08	-0.00621	0.002034	0.002276	
rs11782789	8	27266287	A	T	0.836279	0.041373	0.006972	3.03E-09	-0.00251	0.002689	0.35	
rs11957829	5	121515195	G	A	0.17189	0.041098	0.006803	1.58E-09	0.000417	0.002665	0.8757	Ischemic stroke, Stroke, Ischemic stroke (small-vessel)
rs12057629	1	15538493	C	T	0.356375	0.04	0.005365	9.38E-14	0.000291	0.002093	0.8893	
rs12226331	11	102070976	T	A	0.34776	0.039628	0.005384	1.92E-13	0.001075	0.002116	0.6115	
rs12260159	10	100702737	G	A	0.924845	0.083238	0.010313	7.33E-16	0.006166	0.010338	0.5509	
rs12260436	10	104741114	C	A	0.256544	0.035997	0.005839	7.29E-10	-0.00046	0.002275	0.8407	Worry
rs12295710	11	3249984	T	C	0.47141	0.044736	0.005465	2.86E-16	0.000135	0.002011	0.9465	

rs12452590	17	60720058	G	T	0.375475	0.037525	0.005897	2.03E-10	-3.9E-05	0.002165	0.9855	Lung function (FVC), Heel bone mineral density
rs1245463	14	27661650	A	G	0.388029	0.039621	0.005269	5.72E-14	0.001252	0.002039	0.5393	
rs12653216	5	81129663	T	C	0.21193	0.037031	0.006416	8.08E-09	0.000524	0.00246	0.8315	
rs12708529	15	81022364	A	G	0.72977	0.03575	0.005815	8.11E-10	-0.00072	0.002259	0.7513	
rs12712881	2	43649780	A	C	0.434536	0.032774	0.005219	3.5E-10	0.005306	0.002033	0.009053	
rs1271309	12	124820705	G	A	0.835968	0.040283	0.007315	3.74E-08	0.006942	0.002741	0.01131	Diastolic blood pressure, Male-pattern baldness
rs12787928	11	61697078	A	T	0.482089	0.030484	0.005257	6.85E-09	0.000558	0.001991	0.7794	
rs1285294	17	77925681	C	T	0.615671	0.030049	0.005482	4.32E-08	0.002704	0.002196	0.2183	
rs13078967	3	154289946	A	C	0.973267	0.145952	0.017759	2.16E-16	0.002307	0.006746	0.7323	
rs13235543	7	73013901	C	T	0.873257	0.058115	0.007964	3.06E-13	0.007925	0.002978	0.00778	
rs138556413	2	203832867	C	T	0.963567	0.129319	0.015887	4.15E-16	0.016281	0.00542	0.002665	
rs1458170	12	41901277	C	T	0.844283	0.041557	0.00713	5.75E-09	0.002625	0.002709	0.3325	
rs1472662	1	39590409	T	G	0.215808	0.035213	0.006245	1.75E-08	-0.00751	0.002451	0.002178	
rs1499963	3	124607055	C	T	0.679113	0.032139	0.005556	7.48E-09	0.001297	0.002157	0.5475	
rs1542668	14	42548912	G	A	0.671485	0.030724	0.00551	2.53E-08	-0.00196	0.002113	0.3534	
rs17723637	9	109687403	G	A	0.149827	0.041475	0.007201	8.63E-09	-0.00265	0.002797	0.3429	
rs1982072	19	41864509	A	T	0.700223	0.037194	0.005634	4.22E-11	0.000479	0.002202	0.8279	
rs2000660	13	110788441	A	G	0.092377	0.049566	0.009083	4.95E-08	0.002159	0.003553	0.5433	
rs2078371	1	115677183	C	T	0.117396	0.106181	0.007819	5.87E-42	0.001024	0.003103	0.7414	
rs2119930	17	47514039	G	T	0.405274	0.04088	0.005244	6.69E-15	0.002493	0.002075	0.2295	
rs2160875	12	4527322	C	T	0.477419	0.064863	0.005153	2.72E-36	0.001156	0.002022	0.5674	
rs2274224	10	96039597	G	C	0.560933	0.055292	0.005217	3.28E-26	0.001662	0.002001	0.4061	Body fat percentage
rs2274319	1	156450873	T	C	0.347186	0.072653	0.005395	2.74E-41	0.003909	0.00209	0.06142	
rs246326	5	122306398	T	C	0.125645	0.047567	0.007702	6.8E-10	-0.00048	0.00306	0.8751	
rs2672592	10	124230750	T	G	0.362778	0.038031	0.00535	1.22E-12	0.000103	0.002104	0.9611	
rs28451064	21	35593827	G	A	0.86898	0.063261	0.008031	3.52E-15	0.005789	0.00306	0.05856	Coronary artery disease, Pulse pressure, Myocardial infarction, Heel bone mineral density, Waist-hip ratio
rs28455731	6	121846038	T	G	0.157289	0.068955	0.007014	8.82E-23	-0.00075	0.002749	0.7841	
rs28739509	1	38366907	C	T	0.272555	0.038592	0.006103	2.64E-10	0.000785	0.002241	0.7262	
rs28756401	14	58761912	G	A	0.715445	0.033494	0.005764	6.4E-09	-0.00347	0.002204	0.1158	
rs28929474	14	94844947	T	C	0.019367	0.110981	0.018611	2.54E-09	0.028928	0.007301	7.43E-05	Height, Glucagon levels in response to oral glucose tolerance test (fasting), Post bronchodilator FEV1/FVC ratio, Blood protein levels, Breast size, Alcohol consumption (drinks per week), Fat-free mass, Post bronchodilator percent

												predicted FEV1 in smoking, Antineutrophil cytoplasmic antibody-associated vasculitis, Systolic blood pressure, Alanine transaminase levels, Heel bone mineral density, Metabolite levels (small molecules and protein measures), Gallstone disease
rs34273564	6	72321017	T	C	0.480865	0.033603	0.005192	1E-10	2.65E-05	0.002026	0.9896	
rs34914463	17	7366619	T	C	0.870518	0.04919	0.008237	2.41E-09	0.007183	0.003028	0.01769	Heel bone mineral density
rs3891689	9	119258583	C	T	0.234046	0.057673	0.006072	2.28E-21	0.002169	0.002381	0.3623	
rs4278223	9	140743200	T	A	0.636303	0.045144	0.007294	6.24E-10	0.007604	0.0022	0.000548	
rs42854	5	74963277	G	C	0.311652	0.039387	0.005513	9.4E-13	0.004376	0.002142	0.04109	
rs4668251	2	171234235	G	C	0.709559	0.033415	0.005779	7.58E-09	-0.00193	0.002227	0.3859	
rs4705403	5	149380493	A	G	0.103683	0.047078	0.00825	1.18E-08	-0.0063	0.00322	0.05044	
rs4739105	8	64496159	T	C	0.210995	0.035642	0.006416	2.85E-08	-0.00386	0.002484	0.12	
rs4814864	20	19469817	C	G	0.254392	0.064808	0.005842	1.44E-28	0.004181	0.002275	0.06604	
rs4842676	12	90091782	C	G	0.81683	0.041025	0.006858	2.26E-09	0.005778	0.002649	0.02919	Mean arterial pressure x alcohol consumption (light vs heavy) interaction (2df test)
rs4907224	2	96576609	A	T	0.675732	0.035813	0.005933	1.63E-09	-0.00104	0.002214	0.6393	
rs4910165	11	10674044	G	C	0.677735	0.0568	0.005534	1.09E-24	0.009571	0.00216	9.4E-06	Respiratory diseases
rs55707505	14	75362552	T	C	0.675137	0.03071	0.005505	2.48E-08	-0.00256	0.002119	0.2271	
rs56067931	7	120481569	C	T	0.799583	0.035535	0.006506	4.83E-08	-0.00312	0.002501	0.2126	
rs56140113	1	206843108	C	T	0.780053	0.036807	0.00637	7.76E-09	-0.00214	0.002389	0.3704	
rs566673	11	66401373	G	T	0.461609	0.030245	0.005259	9.07E-09	0.000243	0.002005	0.9034	Insomnia
rs580845	9	14103618	A	C	0.600745	0.029933	0.00546	4.3E-08	-8.4E-05	0.002076	0.9677	
rs6057599	20	31168439	T	C	0.335051	0.04127	0.005529	8.73E-14	0.00586	0.002158	0.006608	
rs625686	22	20142932	C	T	0.306442	0.034071	0.005908	8.26E-09	0.002371	0.002224	0.2863	
rs6556059	5	172645766	T	C	0.362528	0.033553	0.005459	8.16E-10	-0.00015	0.002107	0.9449	Infantile hypertrophic pyloric stenosis
rs6568677	6	111713302	A	G	0.209439	0.035211	0.006278	2.09E-08	0.001565	0.002465	0.5254	
rs6668908	1	186913055	G	T	0.669233	0.030687	0.005482	2.22E-08	0.000282	0.002102	0.8933	
rs6693567	1	150510660	C	T	0.268579	0.043464	0.00586	1.25E-13	0.003463	0.002258	0.125	
rs7034179	9	71746838	T	C	0.428569	0.043065	0.005217	1.6E-16	0.001815	0.002009	0.3663	
rs72764846	1	245847455	G	A	0.783771	0.037521	0.006426	5.41E-09	0.004075	0.002468	0.09873	
rs72923449	2	176978383	C	A	0.035696	0.077728	0.014215	4.66E-08	-0.02015	0.005354	0.000167	
rs73138150	3	86149109	T	A	0.31676	0.032618	0.005803	1.95E-08	0.004713	0.002183	0.0308	
rs7335684	13	47193696	G	A	0.246703	0.034248	0.00598	1.05E-08	-0.00099	0.002312	0.6682	
rs73805934	4	35469918	G	C	0.822895	0.041945	0.006879	1.11E-09	0.003637	0.00257	0.157	

rs74182632	19	19406126	A	G	0.054841	0.063801	0.011245	1.43E-08	-0.00166	0.00442	0.7068	
rs74434374	6	31850308	C	A	0.94897	0.073666	0.012552	4.52E-09	0.004144	0.004805	0.3884	
rs75002882	14	76496477	G	T	0.987756	0.15695	0.027301	9.22E-09	0.006591	0.009234	0.4754	
rs7511672	1	66178918	G	A	0.538291	0.031258	0.005161	1.43E-09	-0.0003	0.001989	0.8815	
rs7564469	2	145258445	C	T	0.160786	0.041248	0.007051	5.06E-09	0.008867	0.002817	0.001647	
rs7618883	3	48498456	T	A	0.457585	0.02845	0.005184	4.16E-08	-0.0012	0.002021	0.5518	
rs764508	21	36935896	C	T	0.369935	0.031505	0.00532	3.28E-09	-0.0022	0.002061	0.2862	
rs7684253	4	57727311	T	C	0.550329	0.039211	0.005187	4.21E-14	-0.00553	0.002005	0.005852	
rs7916911	10	8722944	T	G	0.283965	0.039808	0.005707	3.18E-12	0.003101	0.002195	0.1578	
rs7932866	11	46548094	A	G	0.836281	0.043133	0.00722	2.38E-09	-0.00859	0.002659	0.00123	Body mass index
rs7996252	13	78876537	T	C	0.595889	0.028861	0.005257	4.11E-08	-0.00368	0.002023	0.06922	
rs8046696	16	75442143	T	G	0.427658	0.04115	0.005455	4.76E-14	0.001594	0.002063	0.4398	Coronary artery disease
rs8052831	16	87578039	G	A	0.344377	0.04257	0.005479	8.25E-15	0.001276	0.002105	0.5444	
rs8077768	17	78256432	C	T	0.47684	0.039696	0.005555	9.32E-13	0.002223	0.002031	0.2737	
rs8087942	18	55192245	A	G	0.666961	0.039215	0.005492	9.71E-13	0.001856	0.002109	0.3788	
rs843215	2	156416638	G	A	0.467709	0.028679	0.005149	2.61E-08	0.003681	0.001992	0.06465	
rs869432	10	112502662	A	C	0.583777	0.029294	0.00531	3.54E-08	-0.00112	0.002055	0.5863	
rs895219	2	146037564	C	T	0.300106	0.036982	0.005587	3.74E-11	0.003788	0.002199	0.08493	
rs910187	20	45841052	G	A	0.628264	0.034898	0.005408	1.14E-10	-0.00169	0.002055	0.4106	Depressive symptoms, Insomnia, Neuroticism, Well-being spectrum (multivariate analysis)
rs9295536	6	22131929	C	A	0.561698	0.035501	0.005184	7.75E-12	-0.00199	0.002029	0.3268	Neuroblastoma
rs9349379	6	12903957	A	G	0.590207	0.077238	0.005328	1.41E-47	0.001556	0.002067	0.4516	Systolic blood pressure, Pulse pressure, Hypertension, Coronary artery disease, Headache, Alcohol consumption (drinks per week), Cervical artery dissection, Medication use (antimigraine preparations), Diastolic blood pressure, Myocardial infarction
rs9383843	6	150133954	C	A	0.647085	0.033172	0.005468	1.35E-09	0.002012	0.002113	0.3411	
rs9468830	6	30749712	T	G	0.706336	0.035668	0.006385	2.38E-08	-0.00125	0.002246	0.5776	
rs950570	3	80302512	T	C	0.070819	0.056693	0.009963	1.3E-08	-0.00255	0.003718	0.4934	
rs9894634	17	1967501	C	T	0.403078	0.033879	0.00523	9.64E-11	0.005646	0.002024	0.005275	Estimated glomerular filtration rate
rs11578492	1	60529980	C	A	0.42011	0.031385	0.005398	6.25E-09				
rs56019088	1	73891226	I	D	0.48364	0.045189	0.006295	7.32E-13				
rs11487328	1	174601659	G	C	0.745764	0.048358	0.008568	1.7E-08				
rs7371912	3	30472786	A	G	0.302852	0.043988	0.005685	1.06E-14				
rs6795209	3	88210464	A	G	0.191251	0.041261	0.007239	1.23E-08				

rs10866704	5	176676461	A	T	0.738677	0.037919	0.006762	2.1E-08
rs200314499	10	134479675	D	I	0.339944	0.044514	0.006484	6.92E-12
rs12598836	16	4534482	G	A	0.304153	0.037775	0.005948	2.21E-10
rs7506921	18	20201527	A	T	0.532407	0.038986	0.005742	1.17E-11
rs111404218	20	10684159	G	C	0.336878	0.043633	0.006857	2.04E-10
rs1507220	X	34102712	A	C	0.301482	0.027764	0.004988	2.67E-08
rs4403550	X	40746484	T	C	0.711795	0.030532	0.005147	3.07E-09

SNP, single nucleotide polymorphisms; CHR, chromosome; BP, physical position of SNP (base-pairs); A1, effect allele; A2, alternative allele; EAF: effect allele frequency; Beta, effect allele beta coefficient.

Supplementary Table 4. Characteristics of genetic instruments of chronic kidney disease and their effect sizes with migraine.

SNP	CHR	BP	A1	A2	EAF	Exposure			Outcome			Pleiotropic traits
						Beta	SE	P-value	Beta	SE	P-value	
rs10224002	7	151415041	A	G	0.72	-0.1083	0.0102	2.65E-26	-0.01616	0.008614	0.060724	Hemoglobin, Hematocrit, Diastolic blood pressure, Pulse pressure, Systolic blood pressure
rs1049518	15	45653367	A	G	0.38	0.0788	0.0094	5.42E-17	-0.00469	0.007793	0.547122	
rs11641045	16	20404043	A	G	0.9	-0.1061	0.0148	9.03E-13	0.007214	0.012754	0.571687	
rs11649245	16	20414772	T	C	0.36	-0.0729	0.0128	1.06E-08	-0.00508	0.011203	0.650028	
rs11761603	7	1286912	T	C	0.3	-0.0674	0.0119	1.35E-08	0.002329	0.010003	0.815901	
rs12205178	6	160648923	A	G	0.12	0.0931	0.014	3.09E-11	-0.01245	0.011581	0.282286	
rs13391258	2	73848933	T	C	0.24	-0.06	0.0108	2.74E-08	-0.0024	0.009174	0.79372	
rs1458038	4	81164723	T	C	0.31	-0.059	0.01	4.21E-09	0.009697	0.008175	0.235504	Diastolic blood pressure, Hypertension, Blood pressure, Systolic blood pressure, Pulse pressure, Mean arterial pressure, Systolic blood pressure (cigarette smoking interaction), Atrial fibrillation, Diastolic blood pressure (cigarette smoking interaction)
rs17730281	15	53907948	A	G	0.23	-0.0869	0.011	2.68E-15	-0.00772	0.008936	0.387466	
rs187355703	2	176993583	C	G	0.98	-0.1987	0.0312	1.8E-10	-0.07821	0.023578	0.000915	
rs1889937	9	71403106	A	G	0.63	-0.0624	0.01	5.15E-10	0.009074	0.008118	0.263666	
rs2484639	1	243462367	A	G	0.51	-0.0774	0.0092	2.95E-17	-0.0108	0.007609	0.155842	
rs2580350	2	121996007	A	G	0.55	0.055	0.0098	1.69E-08	0.002933	0.007725	0.70421	
rs28533208	7	151409293	T	C	0.26	-0.0621	0.0113	4.18E-08	-0.00451	0.008762	0.606524	
rs35716097	5	176806636	T	C	0.32	0.0785	0.0105	8.2E-14	0.001706	0.008485	0.840621	Phosphorus levels
rs3925584	11	30760335	T	C	0.56	0.08	0.0092	4.68E-18	0.001388	0.007662	0.856176	Magnesium levels
rs4871907	8	23786784	A	C	0.55	-0.0628	0.0097	9.91E-11	-0.00984	0.007786	0.206185	
rs62300825	4	77205319	A	G	0.2	-0.0949	0.0116	2.63E-16	-0.00667	0.009369	0.476431	
rs700221	5	39357175	A	G	0.59	-0.0719	0.0098	2.19E-13	0.000213	0.007712	0.977979	
rs77713116	11	65531109	C	G	0.65	-0.0752	0.0116	1.03E-10	0.002202	0.008737	0.801041	
rs77924615	16	20392332	A	G	0.2	-0.2237	0.0128	6.38E-69	0.001385	0.010173	0.891716	Medication use (diuretics), Systolic blood pressure, Medication use (agents acting on the renin-angiotensin system), Cardiovascular disease,

												Medication use (beta blocking agents)
rs7908590	10	952523	C	G	0.93	-0.1343	0.0188	8.99E-13	-0.01609	0.015703	0.305569	Red blood cell count
rs8026369	15	39229553	A	G	0.41	-0.0553	0.0093	2.39E-09	-0.00256	0.007752	0.741473	
rs8044650	16	20350163	T	C	0.13	0.0863	0.014	6.71E-10	0.007049	0.012715	0.579319	
rs8096658	18	77156537	C	G	0.51	-0.064	0.011	5.17E-09	0.01642	0.008594	0.056058	Heel bone mineral density
rs881858	6	43806609	A	G	0.7	0.0616	0.0101	1.19E-09	0.012578	0.008469	0.137468	
rs9474801	6	54186999	A	G	0.34	0.0522	0.0096	4.61E-08	-0.0042	0.008071	0.602506	

SNP, single nucleotide polymorphisms; CHR, chromosome; BP, physical position of SNP (base-pairs); A1, effect allele; A2, alternative allele; EAF: effect allele frequency; Beta, effect allele beta coefficient.

Supplementary Table 5. Characteristics of genetic instruments of estimated glomerular filtration rate and their effect sizes with migraine.

SNP	CHR	BP	A1	A2	EAF	Exposure			Outcome			Pleiotropic traits
						Beta	SE	P-value	Beta	SE	P-value	
rs10086569	8	87247209	T	C	0.2392	0.0028	0.0004	5.73E-12	0.000475	0.009074	0.958273	
rs10096421	8	10831868	T	G	0.4706	0.002	0.0004	1.51E-08	-0.00752	0.007656	0.326282	
rs10098664	8	11417493	T	C	0.5102	-0.0024	0.0004	5.99E-11	0.004715	0.007668	0.538625	
rs10122824	9	139109861	T	G	0.3393	-0.0024	0.0004	2.84E-10	0.0091	0.008187	0.266312	
rs10224002	7	151415041	A	G	0.7175	0.0068	0.0004	2.74E-66	-0.01616	0.008614	0.060724	Hemoglobin, Hematocrit, Diastolic blood pressure, Pulse pressure, Systolic blood pressure
rs10272546	7	66111457	A	G	0.539	-0.002	0.0003	7.89E-09	-0.01206	0.007594	0.112236	
rs1028455	14	88829975	A	T	0.3316	0.0021	0.0004	1.9E-08	-0.00653	0.00813	0.421526	
rs10430743	10	126456997	T	G	0.4282	0.0025	0.0003	2.86E-13	-0.00478	0.007718	0.535461	
rs10432479	2	152365775	T	C	0.3714	-0.0021	0.0004	4.23E-09	0.008344	0.007823	0.28615	
rs1047891	2	211540507	A	C	0.3123	-0.0065	0.0004	3.59E-64	0.041306	0.008372	8.22E-07	Mean corpuscular hemoglobin, Height, Mean platelet volume, Platelet count, Fat-free mass, HDL cholesterol levels in current drinkers, Glycine levels, Systolic blood pressure, Mean corpuscular volume, Metabolite levels (small molecules and protein measures), HDL cholesterol levels x alcohol consumption (drinkers vs non-drinkers) interaction (2df), Plasma homocysteine levels (post-methionine load test), HDL cholesterol, White blood cell count, HDL cholesterol levels x alcohol consumption (regular vs non-regular drinkers) interaction (2df), HDL cholesterol levels, Alanine transaminase levels
rs1050816	2	220358198	T	C	0.3349	0.0029	0.0004	1.74E-15	0.003157	0.008064	0.695466	
rs1051447	4	49063872	A	C	0.707	0.0021	0.0004	2.58E-08	-0.02603	0.010005	0.009303	
rs10774625	12	111910219	A	G	0.4787	-0.002	0.0003	1.76E-08	-0.01943	0.007752	0.012195	Systemic lupus erythematosus, Retinal vascular caliber, coronary artery disease (myocardial infarction, percutaneous transluminal coronary angioplasty, coronary artery bypass grafting, angina or chromic ischemic heart disease), Left ventricle diastolic internal dimension, Asthma (childhood onset), Systemic lupus erythematosus, Glycated hemoglobin levels, Asthma (age of onset), Hypothyroidism

rs10838702	11	47410888	T	G	0.3788	-0.0023	0.0004	6.31E-11	0.015824	0.007844	0.043662	
rs10846157	12	15325031	A	C	0.8072	-0.0036	0.0004	1.34E-16	0.012621	0.009527	0.185241	
rs10851543	15	53962748	A	G	0.5633	0.003	0.0003	1.34E-18	0.001302	0.007669	0.865208	
rs10851885	15	76304503	A	G	0.7551	0.005	0.0004	3.28E-34	0.012909	0.009073	0.154804	
rs10865189	2	43433257	C	G	0.4747	0.0025	0.0004	1.02E-12	-0.00298	0.007699	0.698325	
rs10964603	9	20559727	T	C	0.7822	-0.0025	0.0004	7.75E-09	-0.00998	0.009251	0.280903	
rs10994860	10	52645424	T	C	0.1919	0.0039	0.0004	2.7E-18	0.00995	0.00987	0.313393	
rs11062167	12	364739	A	G	0.5303	-0.0042	0.0003	7.08E-34	-0.00168	0.007691	0.826719	
rs11063193	12	4591100	T	C	0.8777	0.0029	0.0005	4.95E-08	-0.02254	0.011715	0.0544	
rs11071738	15	63580155	T	C	0.5307	-0.0025	0.0003	5.49E-13	-0.01423	0.007652	0.062898	
rs11071939	15	67463391	T	C	0.924	-0.0038	0.0007	4.53E-09	-0.01567	0.014348	0.274901	
rs11166440	1	100808363	A	G	0.6265	0.0021	0.0004	1.1E-08	-0.00287	0.007908	0.716546	
rs111827672	19	37649866	A	T	0.3171	0.0031	0.0004	1.02E-16	-0.00811	0.008074	0.315394	
rs1119066	1	186658212	A	C	0.1536	0.0027	0.0005	1.96E-08	0.012122	0.010749	0.259422	
rs11191686	10	105187746	A	G	0.3635	0.002	0.0004	3.72E-08	0.00131	0.007875	0.867829	
rs11211257	1	46581933	A	G	0.8974	0.0039	0.0006	2.07E-11	-0.00654	0.012668	0.605957	
rs11227260	11	65461158	T	G	0.3507	-0.0032	0.0004	4.47E-19	0.005264	0.007978	0.509418	
rs11237450	11	78023356	A	C	0.1666	0.003	0.0005	2.05E-09	0.029907	0.010454	0.004237	
rs112545201	3	185803532	T	C	0.1335	-0.0042	0.0005	8.79E-17	0.017772	0.01133	0.11673	Total cholesterol levels, High density lipoprotein cholesterol levels
rs11260709	1	16557691	T	C	0.6799	0.0024	0.0004	1.87E-10	0.009118	0.00811	0.260889	
rs11261022	1	18807953	A	C	0.3608	-0.0027	0.0004	3.67E-14	-0.00168	0.007885	0.831344	
rs112880707	22	40884662	T	C	0.1086	0.0056	0.0006	6.69E-23	0.002907	0.012615	0.81772	
rs113445505	19	38157969	T	C	0.3686	0.0038	0.0004	1.96E-26	-0.00465	0.007824	0.5521	
rs113956264	16	1997004	T	C	0.0358	0.0081	0.0012	1.65E-11	-0.10373	0.051517	0.04407	
rs1153855	15	45660758	C	G	0.6213	0.0086	0.0004	1.2E-132	0.004569	0.007798	0.557977	
rs11564722	11	2178330	T	C	0.2396	0.0038	0.0004	1.41E-18	-0.00593	0.009446	0.530112	Red blood cell count
rs11657044	17	59450105	T	C	0.173	-0.0075	0.0005	5.34E-60	-0.02402	0.010448	0.021544	
rs11694902	2	121988884	A	G	0.1393	0.0041	0.0005	2.14E-16	0.01169	0.011194	0.296299	
rs117113238	12	12209203	A	G	0.0946	0.0039	0.0006	1.06E-10	-0.00157	0.013	0.903935	
rs11784052	8	8671962	T	C	0.4636	0.0027	0.0004	9.78E-15	-0.00354	0.007627	0.642744	
rs11794652	9	133496402	A	G	0.1616	-0.0028	0.0005	9.34E-09	0.021696	0.010259	0.03446	
rs11919484	3	186432839	T	G	0.3144	-0.0023	0.0004	3.98E-10	0.004577	0.008169	0.575262	
rs11951093	5	39421736	A	G	0.4157	-0.0056	0.0004	2.05E-54	0.004516	0.007755	0.560356	
rs12024377	1	205537858	A	G	0.3697	0.002	0.0004	4.25E-08	-0.00858	0.007948	0.280489	
rs12163971	5	132226669	A	C	0.164	-0.0032	0.0005	4.33E-12	-0.01521	0.010097	0.131866	
rs12207180	6	160633107	A	T	0.119	-0.0085	0.0005	1.21E-56	-0.01235	0.011725	0.292407	
rs12361687	11	9890052	A	G	0.3608	0.0021	0.0004	5.03E-09	0.028465	0.007866	0.000298	
rs1242484	17	17351643	T	C	0.6852	-0.0025	0.0004	8.8E-11	0.002645	0.008253	0.748657	
rs12520984	5	52787358	C	G	0.3264	0.0022	0.0004	2.37E-09	-0.00181	0.008108	0.823129	
rs1268176	6	109018046	A	G	0.344	0.0027	0.0004	7.05E-14	0.006871	0.008196	0.401792	

rs12736457	1	113258293	C	G	0.8703	0.0056	0.0005	8.79E-27	0.014613	0.012084	0.226526	
rs12913015	15	39305443	T	C	0.4439	0.0028	0.0004	4.62E-15	0.00929	0.007683	0.226584	
rs12920176	16	51761084	A	C	0.5893	-0.0026	0.0004	2.35E-13	-0.00552	0.00778	0.477712	
rs12989250	2	148776438	A	G	0.3129	-0.0026	0.0004	1.23E-11	-0.00071	0.008227	0.931568	
rs13029395	2	227344207	T	C	0.1842	0.0034	0.0006	1.48E-09	0.002752	0.011215	0.806149	
rs13157326	5	34504277	A	G	0.4829	-0.0027	0.0004	2.95E-12	-0.00109	0.00762	0.886663	
rs13200335	6	41690823	A	C	0.4154	0.0024	0.0003	1.5E-11	-0.01837	0.007742	0.017708	
rs13230509	7	1286192	C	G	0.686	-0.0055	0.0004	4.04E-37	-0.00082	0.009905	0.93428	
rs1377164	18	59328934	T	C	0.2142	0.0034	0.0004	9.84E-16	0.009263	0.009185	0.313184	
rs1397764	3	141750810	A	G	0.2756	0.0047	0.0004	7.83E-34	-0.0015	0.008507	0.860378	
rs140179699	2	120936492	A	G	0.9537	0.0073	0.0011	1.16E-11	0.027699	0.021626	0.200242	
rs144100226	6	34180297	T	C	0.0386	0.006	0.0011	1.26E-08	-0.00628	0.020688	0.761607	
rs1458038	4	81164723	T	C	0.2993	0.0032	0.0004	3.6E-17	0.009697	0.008175	0.235504	Diastolic blood pressure, Hypertension, Blood pressure, Systolic blood pressure, Pulse pressure, Mean arterial pressure, Systolic blood pressure (cigarette smoking interaction), Atrial fibrillation, Diastolic blood pressure (cigarette smoking interaction)
rs1509117	20	8303120	A	T	0.3044	0.0025	0.0004	3.33E-10	-0.00812	0.008827	0.357717	
rs1543238	8	8134809	A	G	0.5267	-0.0025	0.0004	3.21E-12	0.011547	0.007617	0.129528	
rs154656	16	89708003	A	T	0.4398	-0.0031	0.0003	1.32E-18	0.003203	0.007952	0.687152	Medication use (diuretics)
rs1548945	2	217665788	T	C	0.4131	0.0037	0.0004	1.27E-24	0.029938	0.007756	0.000114	
rs1569011	14	81853291	A	G	0.4368	0.002	0.0003	1.73E-08	-0.00049	0.007648	0.949231	
rs1570521	20	62911019	T	G	0.4144	0.002	0.0004	8.54E-09	-0.00219	0.008086	0.786677	
rs1595810	2	12115479	A	G	0.1952	-0.0024	0.0004	4.4E-08	0.007932	0.009548	0.406132	
rs1635404	16	3747042	T	G	0.7041	-0.0024	0.0004	2.53E-10	-0.00388	0.008305	0.640009	
rs168505	2	54920968	T	C	0.396	-0.0027	0.0004	2.18E-14	-0.00199	0.00776	0.797922	
rs1719934	18	5585158	A	G	0.5366	0.0028	0.0003	3.73E-16	-0.01138	0.007662	0.137363	
rs17413465	1	55718708	A	C	0.1842	0.0025	0.0004	1.36E-08	-0.01259	0.009807	0.199178	
rs17462630	2	219286541	C	G	0.3381	0.0024	0.0004	4.32E-09	-0.00216	0.008072	0.788775	
rs17696736	12	112486818	A	G	0.5738	0.002	0.0004	9.72E-09	0.021645	0.007886	0.006071	High density lipoprotein cholesterol levels, Type 1 diabetes, Systolic blood pressure x alcohol consumption interaction (2df test), Parental longevity (combined parental attained age, Martingale residuals), Mean arterial pressure, Ischemic stroke, Mean arterial pressure x alcohol consumption interaction (2df test), Low density lipoprotein cholesterol levels, Diastolic blood pressure x alcohol consumption interaction (2df test), Total cholesterol levels

rs1783827	11	57409538	A	G	0.5692	-0.0021	0.0004	5.95E-09	0.000105	0.007736	0.989164	
rs1858800	16	73024276	T	C	0.3476	0.0022	0.0004	3.66E-09	0.003863	0.008073	0.632354	Hypospadias
rs187355703	2	176993583	C	G	0.974	0.0101	0.0011	9.45E-19	-0.07821	0.023578	0.000915	
rs1883991	22	43112818	A	C	0.6879	-0.0032	0.0004	2.33E-17	-0.01874	0.00823	0.022806	
rs1887252	1	82957871	C	G	0.6365	-0.0029	0.0004	7.45E-16	-0.01033	0.007952	0.194052	
rs1910738	4	52687939	T	G	0.709	0.0023	0.0004	1.15E-08	-0.02306	0.008495	0.006649	
rs1913641	8	76483239	T	G	0.4778	-0.002	0.0003	4.77E-09	-0.00635	0.007588	0.402553	
rs1994887	15	57793765	A	C	0.2785	-0.0024	0.0004	2.34E-09	0.001219	0.008427	0.884944	
rs2039424	9	71432174	A	G	0.6232	0.0048	0.0004	9.75E-41	0.005426	0.007858	0.489948	
rs2068888	10	94839642	A	G	0.4527	-0.0026	0.0003	6.31E-14	0.001223	0.007616	0.872447	Triglycerides, Low density lipoprotein cholesterol levels, Medication use (HMG CoA reductase inhibitors), Plateletcrit, High density lipoprotein cholesterol levels, Platelet count, Total cholesterol levels
rs2071047	14	54418411	A	G	0.4065	0.002	0.0003	1.1E-08	0.002156	0.007796	0.782157	Male-pattern baldness
rs2074204	22	30403996	T	C	0.2625	-0.0025	0.0004	2.18E-10	0.002731	0.008613	0.751209	
rs2156664	11	121645005	T	C	0.2673	-0.0021	0.0004	3.76E-08	0.010282	0.008733	0.239036	
rs223308	4	103812499	A	G	0.5184	-0.0027	0.0003	3E-15	0.030834	0.007609	5.13E-05	
rs2235826	20	56143169	A	T	0.8139	-0.0033	0.0005	3.94E-13	-0.00914	0.009851	0.35375	
rs2236521	20	60892116	A	G	0.55	-0.0022	0.0004	4.79E-10	0.00752	0.007971	0.345466	Pelvic organ prolapse
rs2244237	21	37818141	T	G	0.221	0.0027	0.0004	8.43E-11	-0.00262	0.009132	0.774261	
rs2252281	17	19437187	T	C	0.6084	0.0041	0.0004	8.47E-30	0.012823	0.008237	0.119514	
rs2261092	20	62353933	A	G	0.0745	-0.0045	0.0007	1.41E-09	0.024205	0.014408	0.092976	
rs2267372	22	38598234	A	G	0.4007	0.0024	0.0004	1.25E-11	-0.01798	0.007841	0.021836	
rs2301343	2	40680149	T	G	0.7443	-0.0023	0.0004	9.08E-09	0.003247	0.008729	0.709936	Lung function (FVC)
rs233438	11	2794392	A	G	0.8122	0.0043	0.0004	2.84E-22	-0.00859	0.009739	0.377921	
rs2337143	18	46482070	A	G	0.3442	-0.0021	0.0004	5.59E-09	-0.01085	0.008019	0.176021	
rs2365286	7	156258179	A	G	0.7384	-0.0033	0.0004	2.08E-17	-0.0121	0.008877	0.172822	
rs2411192	17	34882998	A	T	0.5879	-0.0024	0.0003	5.85E-12	0.001194	0.007812	0.878535	
rs2442604	8	6388533	T	C	0.5458	-0.002	0.0003	9.18E-09	0.006218	0.007629	0.415059	
rs2472297	15	75027880	T	C	0.259	0.0039	0.0004	8.21E-20	-0.01391	0.008864	0.116543	Alcohol consumption (drinks per week), Coffee consumption (cups per day), Caffeine metabolism (plasma 1,3-dimethylxanthine (theophylline) level), Plasma clozapine levels in treatment-resistant schizophrenia, Caffeine metabolism (plasma 1,7-dimethylxanthine (paraxanthine) to 1,3,7-trimethylxanthine (caffeine) ratio), Caffeine metabolism (plasma 1,3,7-trimethylxanthine (caffeine) level)
rs2490391	1	243469669	A	C	0.4644	-0.0025	0.0003	5.46E-13	0.013045	0.007627	0.08717	
rs2509851	11	118966780	A	C	0.6278	0.0021	0.0004	1.62E-09	-0.0031	0.007857	0.693115	

rs2634675	12	48740855	A	G	0.4578	0.0028	0.0004	5.3E-13	-0.00355	0.007596	0.640118	
rs267738	1	150940625	T	G	0.7864	-0.005	0.0004	1.33E-32	-0.00766	0.009223	0.406545	Rhegmatogenous retinal detachment, HDL cholesterol, Blood protein levels, Glycated hemoglobin levels
rs2792796	1	56715908	T	C	0.6086	-0.0021	0.0004	3.14E-09	0.013131	0.007803	0.092396	
rs281380	19	49214470	T	C	0.6264	-0.0022	0.0004	1.9E-09	-0.00115	0.007811	0.883484	Multiple sclerosis
rs2823139	21	16576783	A	G	0.3439	-0.0027	0.0004	1.01E-13	-0.00251	0.007986	0.75288	Cardiovascular disease, Systolic blood pressure
rs2834317	21	35356706	A	G	0.1518	-0.0031	0.0005	2.83E-10	0.038712	0.010553	0.000246	
rs28404308	9	140103272	A	T	0.6232	0.0027	0.0005	1.98E-09	-0.02093	0.011509	0.068939	
rs284859	10	104573017	T	G	0.1934	0.0027	0.0004	1.47E-09	-0.00978	0.009622	0.309457	
rs28581385	16	79942679	A	T	0.8488	-0.0033	0.0005	1.58E-11	0.000138	0.010747	0.989733	
rs28817415	4	77401452	T	C	0.4426	-0.0074	0.0003	9.7E-104	0.013195	0.007585	0.081922	
rs2954017	8	126476873	T	C	0.4644	0.0026	0.0004	3.18E-11	-0.00258	0.007597	0.734491	
rs3018667	11	68912221	A	G	0.3246	-0.0024	0.0004	1.23E-10	-0.00291	0.008187	0.722062	
rs303937	13	72372524	A	T	0.4103	0.0027	0.0004	2.9E-14	-0.00645	0.007851	0.411314	
rs3111316	19	13038415	A	G	0.5887	-0.0019	0.0004	4.89E-08	0.000286	0.00783	0.970817	
rs3134605	6	32159956	T	C	0.7974	0.0033	0.0004	2.77E-13	0.006456	0.011288	0.567364	
rs325442	7	127457228	A	G	0.3981	0.0021	0.0003	3.02E-09	-0.00885	0.007776	0.255043	
rs34468415	2	178125142	A	G	0.6436	-0.0028	0.0004	2.76E-15	-0.00603	0.00791	0.445961	
rs35004449	3	52852897	T	G	0.2724	0.0027	0.0004	2.56E-12	-0.0114	0.008648	0.187566	Eosinophil counts
rs35072105	7	65609817	A	G	0.5502	-0.0021	0.0004	1.97E-09	-0.00968	0.007858	0.218125	
rs35472707	2	169995581	T	C	0.0504	-0.0075	0.0008	9.53E-20	0.015345	0.017988	0.393646	
rs35629566	14	93072317	C	G	0.8276	0.003	0.0005	5.73E-10	-0.00992	0.010294	0.335402	Post bronchodilator FEV1/FVC ratio
rs35662455	17	56755223	C	G	0.8843	0.003	0.0005	2.88E-08	0.007583	0.012118	0.531487	
rs363092	4	3196029	A	C	0.4247	-0.0022	0.0004	5.55E-10	-0.01507	0.007668	0.049485	
rs3757387	7	128576086	T	C	0.5549	0.0029	0.0004	2.48E-16	-0.00086	0.007634	0.910204	Systemic lupus erythematosus, Mouth ulcers, Eosinophil counts, Sjogren syndrome
rs3791221	2	226933	A	G	0.6487	0.0021	0.0004	3E-09	0.005533	0.00789	0.483168	
rs3793805	10	51049027	A	G	0.5659	-0.002	0.0004	1.03E-08	-0.01077	0.007659	0.159502	
rs3795503	1	180905694	T	C	0.3274	0.0022	0.0004	1.51E-08	0.005549	0.008275	0.502518	Height, White blood cell count
rs3797537	5	78322650	A	G	0.7118	0.0021	0.0004	1.86E-08	0.006346	0.008411	0.450547	
rs3812036	5	176813404	T	C	0.263	-0.0069	0.0004	3.19E-64	0.001455	0.008837	0.869188	
rs3822939	6	133849789	A	G	0.4593	-0.0028	0.0003	3.08E-16	0.00545	0.007606	0.473687	Heel bone mineral density
rs3845534	1	163738950	A	G	0.4909	-0.0019	0.0003	3.91E-08	0.006977	0.0076	0.358621	
rs3850625	1	201016296	A	G	0.1197	0.0048	0.0006	3.57E-18	0.007461	0.01176	0.525815	Lung function (FVC)
rs3905668	3	135931586	A	G	0.7226	-0.0025	0.0004	2.81E-11	0.000201	0.008524	0.981149	
rs3925584	11	30760335	T	C	0.549	-0.0055	0.0003	3.01E-56	0.001388	0.007662	0.856176	Magnesium levels
rs396341	11	5571897	T	C	0.2644	0.003	0.0004	1.72E-14	-0.00822	0.008627	0.340853	
rs407102	1	109846278	T	C	0.6996	0.0031	0.0004	3.8E-16	0.002287	0.008489	0.787597	General cognitive ability
rs41284816	13	50655989	T	G	0.0263	-0.0079	0.0012	1.56E-10	0.010866	0.024852	0.661975	Height

rs417237	1	228532195	T	G	0.6126	0.002	0.0004	1.14E-08	-0.02955	0.007798	0.000153	
rs419291	5	131633355	T	C	0.3876	0.0021	0.0004	4.13E-09	0.007253	0.007829	0.354217	Blood metabolite levels
rs4410790	7	17284577	T	C	0.3676	-0.0023	0.0004	1.92E-10	0.003509	0.007858	0.655244	Caffeine consumption, Coffee consumption, Caffeine metabolism
rs4441471	2	16715408	A	G	0.7195	0.0022	0.0004	1.06E-08	-0.0088	0.008459	0.298222	Orofacial clefts
rs4566	8	86361082	T	G	0.612	0.002	0.0004	1.03E-08	-0.00397	0.00778	0.609902	Feeling fed-up, Pediatric autoimmune diseases, Depressed affect
rs4567937	2	18676265	A	G	0.316	-0.0032	0.0004	6.4E-18	0.000756	0.008228	0.926725	
rs4625	3	49572140	A	G	0.679	-0.0023	0.0004	4.34E-10	-0.01359	0.008127	0.094418	
rs4656220	1	170649277	T	C	0.374	0.0021	0.0004	1.6E-09	-0.01334	0.007862	0.089658	
rs4794814	17	37696852	A	G	0.7512	-0.0059	0.0004	5.03E-49	0.010541	0.008811	0.231551	
rs4808154	19	18843752	T	C	0.714	0.0026	0.0004	7.77E-09	0.011705	0.008534	0.170202	
rs4836732	9	119266695	T	C	0.5325	0.0025	0.0003	4.69E-13	0.004556	0.007609	0.549397	Osteoarthritis (hip)
rs4871905	8	23735047	C	G	0.4199	-0.0043	0.0003	1.82E-35	0.006603	0.007659	0.388621	
rs4886425	15	74124543	A	G	0.1706	-0.0027	0.0005	4.26E-09	-0.0087	0.010204	0.393697	
rs4886699	15	75692303	A	C	0.752	0.0031	0.0004	4E-15	-0.0033	0.008885	0.710691	
rs495237	5	39950266	T	G	0.2484	0.0029	0.0004	5.47E-13	-0.00136	0.008743	0.876335	
rs499600	1	46039077	T	G	0.1518	-0.0037	0.0005	8.93E-15	0.014569	0.010491	0.164889	
rs506000	15	76817788	T	C	0.9127	-0.0038	0.0006	4.17E-10	0.027094	0.01338	0.042896	
rs509345	1	150276022	A	G	0.5191	0.0024	0.0003	1.35E-12	-0.03081	0.007569	4.73E-05	
rs544169	9	33956791	A	G	0.7362	0.0024	0.0004	9.98E-10	0.002732	0.008594	0.750547	
rs55759218	7	77453357	A	G	0.2668	-0.0039	0.0004	6.09E-24	-0.00212	0.008514	0.803365	
rs55938024	5	67742038	A	G	0.1159	-0.0065	0.0006	1.37E-26	0.004753	0.012097	0.694389	
rs56140069	16	69795323	A	T	0.8219	0.0025	0.0005	2.67E-08	-0.00794	0.009993	0.427026	
rs57126710	19	37017633	T	C	0.3501	0.0025	0.0004	6.18E-12	-0.00814	0.007891	0.302308	
rs6029640	20	39970385	A	G	0.5823	-0.0021	0.0004	7.03E-09	-0.0218	0.007919	0.005933	
rs6088528	20	33156742	A	G	0.5018	-0.0033	0.0003	9.61E-22	-0.00934	0.007627	0.220486	
rs6088734	20	33745046	A	T	0.4329	0.0029	0.0003	6.56E-17	0.017344	0.00767	0.023775	
rs6127099	20	52731402	A	T	0.721	-0.0051	0.0004	1.17E-36	0.009074	0.00883	0.30411	Vitamin D levels, Serum parathyroid hormone levels
rs6135224	20	14677650	A	G	0.6888	-0.002	0.0004	4.04E-08	-0.01419	0.008178	0.082717	
rs61993680	14	100752644	A	C	0.6477	-0.0022	0.0004	1.5E-08	0.013495	0.008244	0.10164	
rs62053077	16	71643669	T	G	0.3725	-0.0025	0.0004	3.93E-10	-0.00137	0.007868	0.861375	
rs62187541	20	1340244	A	G	0.9323	-0.0037	0.0007	4.13E-08	-0.03256	0.015044	0.030487	
rs62257555	3	51593113	A	G	0.9414	0.0048	0.0009	2.6E-08	0.037836	0.024138	0.117002	
rs62257807	3	50929873	T	C	0.0602	-0.0046	0.0008	2.92E-08	-0.04695	0.019141	0.014198	
rs62432759	6	154858365	A	G	0.7783	-0.0025	0.0004	7.56E-09	0.008069	0.009389	0.390118	
rs62491533	7	129564134	T	C	0.829	-0.0027	0.0005	2.11E-09	-0.00549	0.010039	0.584631	
rs632887	12	3392351	A	G	0.5933	0.0033	0.0004	1.08E-20	0.003638	0.007866	0.643697	
rs6458868	6	52630153	T	C	0.6482	-0.0021	0.0004	3.5E-09	-0.00582	0.007986	0.466178	
rs6481598	10	29781798	C	G	0.7818	0.0023	0.0004	3.77E-08	0.022974	0.009142	0.011992	

rs6484504	11	31424823	T	C	0.2756	-0.0032	0.0004	1.17E-16	0.012943	0.008446	0.12539	Red blood cell count, Height
rs6492982	15	41399951	T	C	0.5512	-0.0032	0.0004	5.8E-19	0.004339	0.007816	0.578793	
rs6501468	17	66427696	T	C	0.2299	0.0024	0.0004	1.37E-08	0.011313	0.009315	0.224559	
rs6546869	2	73895765	A	G	0.2249	0.0061	0.0004	1.66E-48	-0.0041	0.009352	0.661058	
rs6555317	5	498235	A	G	0.69	0.0024	0.0004	6.45E-09	-0.00348	0.009832	0.723488	
rs66473811	3	64000464	T	C	0.8379	0.0031	0.0005	2.02E-10	0.027698	0.010421	0.00788	
rs6667182	1	15914545	T	C	0.3167	-0.0043	0.0004	2.22E-23	0.003764	0.008204	0.646442	
rs6722113	2	28417504	A	G	0.3353	-0.0022	0.0004	1.34E-08	0.001558	0.008205	0.849375	
rs6779368	3	185298868	A	G	0.6608	0.0033	0.0004	8.78E-16	-0.00573	0.008231	0.486743	
rs6780429	3	30750404	A	C	0.5331	-0.002	0.0003	6.7E-09	0.005218	0.007605	0.492675	
rs6833292	4	10272429	T	C	0.4362	0.002	0.0003	1.08E-08	0.009747	0.00763	0.201463	
rs688540	1	48002447	A	G	0.8703	-0.0031	0.0006	2.3E-08	0.002641	0.011657	0.820768	
rs6921580	6	7203714	C	G	0.4127	0.0027	0.0004	1.61E-14	0.003283	0.007748	0.67175	
rs6948759	7	33095688	T	C	0.2116	-0.0026	0.0004	1.01E-09	0.000752	0.009444	0.936509	
rs6971211	7	155664686	T	C	0.4092	-0.0029	0.0004	3.1E-15	0.000785	0.007877	0.92064	
rs700753	7	46753684	C	G	0.3408	0.0033	0.0004	7.5E-20	-0.01275	0.008058	0.11356	
rs7012814	8	9173358	A	G	0.4712	0.0025	0.0004	1.76E-12	-0.01258	0.007805	0.107158	Fibrinogen levels
rs7084764	10	69960430	A	G	0.4962	0.0026	0.0003	2.92E-14	-0.00112	0.007659	0.8841	
rs7095954	10	82209232	A	T	0.468	-0.0019	0.0003	2.82E-08	0.022799	0.007621	0.002785	
rs7127946	11	48250675	T	C	0.7159	0.0023	0.0004	1.76E-09	0.016973	0.008526	0.046531	
rs71606723	4	115498457	A	T	0.7608	0.0029	0.0004	9.26E-13	-0.01209	0.009033	0.180643	
rs7169629	15	85191274	C	G	0.5235	0.0019	0.0003	2.43E-08	-0.0102	0.007577	0.178349	
rs7185391	16	68323115	T	G	0.2886	-0.0026	0.0004	1.15E-11	0.002607	0.008524	0.75975	
rs7188071	16	28917644	T	C	0.3593	0.0024	0.0004	8.62E-12	-0.01496	0.007954	0.060059	
rs7203398	16	53189672	A	C	0.7284	0.0027	0.0004	2.86E-12	-0.00905	0.008558	0.290079	
rs72683923	14	50735947	T	C	0.979	-0.0076	0.0014	1.98E-08	-0.00586	0.028242	0.835721	Hair color, Systolic blood pressure
rs72817412	16	89141490	T	C	0.0526	0.0049	0.0009	1.54E-08	0.017715	0.019218	0.356634	Body mass index
rs72834794	17	38211383	A	C	0.9148	-0.0041	0.0006	1.96E-10	0.008842	0.013746	0.52007	
rs72841902	2	73372212	A	T	0.2885	0.0022	0.0004	3.61E-09	-0.00191	0.008456	0.820885	
rs72995641	2	103166325	A	G	0.2014	-0.0026	0.0004	1.39E-09	0.006576	0.009364	0.482524	
rs73116829	7	50739738	A	G	0.1137	-0.0043	0.0006	1.64E-13	0.001547	0.0124	0.900661	
rs7326821	13	96068204	A	G	0.8267	0.0026	0.0005	3.82E-08	0.006546	0.010171	0.519817	
rs736820	20	43034016	A	G	0.3682	-0.0021	0.0004	5.27E-09	-0.00626	0.007943	0.430409	
rs7514450	1	220991171	T	C	0.4253	0.0022	0.0003	1.48E-10	-0.01045	0.007666	0.172893	
rs75267082	2	188129669	A	T	0.8922	0.0034	0.0006	1.15E-09	0.010685	0.012234	0.382443	
rs7535253	1	214744893	T	C	0.2102	0.0023	0.0004	4.86E-08	0.001669	0.009312	0.857725	
rs7543734	1	94050911	C	G	0.2012	0.0031	0.0005	9.58E-11	0.019685	0.009301	0.034322	
rs7565830	2	159810691	A	G	0.7164	-0.0022	0.0004	8.77E-09	-0.02085	0.00859	0.015223	
rs7592697	2	230665303	T	C	0.6508	-0.002	0.0004	3.18E-08	0.001011	0.008053	0.90011	
rs76215063	5	68265211	T	C	0.9187	-0.0041	0.0007	2.61E-09	0.004077	0.01457	0.779646	

rs7651407	3	48443816	T	C	0.4544	0.0027	0.0004	1.56E-11	0.034144	0.011129	0.002163	
rs7667050	4	23813109	T	C	0.471	0.002	0.0003	2.98E-09	0.008634	0.00758	0.254677	
rs7687209	4	109693926	T	C	0.4155	0.0022	0.0004	1.47E-09	0.001152	0.007814	0.88272	
rs77915916	6	43287722	A	T	0.9162	0.0047	0.0006	3.49E-14	0.032215	0.013928	0.020745	Self-reported math ability
rs77924615	16	20392332	A	G	0.2017	0.0096	0.0005	1.2E-99	0.001385	0.010173	0.891716	Medication use (diuretics), Systolic blood pressure, Medication use (agents acting on the renin-angiotensin system), Cardiovascular disease, Medication use (beta blocking agents)
rs780094	2	27741237	T	C	0.3846	0.0046	0.0004	4.16E-38	0.014002	0.007832	0.073794	C-reactive protein levels, LDL cholesterol, Type 2 diabetes, Fasting blood insulin, Fasting blood glucose, Triglycerides, Homeostasis model assessment of insulin resistance, Plasma omega-3 polyunsaturated fatty acid levels (docosapentaenoic acid), Metabolic traits, Alcohol consumption, Fasting blood glucose (BMI interaction), C-reactive protein, Renal underexcretion gout, Crohn's disease, Metabolic syndrome (multivariate analysis), Urate levels in overweight individuals, Urate levels in obese individuals, Gondoic acid (20:1n-9) levels, Alcohol consumption (drinks per week), Fasting blood insulin (BMI interaction), Triglyceride levels, Red blood cell count, Hypertriglyceridemia, Low density lipoprotein cholesterol levels, Urate levels, Blood glucose levels, Total cholesterol levels, Fasting plasma glucose, Glycemic traits (multi-trait analysis), Calcium levels, Age-related disease endophenotypes, Height, Age-related diseases, mortality and associated endophenotypes, Uric acid levels ,Serum metabolite levels, Metabolic syndrome, Nonalcoholic fatty liver disease
rs7832708	8	10190040	T	C	0.492	0.0023	0.0004	2.45E-10	-0.00502	0.00761	0.509143	Well-being spectrum (multivariate analysis), Self-reported math ability, General factor of neuroticism, Systolic blood pressure x alcohol consumption (light vs heavy) interaction (2df test)
rs7838146	8	22492143	T	C	0.3617	-0.0021	0.0004	1.18E-08	-0.00843	0.007907	0.286639	
rs78444298	1	184672098	A	G	0.0186	-0.0107	0.0014	2.53E-14	0.103072	0.037182	0.005584	Height
rs78614739	1	27174180	T	C	0.1668	0.0026	0.0005	2.06E-08	0.017406	0.010472	0.096491	
rs78936994	8	120894208	T	G	0.2193	0.0024	0.0004	1.02E-08	0.004356	0.009124	0.633089	

rs78986840	1	208051123	T	C	0.9393	-0.0045	0.0007	1.37E-09	-0.00661	0.016246	0.684357	
rs7966357	12	51209838	C	G	0.6627	0.0024	0.0004	6E-11	0.001881	0.008167	0.817868	
rs7974833	12	57791833	T	C	0.7574	-0.0032	0.0004	3.97E-15	-0.00294	0.008712	0.735746	
rs79760705	5	53298716	T	G	0.1087	0.0056	0.0006	2.55E-24	-0.01692	0.012187	0.165006	
rs80282103	10	899071	A	T	0.9158	0.0081	0.0006	2.58E-37	-0.00554	0.014748	0.707013	
rs80576	22	36539804	A	G	0.1625	-0.0027	0.0005	1.01E-08	0.007505	0.010274	0.465142	
rs807624	2	15782471	T	G	0.3428	0.0034	0.0004	1.53E-20	0.001096	0.00793	0.890049	Wilms tumor
rs8096658	18	77156537	C	G	0.5142	0.0046	0.0004	1.77E-29	0.01642	0.008594	0.056058	Heel bone mineral density
rs8101667	19	33402419	T	C	0.3327	0.005	0.0004	2.19E-43	-0.00962	0.008067	0.232953	
rs816828	10	79291868	T	C	0.5131	-0.002	0.0004	2.37E-08	-0.0215	0.007585	0.004605	
rs881858	6	43806609	A	G	0.6957	-0.0056	0.0004	1.15E-49	0.012578	0.008469	0.137468	
rs9375694	6	130356608	A	G	0.7031	0.0026	0.0004	4.63E-12	0.009006	0.008267	0.275958	
rs9375818	6	131882078	A	G	0.2294	-0.0026	0.0004	3.24E-10	-0.0137	0.009237	0.137998	
rs956006	15	62808539	T	C	0.3386	0.0022	0.0004	5.72E-09	0.000688	0.00824	0.933443	Pulse pressure, Diastolic blood pressure
rs9807656	18	42346956	T	C	0.9023	-0.0034	0.0006	3.76E-09	-0.01354	0.013019	0.29823	
rs9828976	3	136536835	C	G	0.7542	-0.0024	0.0004	2.04E-09	-0.00598	0.008874	0.500605	
rs9838792	3	38546726	A	G	0.386	0.0031	0.0004	4.76E-19	-1.8E-05	0.007961	0.998217	
rs9868185	3	121657593	A	G	0.5417	0.0027	0.0003	1.5E-14	0.004311	0.00769	0.575094	
rs9887775	1	23702531	A	G	0.8137	-0.0035	0.0004	5.15E-15	0.021531	0.009743	0.02714	
rs988911	2	61607510	A	G	0.1334	-0.0029	0.0005	1.43E-08	0.011122	0.011287	0.324453	
rs9894634	17	1967501	T	C	0.6007	-0.0021	0.0003	1.69E-09	-0.0341	0.00768	9.13E-06	
rs9907229	17	58917399	T	C	0.8461	-0.0049	0.0005	6.83E-24	-0.00446	0.010752	0.678531	

SNP, single nucleotide polymorphisms; CHR, chromosome; BP, physical position of SNP (base-pairs); A1, effect allele; A2, alternative allele; EAF: effect allele frequency; Beta, effect allele beta coefficient.

Supplementary Table 6. Characteristics of genetic instruments of urinary albumin-to-creatinine ratio and their effect sizes with migraine.

SNP	CHR	BP	A1	A2	EAF	Exposure			Outcome			Pleiotropic traits
						Beta	SE	P-value	Beta	SE	P-value	
rs10023335	4	77358987	T	C	0.5932	0.01439	0.002017	9.72E-13	-0.0147	0.00771	0.056584	
rs1010553	3	52540773	T	C	0.5176	0.011218	0.001988	1.67E-08	-0.01892	0.007623	0.013095	Hematocrit
rs10207567	2	203714973	C	G	0.8151	0.019371	0.002552	3.18E-14	0.053195	0.009971	9.78E-08	Smoking initiation (ever regular vs never regular) (MTAG)
rs1047891	2	211540507	A	C	0.3148	-0.01899	0.002175	2.55E-18	0.041306	0.008372	8.22E-07	Mean corpuscular hemoglobin, Height, Mean platelet volume, Platelet count, Fat-free mass, HDL cholesterol levels in current drinkers, Glycine levels, Systolic blood pressure, Mean corpuscular volume, Metabolite levels (small molecules and protein measures), HDL cholesterol levels x alcohol consumption (drinkers vs non-drinkers) interaction (2df), Plasma homocysteine levels (post-methionine load test), HDL cholesterol, White blood cell count, HDL cholesterol levels x alcohol consumption (regular vs non-regular drinkers) interaction (2df), HDL cholesterol levels, Alanine transaminase levels
rs1057868	7	75615006	T	C	0.2846	0.012172	0.002199	3.09E-08	-0.01035	0.008316	0.213249	
rs11078597	17	1618363	T	C	0.813	-0.01599	0.002595	7.13E-10	-0.00282	0.010267	0.783313	
rs11158763	14	69253343	T	C	0.4637	-0.01349	0.001992	1.26E-11	0.018278	0.00758	0.01591	
rs112607182	3	170027407	T	C	0.0751	0.030135	0.004102	2.03E-13	-0.01491	0.017388	0.391231	
rs113139575	11	10296221	C	G	0.9366	-0.02484	0.004086	1.21E-09	0.030955	0.015813	0.050289	
rs11659764	18	53335512	A	T	0.0527	0.030078	0.004475	1.81E-11	0.030265	0.017487	0.08351	Corneal resistance factor, Body mass index, Intraocular pressure
rs11912350	22	30748027	T	C	0.7582	-0.01301	0.002332	2.44E-08	-0.02012	0.009069	0.026556	
rs12714144	2	85754578	A	T	0.8733	0.022464	0.002987	5.49E-14	-0.00073	0.011849	0.950954	
rs12790943	11	120058623	T	C	0.422	0.013654	0.002013	1.17E-11	0.013774	0.007727	0.074665	Height
rs1309546	5	64290004	T	C	0.5515	0.012364	0.001997	5.9E-10	0.001512	0.007605	0.842381	
rs13132085	4	56460085	A	G	0.2893	-0.01276	0.002197	6.28E-09	0.008716	0.00837	0.297701	
rs1337526	1	47965130	A	G	0.1983	-0.02709	0.002488	1.34E-27	0.011737	0.009516	0.2174	
rs146311723	15	63804507	T	C	0.8235	-0.01542	0.002658	6.64E-09	-0.01188	0.010074	0.238339	Atrial fibrillation
rs147215801	10	17436778	T	C	0.0151	0.058263	0.008657	1.69E-11	-0.03848	0.037549	0.3055	
rs15052	19	41813375	T	C	0.8253	0.017324	0.002729	2.17E-10	0.005088	0.010458	0.626638	

rs1544935	6	39124448	T	G	0.7836	-0.01725	0.002421	1.02E-12	0.004749	0.00911	0.602168	Myocardial infarction, Urolithiasis, Diastolic blood pressure
rs162890	5	131623658	T	C	0.3317	0.013455	0.002176	6.27E-10	0.011134	0.008231	0.176154	
rs16864515	1	171435542	A	C	0.0965	-0.01887	0.003362	1.98E-08	0.013356	0.012734	0.294216	
rs1688031	19	35556640	T	C	0.142	-0.01949	0.002893	1.64E-11	-0.01529	0.010998	0.164482	
rs17035646	1	10796547	A	G	0.3398	0.01203	0.002119	1.36E-08	0.000376	0.007998	0.962487	Systolic blood pressure x alcohol consumption (light vs heavy) interaction (2df test), Systolic blood pressure, Mean arterial pressure x alcohol consumption (light vs heavy) interaction (2df test), Diastolic blood pressure x alcohol consumption (light vs heavy) interaction (2df test), Pulse pressure x alcohol consumption interaction (2df test), Systolic blood pressure x alcohol consumption interaction (2df test), Systolic blood pressure
rs17158386	7	29805361	A	G	0.2584	0.019817	0.002336	2.17E-17	-0.00061	0.008996	0.946091	
rs2023844	7	27243238	A	G	0.9255	0.026523	0.003782	2.32E-12	0.012532	0.014075	0.37329	
rs2068888	10	94839642	A	G	0.4515	-0.01243	0.002003	5.5E-10	0.001223	0.007616	0.872447	Triglycerides, Low density lipoprotein cholesterol levels, Medication use (HMG CoA reductase inhibitors), Plateletcrit, High density lipoprotein cholesterol levels, Platelet count, Total cholesterol levels
rs2240060	6	31114900	A	G	0.2881	0.013634	0.002206	6.44E-10	-0.00789	0.008571	0.357571	
rs2433611	15	45665653	A	C	0.2594	-0.01754	0.002264	9.11E-15	-0.01064	0.008542	0.212723	
rs2470893	15	75019449	T	C	0.3259	0.023034	0.002154	1.08E-26	-0.00946	0.008223	0.250006	Platelet distribution width, Coffee consumption, Caffeine metabolism (plasma 1,3-dimethylxanthine (theophylline) level), Caffeine consumption
rs2601006	12	69979517	T	C	0.343	-0.01545	0.002093	1.56E-13	0.012055	0.007996	0.131628	
rs2793351	10	22151578	A	G	0.6854	0.012102	0.002163	2.21E-08	-0.01396	0.008161	0.08709	
rs2880119	2	111809330	A	C	0.8582	-0.01641	0.002856	9.22E-09	-0.00211	0.010933	0.847277	
rs2954021	8	126482077	A	G	0.4915	0.014846	0.001982	6.89E-14	-0.00283	0.007567	0.708217	HDL cholesterol levels x alcohol consumption (drinkers vs non-drinkers) interaction (2df), White blood cell count, LDL cholesterol levels x alcohol consumption (regular vs non-regular drinkers) interaction (2df), LDL cholesterol levels in current drinkers, Neutrophil percentage of white

												cells, HDL cholesterol levels x alcohol consumption (regular vs non-regular drinkers) interaction (2df), Triglycerides, HDL cholesterol levels in current drinkers, Triglyceride levels x alcohol consumption (drinkers vs non-drinkers) interaction (2df), Total cholesterol levels, Triglyceride levels, LDL cholesterol levels x alcohol consumption (drinkers vs non-drinkers) interaction (2df), Triglyceride levels x alcohol consumption (regular vs non-regular drinkers) interaction (2df), HDL cholesterol levels, Gamma glutamyl transferase levels, Body mass index, LDL cholesterol, Red cell distribution width, Medication use (HMG CoA reductase inhibitors), LDL cholesterol levels, High density lipoprotein cholesterol levels, Serum alkaline phosphatase levels, Triglyceride levels in current drinkers, Lymphocyte percentage of white cells, Liver enzyme levels (alkaline phosphatase), Liver enzyme levels (alanine transaminase), Low density lipoprotein cholesterol levels
rs34257409	1	155131394	T	G	0.4038	0.01609	0.00202	1.63E-15	0.010064	0.007701	0.191261	
rs35572189	17	79419025	A	G	0.3641	-0.01199	0.002164	3.05E-08	0.006412	0.009908	0.517543	Alcohol use disorder (consumption score), Alcohol consumption (drinks per week)
rs35692677	7	69902654	A	G	0.1863	-0.01635	0.002595	2.93E-10	0.001984	0.00961	0.836393	
rs3734692	6	43817791	A	T	0.6908	-0.01772	0.002181	4.49E-16	0.008205	0.008254	0.3202	
rs3784283	15	41867782	A	T	0.5955	0.015135	0.00203	8.87E-14	0.002844	0.00782	0.716082	
rs3850625	1	201016296	A	G	0.1187	0.017718	0.003121	1.36E-08	0.007461	0.01176	0.525815	Lung function (FVC)
rs4410790	7	17284577	T	C	0.3689	-0.02192	0.002061	2.03E-26	0.003509	0.007858	0.655244	Caffeine metabolism (plasma 1,7-dimethylxanthine (paraxanthine) to 1,3,7-trimethylxanthine (caffeine) ratio), Coffee consumption (cups per day), Caffeine consumption
rs45551835	10	16932384	A	G	0.0147	0.201076	0.00842	4.8E-126	-0.02775	0.034004	0.414429	
rs4641276	1	33760743	T	C	0.2455	-0.01284	0.002331	3.61E-08	-0.00431	0.008963	0.63083	
rs4665972	2	27598097	T	C	0.3953	0.017373	0.002078	6.2E-17	0.010887	0.007987	0.17283	Low density lipoprotein cholesterol levels, Urinary sodium

												to potassium ratio, Reticulocyte count, Triglyceride levels, Triglyceride levels x alcohol consumption (regular vs non-regular drinkers) interaction (2df), Triglyceride levels x alcohol consumption (drinkers vs non-drinkers) interaction (2df), LDL cholesterol levels in current drinkers, High light scatter reticulocyte count, Triglyceride levels in current drinkers, LDL cholesterol levels x alcohol consumption (drinkers vs non-drinkers) interaction (2df), Breast size, Total cholesterol levels, LDL cholesterol levels x alcohol consumption (regular vs non-regular drinkers) interaction (2df), Triglycerides, Reticulocyte fraction of red cells
rs4738817	8	61620613	A	G	0.4535	-0.01153	0.001994	7.39E-09	-0.00149	0.007704	0.846675	
rs56164452	15	75623664	A	G	0.272	0.018347	0.002262	4.94E-16	-0.01362	0.008633	0.114633	
rs6119771	20	30770375	C	G	0.4257	0.01129	0.002009	1.91E-08	0.023276	0.007696	0.002501	
rs6535594	4	149132756	A	G	0.4979	0.014369	0.001994	5.74E-13	0.003159	0.007584	0.67703	
rs67339103	10	77893686	A	G	0.2163	0.017214	0.002457	2.45E-12	0.004313	0.009238	0.64062	
rs677888	17	37461018	T	G	0.7598	-0.01432	0.002325	7.3E-10	0.01207	0.009103	0.184861	
rs6998967	8	81364205	A	G	0.1664	-0.01524	0.002687	1.4E-08	-0.00264	0.010639	0.803968	Late-onset myasthenia gravis
rs7115200	11	71752160	T	G	0.5605	-0.01225	0.002044	2.02E-09	-0.0037	0.008931	0.679081	Height
rs73065147	3	46894939	T	C	0.9306	-0.02643	0.003927	1.69E-11	-0.02359	0.014924	0.113994	
rs7597336	2	227942519	A	G	0.8726	-0.01999	0.002993	2.43E-11	-0.00462	0.011911	0.69787	
rs76027714	5	53275370	A	G	0.9238	0.023445	0.00384	1.03E-09	-0.00783	0.014434	0.587724	
rs7812843	8	23737080	A	G	0.501	-0.0117	0.001983	3.62E-09	0.004539	0.007575	0.549045	
rs78444298	1	184672098	A	G	0.0192	-0.04735	0.007507	2.84E-10	0.103072	0.037182	0.005584	Height
rs78999781	2	204290037	T	C	0.8957	0.020025	0.003272	9.36E-10	0.013057	0.012972	0.314141	
rs819636	1	200271408	T	C	0.6609	-0.01209	0.002141	1.63E-08	0.015905	0.008099	0.049577	
rs838142	19	49252151	A	G	0.7203	0.017184	0.002304	8.78E-14	0.002441	0.008578	0.775942	
rs988712	11	27563382	T	G	0.2363	-0.01317	0.00234	1.79E-08	0.020106	0.009024	0.025887	Obesity

SNP, single nucleotide polymorphisms; CHR, chromosome; BP, physical position of SNP (base-pairs); A1, effect allele; A2, alternative allele; EAF: effect allele frequency; Beta, effect allele beta coefficient.

Supplementary Table 7. Data sources, sample sizes, number of instruments and F-statistics.

Phenotypes	IVs	Sample size	R^2 (%)	F statistics	Author	Ethnicity	Journal	Year	Websites for full summary statistics
Migraine	123	Ncase=102084/ Ncontrol=771257	0.81	57.97	Hautakang as, Heidi	European	Nature Genetics	2022	Please contact Dale Nyholt, d.nyholt@qut.edu.au
CKD	27‡	Ncase=41395/ Ncontrol=439303	0.32	57.15	Wuttke, Matthias	European	Nature Genetics	2019	http://ckdgen.imbi.uni-freiburg.de
eGFR	256	Ntotal=567460	2.97	67.82	Wuttke, Matthias	European	Nature Genetics	2019	http://ckdgen.imbi.uni-freiburg.de
UACR	68	Ntotal=547361	0.63	51.03	Teumer, Alexander	European	Nature Communications	2019	http://ckdgen.imbi.uni-freiburg.de

IV: instrumental variables; CKD, chronic kidney disease; eGFR, estimated glomerular filtration rate; UACR, urinary albumin-to-creatinine ratio.

$R^2=2\times\beta^2\times MAF\times(1-MAF)/(2\times\beta^2\times MAF\times(1-MAF)+2se(\beta)^2\times N\times MAF\times(1-MAF))$, β denotes the SNP effect, MAF denotes minor allele frequency, N denotes the sample size.

$F=(N-K-1)/K)\times(R^2/(1-R^2)$, N denotes the sample size, K denotes the number of IVs.

†using TwoSampleMR R package "clump_data" function (parameters: clump-p1=5e-8, clump-p2=1e-5, clump-r2=0.1, clump-kb=500, pop="EUR")

Supplementary Table 8. Baseline Characteristics of UK Biobank participants by migraine status at the baseline.

Characteristics	All	Without migraine	With migraine
Total (N)	255,896	247,876	8,020
Sex, n (%)			
Female	140,056 (54.7)	133,732 (54.0)	6,324 (78.9)
Male	115,840 (45.3)	114,144 (46.0)	1,696 (21.1)
Age at recruitment (years), mean ± SD	54.6 ± 7.9	54.7 ± 7.9	53.6 ± 7.6
Assessment center, n (%)			
England	223,713 (87.4)	216,625 (87.4)	7,088 (88.4)
Scotland	20,714 (8.1)	20,148 (8.1)	566 (7.1)
Wales	11,469 (4.5)	11,103 (4.5)	366 (4.6)
Average total household income before tax, n (%)			
<£18,000	43,575 (19.5)	42,142 (19.4)	1,433 (20.6)
£18,000 to £30,999	53,581 (24.0)	51,970 (24.0)	1,611 (23.2)
£31,000 to £51,999	61,762 (27.6)	59,827 (27.6)	1,935 (27.8)
£52,000 to £100,000	51,144 (22.9)	49,579 (22.9)	1,565 (22.5)
>£100,000	13,626 (6.1)	13,218 (6.1)	408 (5.9)
Townsend deprivation index at recruitment, n (%)			
<-2.00 (least deprived)	133,926 (52.4)	129,653 (52.4)	4,273 (53.4)
-2.00 to 1.99 (average)	81,972 (32.1)	79,445 (32.1)	2,527 (31.6)
≥2.00 (most deprived)	39,677 (15.5)	38,468 (15.5)	1,209 (15.1)
Current tobacco smoking, n (%)			
No	224,577 (87.8)	217,306 (87.7)	7,271 (90.7)
Yes, on most or all days	23,549 (9.2)	22,959 (9.3)	590 (7.4)
Only occasionally	7,632 (3.0)	7,476 (3.0)	156 (1.9)
Alcohol intake frequency, n (%)			
Daily or almost daily	55,090 (21.5)	54,011 (21.8)	1,079 (13.5)
Three or four times a week	62,781 (24.6)	61,414 (24.8)	1,367 (17.0)
Once or twice a week	67,480 (26.4)	65,394 (26.4)	2,086 (26.0)
One to three times a month	28,405 (11.1)	27,137 (11.0)	1,268 (15.8)
Special occasions only	26,004 (10.2)	24,716 (10.0)	1,288 (16.1)
Never	15,940 (6.2)	15,009 (6.1)	931 (11.6)
IPAQ activity group, n (%)			
Low	38,606 (18.4)	37,234 (18.3)	1,372 (21.4)
Moderate	85,452 (40.8)	82,739 (40.8)	2,713 (42.4)
Hight	85,378 (40.8)	83,057 (40.9)	2,321 (36.2)
Sleep duration, n (%)			
Short (≤ 6h)	175,098 (68.8)	169,758 (68.8)	5,340 (67.1)
Normal (7-8h)	62,074 (24.4)	60,052 (24.4)	2,022 (25.4)
Long (≥ 9h)	17,388 (6.8)	16,786 (6.8)	602 (7.6)
Body mass index (BMI, kg/m^2), mean ± SD	27.1 ± 4.8	27.1 ± 4.8	26.8 ± 5.1
Type 2 diabetes mellitus, n (%)			
No	252,345 (98.6)	244,390 (98.6)	7,955 (99.2)
Yes	3,551 (1.4)	3,486 (1.4)	65 (0.8)
Hypertension, n (%)			
No	241,423 (94.3)	233,818 (94.3)	7,605 (94.8)
Yes	14,473 (5.7)	14,058 (5.7)	415 (5.2)
Dyslipidemia, n (%)			
No	249,852 (97.6)	241,988 (97.6)	7,864 (98.1)
Yes	6,044 (2.4)	5,888 (2.4)	156 (1.9)
Antimigraine use, n (%)			
No	253,333 (99.0)	247,254 (99.7)	6,079 (75.8)
Yes	2,563 (1.0)	622 (0.3)	1,941 (24.2)

SD, standard deviation;
Body mass index was calculated as weight in kilograms divided by height in meters squared.
Type 2 diabetes mellitus: ICD 10 code E11
Hypertension: ICD 9 code 401; ICD 10 code I10.
Dyslipidemia: ICD 9 code 272; ICD 10 code E78.
Antimigraine use was based on Anatomical Therapeutic Chemical classification code N02C.

Supplementary Table 9. Local heritability of migraine and estimated glomerular filtration rate, and regions that contribute significant genetic correlation as estimated by SUPERGNOVA (*P* < 0.05/2353).

Locus	local $h^2_{migraine}$	local h^2_{eGFR}	local genetic correlation	local genetic covariance	*P*
4: 103388441-104802530	2.90E-04	3.05E-04	-0.92	-2.72E-04 (3.09E-09)	1.01E-06
9: 132895131-133640777	1.35E-04	2.65E-04	-1.08	-2.04E-04 (1.84E-09)	1.89E-06
11: 77904339-79723318	1.83E-04	1.94E-04	1.39	2.62E-04 (3.13E-09)	2.84E-06
12: 110112932-113027651	1.17E-04	3.97E-04	1.05	2.26E-04 (2.50E-09)	5.97E-06

Numbers in parentheses represent variance of the genetic covariance estimated by SUPERGNOVA.
eGFR, estimated glomerular filtration rate

Supplementary Table 10. Results from cross-trait meta-analysis of migraine and chronic kidney disease (SNPs with P-CPASSOC < 5×10^{-8} and single trait P-value < 1×10^{-5} are shown).

SNP	Novel	A1	A2	Beta		P-migraine	P-kidney	P-CPASSOC	Genomic coordinates	Genes within clumping area	Established Migraine		Established CKD		Mapped genes‡
				Migraine	CKD						SNP	r^2	SNP	r^2	
**Migraine and CKD**															
**rs1047891**	No	A	C	0.041	0.055	8.22E-07	2.28E-07	6.13E-12	2: 211540507-211652153	*CPS1*			rs1047891	1	*CPS1*
**Migraine and eGFR**															
rs1566225	No	G	C	0.035	-0.002	7.90E-06	2.81E-12	2.61E-15	1: 150250636-150740358	*ADAMTSL4, ADAMTSL4-AS1, C1orf54, CIART, CTSS, ECM1, ENSA, GOLPH3L, HORMAD1, LINC00568, MCL1, MIR4257, MIR6878, MRPS21, PRPF3, RPRD2, TARS2*	rs6693567	0.48	rs509345	0.74	*RPRD2*
rs41272663	Yes	A	C	-0.043	0.002	1.34E-06	1.08E-07	2.91E-12	2: 211244863-211504198	*CPS1, CPS1-IT1, LANCL1, LOC102724820*					*LANCL1, AC007970.1*
**rs1047891**	No	A	C	0.041	-0.007	8.22E-07	3.59E-64	9.35E-65	2: 211540507-211684149	*CPS1*			rs1047891	1	*CPS1*
rs13099628	Yes	G	T	0.040	-0.002	4.86E-06	4.82E-06	4.18E-10	3: 38874391-39212907	*CSRNP1, GORASP1, MIR6822, SCN11A, TTC21A, WDR48*					*SCN11A*
rs6776700	No	A	G	0.036	0.002	2.50E-06	1.83E-11	2.95E-15	3: 48411735-48591481	*ATRIP, CCDC51, FBXW12, MIR6823, PFKFB4, PLXNB1, SHISA5, TMA7, TREX1*	rs7618883	0.99	rs7651407	0.99	*ATRIP*
rs62576116	No	A	G	0.055	0.004	3.54E-07	4.28E-12	8.05E-17	9: 119202879-119486923	*ASTN2, LOC100128505, TRIM32*	rs3891689	0.38	rs4836732	0.16	*ASTN2, RP11-67K19.3*
rs9894634	No	C	T	0.034	0.002	9.13E-06	1.69E-09	6.16E-13	17: 1958609-2178092	*HIC1, LOC101927839, SMG6*	rs9894634	1			*SMG6, HIC1*
**Migraine and UACR**															
rs1971819	No	G	C	-0.054	-0.019	6.62E-08	4.66E-14	1.03E-19	2: 203639395-204196618	*ABI2, CARF, CYP20A1, ICA1L, NBEAL1, WDR12*	rs138556413	0.16	rs10207567	1	*ICA1L, KRT8P15*
**rs1047891**	No	A	C	0.041	-0.019	8.22E-07	2.55E-18	1.06E-21	2: 211540507-211684149	*CPS1*			rs1047891	1	*CPS1*
rs4909945	No	T	C	-0.067	-0.010	5.08E-16	6.46E-06	2.28E-19	11: 10654911-10701556	*MRVI1*	rs4910165	0.98			*MRVI1*

Novel: a novel SNPs only if all following criteria were satisfied: (1) the SNP reached genome-wide significance ($P_{CPASSOC} < 5\times10^{-8}$) cross-trait; (2) the SNP did not reach genome-wide significance ($5\times10^{-8} < P_{GWAS} < 10^{-5}$) in both single-trait GWAS(s); (3) the SNP was not in LD ($r^2 < 0.05$) with any of those previously reported genome-wide significant SNPs of single traits.
Measuring linkage disequilibrium (LD) with r^2 using online LDlink. LD ($r^2 \geq 0.05$) with previous reported SNPs were presented.
CKD, chronic kidney disease; eGFR, estimated glomerular filtration rate; UACR, urinary albumin-to-creatinine ratio.

Supplementary Table 11. Previously reported genome-wide significant variants for migraine and chronic kidney disease among European ancestry.

Phenotypes	Chromosome	Position (Build 37 bp)	SNP	Nearest gene	PMID
Migraine	1	3075597	rs10218452	*PRDM16*	27322543
Migraine	1	3103312	rs12135062	*PRDM16*	27322543
Migraine	1	73899742	rs1572668	*LRRIQ3*	27322543
Migraine	1	115677183	rs2078371	*TSPAN2*	27322543
Migraine	1	115824398	rs7544256	*NGF*	27322543
Migraine	1	150510660	rs6693567	*ADAMTSL4*	27322543
Migraine	1	156450740	rs1925950	*MEF2D*	27322543
Migraine	2	203832867	rs138556413	*CARF*	27322543
Migraine	2	234756811	rs566529	*HJURP*	27322543
Migraine	2	234825093	rs10166942	*TRPM8*	27322543
Migraine	3	30480559	rs6791480	*TGFBR2*	27322543
Migraine	3	154289946	rs13078967	*GPR149*	27322543
Migraine	4	57727311	rs7684253	*SPINK2*	27322543
Migraine	6	12903957	rs9349379	*PHACTR1*	27322543
Migraine	6	32206049	rs140002913	*NOTCH4*	27322543
Migraine	6	39183470	rs10456100	*KCNK5*	27322543
Migraine	6	96853967	rs4839827	*FUT9*	27322543
Migraine	6	97042147	rs67338227	*FHL5*	27322543
Migraine	6	121846038	rs28455731	*GJA1*	27322543
Migraine	6	126049040	rs1268083	*HEY2*	27322543
Migraine	7	40406876	rs186166891	*C7orf10*	27322543
Migraine	7	111328397	rs10155855	*DOCK4*	27322543
Migraine	9	119252629	rs6478241	*ASTN2*	27322543
Migraine	10	33468124	rs2506142	*NRP1*	27322543
Migraine	10	96014622	rs10786156	*PLCE1*	27322543
Migraine	10	96019029	rs75473620	*PLCE1*	27322543
Migraine	10	100702737	rs12260159	*HPSE2*	27322543
Migraine	10	124210160	rs2223089	*ARMS2*	27322543
Migraine	11	10674044	rs4910165	*MRVI1*	27322543
Migraine	11	30547438	rs11031122	*MPPED2*	27322543
Migraine	11	102083608	rs10895275	*YAP1*	27322543
Migraine	11	133829706	rs561561	*IGSF9B*	27322543
Migraine	12	4518140	rs1024905	*FGF6*	27322543
Migraine	12	57308260	rs11172055	*SDR9C7*	27322543
Migraine	12	57527283	rs11172113	*LRP1*	27322543
Migraine	14	93595591	rs11624776	*ITPK1*	27322543
Migraine	16	75442143	rs77505915	*CFDP1*	27322543
Migraine	16	87579870	rs4081947	*ZCCHC14*	27322543

Migraine	17	5612640	rs75213074	*WSCD1*	27322543
Migraine	17	78262161	rs17857135	*RNF213*	27322543
Migraine	20	10684159	rs111404218	*JAG1*	27322543
Migraine	20	19469817	rs4814864	*SLC24A3*	27322543
Migraine	20	30628982	rs144017103	*CCM2L*	27322543
Migraine	23	40764757	rs12845494	*MED14*	27322543
Migraine	1	3075597	rs10218452	*PRDM16*	35115687
Migraine	1	7055843	rs10128028	*CAMTA1*	35115687
Migraine	1	15538493	rs12057629	*TMEM51*	35115687
Migraine	1	38366907	rs28739509	*INPP5B*	35115687
Migraine	1	39590409	rs1472662	*MACF1*	35115687
Migraine	1	60529980	rs11578492	*C1orf87*	35115687
Migraine	1	66178918	rs7511672	near *LEPR*	35115687
Migraine	1	73891226	rs56019088	near *RP4-598G3.1*	35115687
Migraine	1	92177663	rs11165300	*TGFBR3*	35115687
Migraine	1	115677183	rs2078371	near *TSPAN2*	35115687
Migraine	1	150510660	rs6693567	near *ADAMTSL4*	35115687
Migraine	1	156450873	rs2274319	*MEF2D*	35115687
Migraine	1	174601659	rs11487328	*RABGAP1L*	35115687
Migraine	1	186913055	rs6668908	*PLA2G4A*	35115687
Migraine	1	206843108	rs56140113	near *MAPKAPK2*	35115687
Migraine	1	245847455	rs72764846	*KIF26B*	35115687
Migraine	2	43649780	rs12712881	*THADA*	35115687
Migraine	2	96576609	rs4907224	*ANKRD36C*	35115687
Migraine	2	145258445	rs7564469	*ZEB2*	35115687
Migraine	2	146037564	rs895219	near *AC064865.1*	35115687
Migraine	2	156416638	rs843215	near *RNU6-546P*	35115687
Migraine	2	171234235	rs4668251	*MYO3B*	35115687
Migraine	2	176978383	rs72923449	near *HOXD10*	35115687
Migraine	2	203832867	rs138556413	*CARF*	35115687
Migraine	2	234825093	rs10166942	near *TRPM8*	35115687
Migraine	3	30472786	rs7371912	near *TGFBR2*	35115687
Migraine	3	48498456	rs7618883	*ATRIP*	35115687
Migraine	3	80302512	rs950570	near *HNRNPA3P8*	35115687
Migraine	3	86149109	rs73138150	near *CADM2*	35115687
Migraine	3	88210464	rs6795209	near *C3orf38*	35115687
Migraine	3	124607055	rs1499963	*ITGB5*	35115687
Migraine	3	154289946	rs13078967	near *GPR149*	35115687
Migraine	4	35469918	rs73805934	near *SEC63P2*	35115687
Migraine	4	57727311	rs7684253	near *SPINK2*	35115687

Migraine	5	74963277	rs42854	*ANKDD1B*	35115687
Migraine	5	81129663	rs12653216	near *SSBP2*	35115687
Migraine	5	121515195	rs11957829	near *ZNF474*	35115687
Migraine	5	122306398	rs246326	*SNX24*	35115687
Migraine	5	145752008	rs10038882	near *POU4F3*	35115687
Migraine	5	149380493	rs4705403	*TIGD6*|*HMGXB3*	35115687
Migraine	5	172645766	rs6556059	near *NKX2-5*	35115687
Migraine	5	176676461	rs10866704	*NSD1*	35115687
Migraine	6	12903957	rs9349379	*PHACTR1*	35115687
Migraine	6	22131929	rs9295536	near *PRL*	35115687
Migraine	6	30749712	rs9468830	near *IER3*	35115687
Migraine	6	31850308	rs74434374	*EHMT2*	35115687
Migraine	6	39183470	rs10456100	*KCNK5*	35115687
Migraine	6	72321017	rs34273564	near *KRT19P1*	35115687
Migraine	6	97059666	rs11153082	*FHL5*	35115687
Migraine	6	111713302	rs6568677	*REV3L*	35115687
Migraine	6	121846038	rs28455731	near *GJA1*	35115687
Migraine	6	150133954	rs9383843	near *PCMT1*	35115687
Migraine	7	40427617	rs10234636	*SUGCT*	35115687
Migraine	7	73013901	rs13235543	*MLXIPL*	35115687
Migraine	7	120481569	rs56067931	*TSPAN12*	35115687
Migraine	8	27266287	rs11782789	*PTK2B*	35115687
Migraine	8	64496159	rs4739105	near *RP11-573J24.1*	35115687
Migraine	9	14103618	rs580845	*NFIB*	35115687
Migraine	9	29372501	rs10156578	near *RP11-373A6.1*	35115687
Migraine	9	71746838	rs7034179	*TJP2*	35115687
Migraine	9	109687403	rs17723637	*ZNF462*	35115687
Migraine	9	119258583	rs3891689	*ASTN2*	35115687
Migraine	9	140743200	rs4278223	near *EHMT1*	35115687
Migraine	10	8722944	rs7916911	near *RNA5SP299*	35115687
Migraine	10	21822856	rs10828247	near *MLLT10*	35115687
Migraine	10	96039597	rs2274224	*PLCE1*	35115687
Migraine	10	100702737	rs12260159	*HPSE2*	35115687
Migraine	10	104741114	rs12260436	*CNNM2*	35115687
Migraine	10	112502662	rs869432	*RBM20*	35115687
Migraine	10	124230750	rs2672592	*HTRA1*	35115687
Migraine	10	125242283	rs11248546	near *GPR26*	35115687
Migraine	10	134479675	rs200314499	*INPP5A*	35115687
Migraine	11	3249984	rs12295710	*MRGPRE*	35115687
Migraine	11	10674044	rs4910165	*MRVI1*	35115687

Migraine	11	15126085	rs1003194	near *INSC*	35115687
Migraine	11	30547438	rs11031122	*MPPED2*	35115687
Migraine	11	46548094	rs7932866	*AMBRA1*	35115687
Migraine	11	61697078	rs12787928	near *RAB3IL1*	35115687
Migraine	11	66401373	rs566673	*RBM14-RBM4|RBM4*	35115687
Migraine	11	102070976	rs12226331	*YAP1*	35115687
Migraine	11	133745852	rs10894756	near *SPATA19*	35115687
Migraine	12	4527322	rs2160875	near *FGF6*	35115687
Migraine	12	41901277	rs1458170	*PDZRN4*	35115687
Migraine	12	57527283	rs11172113	*LRP1*	35115687
Migraine	12	90091782	rs4842676	*ATP2B1*	35115687
Migraine	12	98498223	rs10777902	near *RP11-690J15.1*	35115687
Migraine	12	124820705	rs1271309	*NCOR2*	35115687
Migraine	13	47193696	rs7335684	*LRCH1*	35115687
Migraine	13	78876537	rs7996252	*RNF219-AS1*	35115687
Migraine	13	110788441	rs2000660	near *COL4A1*	35115687
Migraine	14	27661650	rs1245463	near *RP11-384J4.2*	35115687
Migraine	14	42548912	rs1542668	near *LRFN5*	35115687
Migraine	14	58761912	rs28756401	near *ARID4A*	35115687
Migraine	14	75362552	rs55707505	*DLST*	35115687
Migraine	14	76496477	rs75002882	*IFT43*	35115687
Migraine	14	93595591	rs11624776	near *ITPK1*	35115687
Migraine	14	94844947	rs28929474	*SERPINA1*	35115687
Migraine	15	81022364	rs12708529	*ABHD17C*	35115687
Migraine	16	4534482	rs12598836	*HMOX2*	35115687
Migraine	16	75442143	rs8046696	*CFDP1*	35115687
Migraine	16	87578039	rs8052831	near *ZCCHC14*	35115687
Migraine	17	1967501	rs9894634	*SMG6*	35115687
Migraine	17	7366619	rs34914463	*ZBTB4*	35115687
Migraine	17	46632679	rs11652860	*HOXB3*	35115687
Migraine	17	47514039	rs2119930	*RP11-81K2.1*	35115687
Migraine	17	60720058	rs12452590	*MRC2*	35115687
Migraine	17	77925681	rs1285294	*TBC1D16*	35115687
Migraine	17	78256432	rs8077768	*RNF213*	35115687
Migraine	18	20201527	rs7506921	near *RBBP8*	35115687
Migraine	18	44866736	rs1019990	near *SKOR2*	35115687
Migraine	18	55192245	rs8087942	near *FECH*	35115687
Migraine	19	13339128	rs10405121	*CACNA1A*	35115687
Migraine	19	19406126	rs74182632	*SUGP1*	35115687
Migraine	19	41864509	rs1982072	*B9D2|TMEM91*	35115687

Migraine	20	10684159	rs111404218	near *JAG1*	35115687
Migraine	20	19469817	rs4814864	*SLC24A3*	35115687
Migraine	20	31168439	rs6057599	*C20orf112*	35115687
Migraine	20	45841052	rs910187	*ZMYND8*	35115687
Migraine	21	35593827	rs28451064	near *MRPS6*	35115687
Migraine	21	36935896	rs764508	*RUNX1*	35115687
Migraine	22	20142932	rs625686	near *AC006547.14*	35115687
Migraine	23	34102712	rs1507220	near *FAM47A*	35115687
Migraine	23	40746484	rs4403550	near *MED14*	35115687
eGFR	1	15914545	rs6667182	*AGMAT*	31152163
eGFR	1	16557691	rs11260709	*RSG1*	31152163
eGFR	1	18807953	rs11261022	*KLHDC7A*	31152163
eGFR	1	23702531	rs9887775	*ZNF436-AS1*	31152163
eGFR	1	27174180	rs78614739	*ZDHHC18*	31152163
eGFR	1	46039077	rs499600	*AKR1A1*	31152163
eGFR	1	46581933	rs11211257	*PIK3R3*	31152163
eGFR	1	48002447	rs688540	*FOXD2*	31152163
eGFR	1	55718708	rs17413465	*MIR4422HG*	31152163
eGFR	1	56715908	rs2792796	*LINC01767*	31152163
eGFR	1	82957871	rs1887252	*LINC01362*	31152163
eGFR	1	94050911	rs7543734	*BCAR3*	31152163
eGFR	1	100808363	rs11166440	*CDC14A*	31152163
eGFR	1	109846278	rs407102	*MYBPHL*	31152163
eGFR	1	113258293	rs12736457	*PPM1J*	31152163
eGFR	1	150276022	rs509345	*MRPS21*	31152163
eGFR	1	150940625	rs267738	*CERS2*	31152163
eGFR	1	163738950	rs3845534	*LOC100422212*	31152163
eGFR	1	170649277	rs4656220	*PRRX1*	31152163
eGFR	1	180905694	rs3795503	*KIAA1614*	31152163
eGFR	1	184672098	rs78444298	*EDEM3*	31152163
eGFR	1	186658212	rs1119066	*PACERR*	31152163
eGFR	1	201016296	rs3850625	*CACNA1S*	31152163
eGFR	1	205537858	rs12024377	*MFSD4A*	31152163
eGFR	1	208051123	rs78986840	*CD34*	31152163
eGFR	1	214744893	rs7535253	*PTPN14*	31152163
eGFR	1	220991171	rs7514450	*MARC1*	31152163
eGFR	1	228532195	rs417237	*OBSCN*	31152163
eGFR	1	243469669	rs2490391	*SDCCAG8*	31152163
eGFR	2	226933	rs3791221	*SH3YL1*	31152163
eGFR	2	12115479	rs1595810	*MIR3681HG*	31152163

eGFR	2	15782471	rs807624	*DDX1*	31152163
eGFR	2	16715408	rs4441471	*FAM49A*	31152163
eGFR	2	18676265	rs4567937	*RDH14*	31152163
eGFR	2	27741237	rs780094	*GCKR*	31152163
eGFR	2	28417504	rs6722113	*BABAM2*	31152163
eGFR	2	40680149	rs2301343	*SLC8A1*	31152163
eGFR	2	43433257	rs10865189	*ZFP36L2*	31152163
eGFR	2	54920968	rs168505	*SPTBN1*	31152163
eGFR	2	61607510	rs988911	*USP34*	31152163
eGFR	2	73372212	rs72841902	*RAB11FIP5*	31152163
eGFR	2	73895765	rs6546869	*ALMS1P1*	31152163
eGFR	2	103166325	rs72995641	*SLC9A4*	31152163
eGFR	2	120936492	rs140179699	*EPB41L5*	31152163
eGFR	2	121988884	rs11694902	*TFCP2L1*	31152163
eGFR	2	148776438	rs12989250	*ORC4*	31152163
eGFR	2	152365775	rs10432479	*NEB*	31152163
eGFR	2	159810691	rs7565830	*TANC1*	31152163
eGFR	2	169995581	rs35472707	*LRP2*	31152163
eGFR	2	176993583	rs187355703	*HOXD8*	31152163
eGFR	2	178125142	rs34468415	*NFE2L2*	31152163
eGFR	2	188129669	rs75267082	*CALCRL*	31152163
eGFR	2	211540507	rs1047891	*CPS1*	31152163
eGFR	2	217665788	rs1548945	*TNP1*	31152163
eGFR	2	219286541	rs17462630	*VIL1*	31152163
eGFR	2	220358198	rs1050816	*SPEG*	31152163
eGFR	2	227344207	rs13029395	*MIR5702*	31152163
eGFR	2	230665303	rs7592697	*TRIP12*	31152163
eGFR	3	30750404	rs6780429	*TGFBR2*	31152163
eGFR	3	38546726	rs9838792	*EXOG*	31152163
eGFR	3	48443816	rs7651407	*PLXNB1*	31152163
eGFR	3	49572140	rs4625	*DAG1*	31152163
eGFR	3	50929873	rs62257807	*DOCK3*	31152163
eGFR	3	51593113	rs62257555	*RAD54L2*	31152163
eGFR	3	52852897	rs35004449	*ITIH4*	31152163
eGFR	3	64000464	rs66473811	*PSMD6*	31152163
eGFR	3	121657593	rs9868185	*SLC15A2*	31152163
eGFR	3	135931586	rs3905668	*MSL2*	31152163
eGFR	3	136536835	rs9828976	*SLC35G2*	31152163
eGFR	3	141750810	rs1397764	*TFDP2*	31152163
eGFR	3	185298868	rs6779368	*SENP2*	31152163

eGFR	3	185803532	rs112545201	*ETV5*	31152163
eGFR	3	186432839	rs11919484	*KNG1*	31152163
eGFR	4	3196029	rs363092	*HTT*	31152163
eGFR	4	10272429	rs6833292	*WDR1*	31152163
eGFR	4	23813109	rs7667050	*PPARGC1A*	31152163
eGFR	4	49063872	rs1051447	*CWH43*	31152163
eGFR	4	52687939	rs1910738	*DCUN1D4*	31152163
eGFR	4	77401452	rs28817415	*SHROOM3*	31152163
eGFR	4	81164723	rs1458038	*FGF5*	31152163
eGFR	4	103812499	rs223308	*CISD2*	31152163
eGFR	4	109693926	rs7687209	*ETNPPL*	31152163
eGFR	4	115498457	rs71606723	*UGT8*	31152163
eGFR	5	498235	rs6555317	*SLC9A3*	31152163
eGFR	5	34504277	rs13157326	*RAI14*	31152163
eGFR	5	39421736	rs11951093	*DAB2*	31152163
eGFR	5	39950266	rs495237	*LINC00603*	31152163
eGFR	5	52787358	rs12520984	*FST*	31152163
eGFR	5	53298716	rs79760705	*ARL15*	31152163
eGFR	5	67742038	rs55938024	*PIK3R1*	31152163
eGFR	5	68265211	rs76215063	*LINC02198*	31152163
eGFR	5	78322650	rs3797537	*DMGDH*	31152163
eGFR	5	131633355	rs419291	*SLC22A4*	31152163
eGFR	5	132226669	rs12163971	*AFF4*	31152163
eGFR	5	176813404	rs3812036	*SLC34A1*	31152163
eGFR	6	7203714	rs6921580	*RREB1*	31152163
eGFR	6	32159956	rs3134605	*GPSM3*	31152163
eGFR	6	34180297	rs144100226	*HMGA1*	31152163
eGFR	6	41690823	rs13200335	*TFEB*	31152163
eGFR	6	43287722	rs77915916	*CRIP3*	31152163
eGFR	6	43806609	rs881858	*LINC01512*	31152163
eGFR	6	52630153	rs6458868	*GSTA2*	31152163
eGFR	6	109018046	rs1268176	*FOXO3*	31152163
eGFR	6	130356608	rs9375694	*L3MBTL3*	31152163
eGFR	6	131882078	rs9375818	*ARG1*	31152163
eGFR	6	133849789	rs3822939	*EYA4*	31152163
eGFR	6	154858365	rs62432759	*CNKSR3*	31152163
eGFR	6	160633107	rs12207180	*SLC22A2*	31152163
eGFR	7	1286192	rs13230509	*UNCX*	31152163
eGFR	7	17284577	rs4410790	*AHR*	31152163
eGFR	7	33095688	rs6948759	*NT5C3A*	31152163

eGFR	7	46753684	rs700753	*LOC730338*	31152163
eGFR	7	50739738	rs73116829	*GRB10*	31152163
eGFR	7	65609817	rs35072105	*CRCP*	31152163
eGFR	7	66111457	rs10272546	*KCTD7*	31152163
eGFR	7	77453357	rs55759218	*PHTF2*	31152163
eGFR	7	127457228	rs325442	*SND1*	31152163
eGFR	7	128576086	rs3757387	*IRF5*	31152163
eGFR	7	129564134	rs62491533	*UBE2H*	31152163
eGFR	7	151415041	rs10224002	*PRKAG2*	31152163
eGFR	7	155664686	rs6971211	*SHH*	31152163
eGFR	7	156258179	rs2365286	*LINC01006*	31152163
eGFR	8	6388533	rs2442604	*MCPH1*	31152163
eGFR	8	8134809	rs1543238	*FAM86B3P*	31152163
eGFR	8	8671962	rs11784052	*MFHAS1*	31152163
eGFR	8	9173358	rs7012814	*LOC157273*	31152163
eGFR	8	10190040	rs7832708	*MSRA*	31152163
eGFR	8	10831868	rs10096421	*XKR6*	31152163
eGFR	8	11417493	rs10098664	*BLK*	31152163
eGFR	8	22492143	rs7838146	*BIN3*	31152163
eGFR	8	23735047	rs4871905	*STC1*	31152163
eGFR	8	76483239	rs1913641	*HNF4G*	31152163
eGFR	8	86361082	rs4566	*CA3*	31152163
eGFR	8	87247209	rs10086569	*SLC7A13*	31152163
eGFR	8	120894208	rs78936994	*DEPTOR*	31152163
eGFR	8	126476873	rs2954017	*TRIB1*	31152163
eGFR	9	20559727	rs10964603	*MLLT3*	31152163
eGFR	9	33956791	rs544169	*UBAP2*	31152163
eGFR	9	71432174	rs2039424	*PIP5K1B*	31152163
eGFR	9	119266695	rs4836732	*ASTN2*	31152163
eGFR	9	133496402	rs11794652	*FUBP3*	31152163
eGFR	9	139109861	rs10122824	*QSOX2*	31152163
eGFR	9	140103272	rs28404308	*NDOR1*	31152163
eGFR	10	899071	rs80282103	*LARP4B*	31152163
eGFR	10	29781798	rs6481598	*SVIL*	31152163
eGFR	10	51049027	rs3793805	*PARG*	31152163
eGFR	10	52645424	rs10994860	*A1CF*	31152163
eGFR	10	69960430	rs7084764	*MYPN*	31152163
eGFR	10	79291868	rs816828	*KCNMA1*	31152163
eGFR	10	82209232	rs7095954	*TSPAN14*	31152163
eGFR	10	94839642	rs2068888	*CYP26A1*	31152163

eGFR	10	104573017	rs284859	*WBP1L*	31152163
eGFR	10	105187746	rs11191686	*PDCD11*	31152163
eGFR	10	126456997	rs10430743	*EEF1AKMT2*	31152163
eGFR	11	2178330	rs11564722	*INS-IGF2*	31152163
eGFR	11	2794392	rs233438	*KCNQ1*	31152163
eGFR	11	5571897	rs396341	*OR52H1*	31152163
eGFR	11	9890052	rs12361687	*SBF2*	31152163
eGFR	11	30760335	rs3925584	*DCDC1*	31152163
eGFR	11	31424823	rs6484504	*DNAJC24*	31152163
eGFR	11	47410888	rs10838702	*SPI1*	31152163
eGFR	11	48250675	rs7127946	*OR4B1*	31152163
eGFR	11	57409538	rs1783827	*MIR130A*	31152163
eGFR	11	65461158	rs11227260	*KAT5*	31152163
eGFR	11	68912221	rs3018667	*LOC338694*	31152163
eGFR	11	78023356	rs11237450	*GAB2*	31152163
eGFR	11	118966780	rs2509851	*DPAGT1*	31152163
eGFR	11	121645005	rs2156664	*SORL1*	31152163
eGFR	12	364739	rs11062167	*SLC6A13*	31152163
eGFR	12	3392351	rs632887	*TSPAN9*	31152163
eGFR	12	4591100	rs11063193	*C12orf4*	31152163
eGFR	12	12209203	rs117113238	*BCL2L14*	31152163
eGFR	12	15325031	rs10846157	*RERG*	31152163
eGFR	12	48740855	rs2634675	*ZNF641*	31152163
eGFR	12	51209838	rs7966357	*ATF1*	31152163
eGFR	12	57791833	rs7974833	*R3HDM2*	31152163
eGFR	12	111910219	rs10774625	*ATXN2*	31152163
eGFR	12	112486818	rs17696736	*NAA25*	31152163
eGFR	13	50655989	rs41284816	*DLEU2*	31152163
eGFR	13	72372524	rs303937	*DACH1*	31152163
eGFR	13	96068204	rs7326821	*CLDN10*	31152163
eGFR	14	50735947	rs72683923	*L2HGDH*	31152163
eGFR	14	54418411	rs2071047	*BMP4*	31152163
eGFR	14	81853291	rs1569011	*STON2*	31152163
eGFR	14	88829975	rs1028455	*SPATA7*	31152163
eGFR	14	93072317	rs35629566	*RIN3*	31152163
eGFR	14	100752644	rs61993680	*SLC25A29*	31152163
eGFR	15	39305443	rs12913015	*C15orf54*	31152163
eGFR	15	41399951	rs6492982	*INO80*	31152163
eGFR	15	45660758	rs1153855	*GATM*	31152163
eGFR	15	53962748	rs10851543	*WDR72*	31152163

eGFR	15	57793765	rs1994887	*CGNL1*	31152163
eGFR	15	62808539	rs956006	*MGC15885*	31152163
eGFR	15	63580155	rs11071738	*APH1B*	31152163
eGFR	15	67463391	rs11071939	*SMAD3*	31152163
eGFR	15	74124543	rs4886425	*TBC1D21*	31152163
eGFR	15	75027880	rs2472297	*CYP1A1*	31152163
eGFR	15	75692303	rs4886699	*SIN3A*	31152163
eGFR	15	76304503	rs10851885	*NRG4*	31152163
eGFR	15	76817788	rs506000	*SCAPER*	31152163
eGFR	15	85191274	rs7169629	*WDR73*	31152163
eGFR	16	1997004	rs113956264	*RPL3L*	31152163
eGFR	16	3747042	rs1635404	*TRAP1*	31152163
eGFR	16	20392332	rs77924615	*PDILT*	31152163
eGFR	16	28917644	rs7188071	*RABEP2*	31152163
eGFR	16	51761084	rs12920176	*LINC01571*	31152163
eGFR	16	53189672	rs7203398	*CHD9*	31152163
eGFR	16	68323115	rs7185391	*SLC7A6*	31152163
eGFR	16	69795323	rs56140069	*WWP2*	31152163
eGFR	16	71643669	rs62053077	*MARVELD3*	31152163
eGFR	16	73024276	rs1858800	*ZFHX3*	31152163
eGFR	16	79942679	rs28581385	*LINC01229*	31152163
eGFR	16	89141490	rs72817412	*ACSF3*	31152163
eGFR	16	89708003	rs154656	*CHMP1A*	31152163
eGFR	17	1967501	rs9894634	*SMG6*	31152163
eGFR	17	17351643	rs1242484	*MED9*	31152163
eGFR	17	19437187	rs2252281	*SLC47A1*	31152163
eGFR	17	34882998	rs2411192	*MYO19*	31152163
eGFR	17	37696852	rs4794814	*CDK12*	31152163
eGFR	17	38211383	rs72834794	*MED24*	31152163
eGFR	17	56755223	rs35662455	*TEX14*	31152163
eGFR	17	58917399	rs9907229	*BCAS3*	31152163
eGFR	17	59450105	rs11657044	*BCAS3*	31152163
eGFR	17	66427696	rs6501468	*PRKAR1A*	31152163
eGFR	18	5585158	rs1719934	*EPB41L3*	31152163
eGFR	18	42346956	rs9807656	*SETBP1*	31152163
eGFR	18	46482070	rs2337143	*SMAD7*	31152163
eGFR	18	59328934	rs1377164	*LINC01544*	31152163
eGFR	18	77156537	rs8096658	*NFATC1*	31152163
eGFR	19	13038415	rs3111316	*FARSA*	31152163
eGFR	19	18843752	rs4808154	*CRTC1*	31152163

eGFR	19	33402419	rs8101667	*CEP89*	31152163
eGFR	19	37017633	rs57126710	*ZNF260*	31152163
eGFR	19	37649866	rs111827672	*ZNF585A*	31152163
eGFR	19	38157969	rs113445505	*ZNF781*	31152163
eGFR	19	49214470	rs281380	*MAMSTR*	31152163
eGFR	20	1340244	rs62187541	*FKBP1A-SDCBP2*	31152163
eGFR	20	8303120	rs1509117	*PLCB1*	31152163
eGFR	20	14677650	rs6135224	*MACROD2*	31152163
eGFR	20	33156742	rs6088528	*PIGU*	31152163
eGFR	20	33745046	rs6088734	*EDEM2*	31152163
eGFR	20	39970385	rs6029640	*LPIN3*	31152163
eGFR	20	43034016	rs736820	*HNF4A*	31152163
eGFR	20	52731402	rs6127099	*CYP24A1*	31152163
eGFR	20	56143169	rs2235826	*PCK1*	31152163
eGFR	20	60892116	rs2236521	*LAMA5*	31152163
eGFR	20	62353933	rs2261092	*ZGPAT*	31152163
eGFR	20	62911019	rs1570521	*PCMTD2*	31152163
eGFR	21	16576783	rs2823139	*NRIP1*	31152163
eGFR	21	35356706	rs2834317	*LOC101928126*	31152163
eGFR	21	37818141	rs2244237	*CLDN14*	31152163
eGFR	22	30403996	rs2074204	*MTMR3*	31152163
eGFR	22	36539804	rs80576	*APOL3*	31152163
eGFR	22	38598234	rs2267372	*MAFF*	31152163
eGFR	22	40884662	rs112880707	*MKL1*	31152163
eGFR	22	43112818	rs1883991	*A4GALT*	31152163
CKD*	1	243469669	rs2490391	*SDCCAG8*	31152163
CKD*	2	113967075	rs11123169	*PSD4*	31152163
CKD*	2	176993583	rs187355703	*HOXD8*	31152163
CKD*	2	211540507	rs1047891	*CPS1*	31152163
CKD*	4	77401452	rs28817415	*SHROOM3*	31152163
CKD*	4	81182554	rs12509595	*FGF5*	31152163
CKD*	5	39378115	rs1362800	*DAB2*	31152163
CKD*	5	176813404	rs3812036	*SLC34A1*	31152163
CKD*	6	43806609	rs881858	*LINC01512*	31152163
CKD*	6	160633107	rs12207180	*SLC22A2*	31152163
CKD*	7	1286567	rs62435145	*UNCX*	31152163
CKD*	7	151415536	rs10254101	*PRKAG2*	31152163
CKD*	7	156252939	rs868822	*LINC01006*	31152163
CKD*	10	899071	rs80282103	*LARP4B*	31152163
CKD*	11	30749090	rs963837	*DCDC1*	31152163

CKD*	11	65552154	rs948493	*MIR1234*	31152163
CKD*	15	45683795	rs1145077	*GATM*	31152163
CKD*	15	53950578	rs690428	*WDR72*	31152163
CKD*	16	20392332	rs77924615	*PDILT*	31152163
CKD*	17	34882998	rs2411192	*MYO19*	31152163
CKD*	18	24393213	rs16942751	*AQP4*	31152163
CKD*	18	77156537	rs8096658	*NFATC1*	31152163
CKD*	21	16576783	rs2823139	*NRIP1*	31152163
UACR	1	10796547	rs17035646	*CASZ1*	31511532
UACR	1	33760743	rs4641276	*ZNF362*	31511532
UACR	1	47965130	rs1337526	*FOXD2*	31511532
UACR	1	155131394	rs34257409	*KRTCAP2*	31511532
UACR	1	171435542	rs16864515	*PRRC2C*	31511532
UACR	1	184672098	rs78444298	*EDEM3*	31511532
UACR	1	200271408	rs819636	*LINC00862*	31511532
UACR	1	201016296	rs3850625	*CACNA1S*	31511532
UACR	2	27598097	rs4665972	*SNX17*	31511532
UACR	2	85754578	rs12714144	*PARTICL*	31511532
UACR	2	111809330	rs2880119	*ACOXL*	31511532
UACR	2	203714973	rs10207567	*ICA1L*	31511532
UACR	2	204290037	rs78999781	*ABI2*	31511532
UACR	2	211540507	rs1047891	*CPS1*	31511532
UACR	2	227942519	rs7597336	*COL4A4*	31511532
UACR	3	46894939	rs73065147	*MYL3*	31511532
UACR	3	52540773	rs1010553	*STAB1*	31511532
UACR	3	170027407	rs112607182	*PRKCI*	31511532
UACR	4	56460085	rs13132085	*NMU*	31511532
UACR	4	77358987	rs10023335	*SHROOM3*	31511532
UACR	4	149132756	rs6535594	*NR3C2*	31511532
UACR	5	53275370	rs76027714	*ARL15*	31511532
UACR	5	64290004	rs1309546	*CWC27*	31511532
UACR	5	131623658	rs162890	*SLC22A4*	31511532
UACR	6	31114900	rs2240060	*CCHCR1*	31511532
UACR	6	39124448	rs1544935	*KCNK5*	31511532
UACR	6	43817791	rs3734692	*LINC01512*	31511532
UACR	7	17284577	rs4410790	*AHR*	31511532
UACR	7	27243238	rs2023844	*HOTTIP*	31511532
UACR	7	29805361	rs17158386	*WIPF3*	31511532
UACR	7	69902654	rs35692677	*AUTS2*	31511532
UACR	7	75615006	rs1057868	*POR*	31511532

UACR	8	23737080	rs7812843	*STC1*	31511532
UACR	8	61620613	rs4738817	*CHD7*	31511532
UACR	8	81364205	rs6998967	*ZBTB10*	31511532
UACR	8	126482077	rs2954021	*TRIB1*	31511532
UACR	10	16932384	rs45551835	*CUBN*	31511532
UACR	10	17436778	rs147215801	*ST8SIA6*	31511532
UACR	10	22151578	rs2793351	*DNAJC1*	31511532
UACR	10	77893686	rs67339103	*LRMDA*	31511532
UACR	10	94839642	rs2068888	*CYP26A1*	31511532
UACR	11	10296221	rs113139575	*SBF2*	31511532
UACR	11	27563382	rs988712	*BDNF-AS*	31511532
UACR	11	71752160	rs7115200	*NUMA1*	31511532
UACR	11	120058623	rs12790943	*OAF*	31511532
UACR	12	69979517	rs2601006	*CCT2*	31511532
UACR	14	69253343	rs11158763	*ZFP36L1*	31511532
UACR	15	41867782	rs3784283	*TYRO3*	31511532
UACR	15	45665653	rs2433611	*GATM*	31511532
UACR	15	63804507	rs146311723	*USP3*	31511532
UACR	15	75019449	rs2470893	*CYP1A1*	31511532
UACR	15	75623664	rs56164452	*COMMD4*	31511532
UACR	17	1618363	rs11078597	*MIR22HG*	31511532
UACR	17	37461018	rs677888	*FBXL20*	31511532
UACR	17	79419025	rs35572189	*BAHCC1*	31511532
UACR	18	53335512	rs11659764	*TCF4*	31511532
UACR	19	35556640	rs1688031	*HPN*	31511532
UACR	19	41813375	rs15052	*HNRNPUL1*	31511532
UACR	19	49252151	rs838142	*FUT1*	31511532
UACR	20	30770375	rs6119771	*TSPY26P*	31511532
UACR	22	30748027	rs11912350	*SF3A1*	31511532

*Trans-ancestry genome-wide significanct loci.

CKD, chronic kidney disease; eGFR, estimated glomerular filtration rate; UACR, urinary albumin-to-creatinine ratio.

Supplementary Table 12. Detailed annotation of genome-wide significant SNPs identified by cross-trait meta-analysis.

SNP	Location	Consequence	IMPACT	SYMBOL	Feature_type	BIOTYPE
**Migraine and CKD**						
rs1047891	2:211540507-211540507	missense_variant	MODERATE	*CPS1*	Transcript	protein_coding
rs1047891	2:211540507-211540507	missense_variant	MODERATE	*CPS1*	Transcript	protein_coding
rs1047891	2:211540507-211540507	missense_variant	MODERATE	*CPS1*	Transcript	protein_coding
rs1047891	2:211540507-211540507	non_coding_transcript_exon_variant	MODIFIER	*CPS1*	Transcript	retained_intron
**Migraine and eGFR**						
rs1566225	1:150415990-150415990	downstream_gene_variant	MODIFIER	*RPRD2*	Transcript	protein_coding
rs1566225	1:150415990-150415990	intron_variant	MODIFIER	*RPRD2*	Transcript	protein_coding
rs1566225	1:150415990-150415990	intron_variant	MODIFIER	*RPRD2*	Transcript	protein_coding
rs1566225	1:150415990-150415990	intron_variant,non_coding_transcript_variant	MODIFIER	*RPRD2*	Transcript	processed_transcript
rs1566225	1:150415990-150415990	intron_variant	MODIFIER	*RPRD2*	Transcript	protein_coding
rs9894634	17:1967501-1967501	intron_variant	MODIFIER	*SMG6*	Transcript	protein_coding
rs9894634	17:1967501-1967501	intron_variant	MODIFIER	*SMG6*	Transcript	protein_coding
rs9894634	17:1967501-1967501	intron_variant	MODIFIER	*SMG6*	Transcript	protein_coding
rs9894634	17:1967501-1967501	downstream_gene_variant	MODIFIER	*HIC1*	Transcript	protein_coding
rs9894634	17:1967501-1967501	downstream_gene_variant	MODIFIER	*HIC1*	Transcript	protein_coding
rs9894634	17:1967501-1967501	downstream_gene_variant	MODIFIER	*HIC1*	Transcript	protein_coding
rs9894634	17:1967501-1967501	intron_variant	MODIFIER	*SMG6*	Transcript	protein_coding
rs9894634	17:1967501-1967501	intron_variant	MODIFIER	*SMG6*	Transcript	protein_coding
rs9894634	17:1967501-1967501	intron_variant	MODIFIER	*SMG6*	Transcript	protein_coding
rs9894634	17:1967501-1967501	downstream_gene_variant	MODIFIER	*HIC1*	Transcript	protein_coding
rs9894634	17:1967501-1967501	downstream_gene_variant	MODIFIER	*HIC1*	Transcript	protein_coding
rs9894634	17:1967501-1967501	downstream_gene_variant	MODIFIER	*HIC1*	Transcript	protein_coding
rs9894634	17:1967501-1967501	intron_variant	MODIFIER	*SMG6*	Transcript	protein_coding
rs9894634	17:1967501-1967501	intron_variant	MODIFIER	*SMG6*	Transcript	protein_coding
rs9894634	17:1967501-1967501	intron_variant	MODIFIER	*SMG6*	Transcript	protein_coding
rs9894634	17:1967501-1967501	intron_variant	MODIFIER	*SMG6*	Transcript	protein_coding
rs9894634	17:1967501-1967501	intron_variant	MODIFIER	*SMG6*	Transcript	protein_coding
rs9894634	17:1967501-1967501	intron_variant	MODIFIER	*SMG6*	Transcript	protein_coding
rs9894634	17:1967501-1967501	intron_variant,non_coding_transcript_variant	MODIFIER	*SMG6*	Transcript	processed_transcript
rs9894634	17:1967501-1967501	intron_variant,non_coding_transcript_variant	MODIFIER	*SMG6*	Transcript	processed_transcript
rs9894634	17:1967501-1967501	intron_variant,non_coding_transcript_variant	MODIFIER	*SMG6*	Transcript	processed_transcript
rs9894634	17:1967501-1967501	intron_variant,non_coding_transcript_variant	MODIFIER	*SMG6*	Transcript	processed_transcript
rs9894634	17:1967501-1967501	intron_variant,non_coding_transcript_variant	MODIFIER	*SMG6*	Transcript	processed_transcript
rs9894634	17:1967501-1967501	intron_variant,non_coding_transcript_variant	MODIFIER	*SMG6*	Transcript	processed_transcript
rs9894634	17:1967501-1967501	intron_variant,non_coding_transcript_variant	MODIFIER	*SMG6*	Transcript	processed_transcript
rs9894634	17:1967501-1967501	intron_variant,non_coding_transcript_variant	MODIFIER	*SMG6*	Transcript	processed_transcript
rs9894634	17:1967501-1967501	intron_variant,non_coding_transcript_variant	MODIFIER	*SMG6*	Transcript	processed_transcript
rs9894634	17:1967501-1967501	intron_variant,non_coding_transcript_variant	MODIFIER	*SMG6*	Transcript	processed_transcript
rs9894634	17:1967501-1967501	intron_variant,non_coding_transcript_variant	MODIFIER	*SMG6*	Transcript	processed_transcript
rs9894634	17:1967501-1967501	intron_variant,non_coding_transcript_variant	MODIFIER	*SMG6*	Transcript	processed_transcript
rs9894634	17:1967501-1967501	upstream_gene_variant	MODIFIER	*SMG6*	Transcript	retained_intron
rs9894634	17:1967501-1967501	upstream_gene_variant	MODIFIER	*SMG6*	Transcript	retained_intron
rs9894634	17:1967501-1967501	upstream_gene_variant	MODIFIER	*SMG6*	Transcript	retained_intron
rs9894634	17:1967501-1967501	downstream_gene_variant	MODIFIER	*SMG6*	Transcript	protein_coding

rs9894634	17:1967501-1967501	downstream_gene_variant	MODIFIER	*SMG6*	Transcript	protein_coding
rs9894634	17:1967501-1967501	downstream_gene_variant	MODIFIER	*SMG6*	Transcript	protein_coding
rs9894634	17:1967501-1967501	intron_variant,non_coding_transcript_variant	MODIFIER	*SMG6*	Transcript	processed_transcript
rs9894634	17:1967501-1967501	intron_variant,non_coding_transcript_variant	MODIFIER	*SMG6*	Transcript	processed_transcript
rs9894634	17:1967501-1967501	intron_variant,non_coding_transcript_variant	MODIFIER	*SMG6*	Transcript	processed_transcript
rs41272663	2:211302627-211302627	intron_variant	MODIFIER	*LANCL1*	Transcript	protein_coding
rs41272663	2:211302627-211302627	intron_variant	MODIFIER	*LANCL1*	Transcript	protein_coding
rs41272663	2:211302627-211302627	intron_variant,non_coding_transcript_variant	MODIFIER	*AC007970.1*	Transcript	antisense
rs41272663	2:211302627-211302627	intron_variant	MODIFIER	*LANCL1*	Transcript	protein_coding
rs41272663	2:211302627-211302627	intron_variant,non_coding_transcript_variant	MODIFIER	*AC007970.1*	Transcript	antisense
rs41272663	2:211302627-211302627	intron_variant	MODIFIER	*LANCL1*	Transcript	protein_coding
rs41272663	2:211302627-211302627	intron_variant	MODIFIER	*LANCL1*	Transcript	protein_coding
rs41272663	2:211302627-211302627	downstream_gene_variant	MODIFIER	*LANCL1*	Transcript	protein_coding
rs41272663	2:211302627-211302627	intron_variant	MODIFIER	*LANCL1*	Transcript	protein_coding
rs1047891	2:211540507-211540507	missense_variant	MODERATE	*CPS1*	Transcript	protein_coding
rs1047891	2:211540507-211540507	missense_variant	MODERATE	*CPS1*	Transcript	protein_coding
rs1047891	2:211540507-211540507	missense_variant	MODERATE	*CPS1*	Transcript	protein_coding
rs1047891	2:211540507-211540507	non_coding_transcript_exon_variant	MODIFIER	*CPS1*	Transcript	retained_intron
rs13099628	3:38927854-38927854	intron_variant	MODIFIER	*SCN11A*	Transcript	protein_coding
rs13099628	3:38927854-38927854	intron_variant	MODIFIER	*SCN11A*	Transcript	protein_coding
rs13099628	3:38927854-38927854	intron_variant	MODIFIER	*SCN11A*	Transcript	protein_coding
rs13099628	3:38927854-38927854	intron_variant	MODIFIER	*SCN11A*	Transcript	protein_coding
rs6776700	3:48496758-48496758	intron_variant	MODIFIER	*ATRIP*	Transcript	protein_coding
rs6776700	3:48496758-48496758	intron_variant	MODIFIER	*ATRIP*	Transcript	protein_coding
rs6776700	3:48496758-48496758	intron_variant	MODIFIER	*ATRIP*	Transcript	protein_coding
rs6776700	3:48496758-48496758	intron_variant	MODIFIER	*ATRIP*	Transcript	protein_coding
rs6776700	3:48496758-48496758	intron_variant	MODIFIER	*ATRIP*	Transcript	protein_coding
rs6776700	3:48496758-48496758	intron_variant	MODIFIER	*ATRIP*	Transcript	protein_coding
rs6776700	3:48496758-48496758	intron_variant	MODIFIER	*ATRIP*	Transcript	protein_coding
rs6776700	3:48496758-48496758	intron_variant	MODIFIER	*ATRIP*	Transcript	protein_coding
rs6776700	3:48496758-48496758	intron_variant	MODIFIER	*ATRIP*	Transcript	protein_coding
rs6776700	3:48496758-48496758	intron_variant	MODIFIER	*ATRIP*	Transcript	protein_coding
rs6776700	3:48496758-48496758	intron_variant,NMD_transcript_variant	MODIFIER	*ATRIP*	Transcript	nonsense_mediated_decay
rs6776700	3:48496758-48496758	intron_variant,NMD_transcript_variant	MODIFIER	*ATRIP*	Transcript	nonsense_mediated_decay
rs6776700	3:48496758-48496758	intron_variant,NMD_transcript_variant	MODIFIER	*ATRIP*	Transcript	nonsense_mediated_decay
rs6776700	3:48496758-48496758	intron_variant,NMD_transcript_variant	MODIFIER	*ATRIP*	Transcript	nonsense_mediated_decay
rs6776700	3:48496758-48496758	intron_variant,non_coding_transcript_variant	MODIFIER	*ATRIP*	Transcript	retained_intron
rs6776700	3:48496758-48496758	intron_variant,non_coding_transcript_variant	MODIFIER	*ATRIP*	Transcript	retained_intron
rs62576116	9:119342218-119342218	intron_variant	MODIFIER	*ASTN2*	Transcript	protein_coding
rs62576116	9:119342218-119342218	intron_variant	MODIFIER	*ASTN2*	Transcript	protein_coding
rs62576116	9:119342218-119342218	intron_variant	MODIFIER	*ASTN2*	Transcript	protein_coding
rs62576116	9:119342218-119342218	intron_variant	MODIFIER	*ASTN2*	Transcript	protein_coding
rs62576116	9:119342218-119342218	intron_variant	MODIFIER	*ASTN2*	Transcript	protein_coding
rs62576116	9:119342218-119342218	intron_variant	MODIFIER	*ASTN2*	Transcript	protein_coding
rs62576116	9:119342218-119342218	intron_variant	MODIFIER	*ASTN2*	Transcript	protein_coding
rs62576116	9:119342218-119342218	intron_variant,non_coding_transcript_variant	MODIFIER	*RP11-67K19.3*	Transcript	antisense
rs1566225	HG1287_PATCH:151129929-151129929	intron_variant	MODIFIER	*RPRD2*	Transcript	protein_coding
rs1566225	HG1287_PATCH:151129929-151129929	intron_variant	MODIFIER	*RPRD2*	Transcript	protein_coding

rs1566225	HG1287_PATCH:151129929-151129929	intron_variant,non_coding_transcript_variant	MODIFIER	*RPRD2*	Transcript	processed_transcript
rs1566225	HG1287_PATCH:151129929-151129929	downstream_gene_variant	MODIFIER	*RPRD2*	Transcript	protein_coding
rs1566225	HG1287_PATCH:151129929-151129929	intron_variant	MODIFIER	*RPRD2*	Transcript	protein_coding
**Migraine and UACR**						
rs4909945	11:10673739-10673739	missense_variant	MODERATE	*MRVI1*	Transcript	protein_coding
rs4909945	11:10673739-10673739	missense_variant	MODERATE	*MRVI1*	Transcript	protein_coding
rs4909945	11:10673739-10673739	splice_polypyrimidine_tract_variant,intron_variant	LOW	*MRVI1*	Transcript	protein_coding
rs4909945	11:10673739-10673739	splice_polypyrimidine_tract_variant,intron_variant	LOW	*MRVI1*	Transcript	protein_coding
rs4909945	11:10673739-10673739	upstream_gene_variant	MODIFIER	*MRVI1*	Transcript	protein_coding
rs4909945	11:10673739-10673739	upstream_gene_variant	MODIFIER	*MRVI1*	Transcript	protein_coding
rs4909945	11:10673739-10673739	missense_variant	MODERATE	*MRVI1*	Transcript	protein_coding
rs4909945	11:10673739-10673739	missense_variant	MODERATE	*MRVI1*	Transcript	protein_coding
rs4909945	11:10673739-10673739	splice_polypyrimidine_tract_variant,intron_variant,NMD_transcript_variant	LOW	*MRVI1*	Transcript	nonsense_mediated_decay
rs4909945	11:10673739-10673739	splice_polypyrimidine_tract_variant,intron_variant,NMD_transcript_variant	LOW	*MRVI1*	Transcript	nonsense_mediated_decay
rs4909945	11:10673739-10673739	intron_variant	MODIFIER	*MRVI1*	Transcript	protein_coding
rs4909945	11:10673739-10673739	intron_variant	MODIFIER	*MRVI1*	Transcript	protein_coding
rs4909945	11:10673739-10673739	intron_variant,NMD_transcript_variant	MODIFIER	*MRVI1*	Transcript	nonsense_mediated_decay
rs4909945	11:10673739-10673739	intron_variant,NMD_transcript_variant	MODIFIER	*MRVI1*	Transcript	nonsense_mediated_decay
rs4909945	11:10673739-10673739	intron_variant,NMD_transcript_variant	MODIFIER	*MRVI1*	Transcript	nonsense_mediated_decay
rs4909945	11:10673739-10673739	intron_variant,NMD_transcript_variant	MODIFIER	*MRVI1*	Transcript	nonsense_mediated_decay
rs4909945	11:10673739-10673739	splice_polypyrimidine_tract_variant,intron_variant	LOW	*MRVI1*	Transcript	protein_coding
rs4909945	11:10673739-10673739	splice_polypyrimidine_tract_variant,intron_variant	LOW	*MRVI1*	Transcript	protein_coding
rs4909945	11:10673739-10673739	missense_variant	MODERATE	*MRVI1*	Transcript	protein_coding
rs4909945	11:10673739-10673739	missense_variant	MODERATE	*MRVI1*	Transcript	protein_coding
rs4909945	11:10673739-10673739	splice_polypyrimidine_tract_variant,intron_variant,NMD_transcript_variant	LOW	*MRVI1*	Transcript	nonsense_mediated_decay
rs4909945	11:10673739-10673739	splice_polypyrimidine_tract_variant,intron_variant,NMD_transcript_variant	LOW	*MRVI1*	Transcript	nonsense_mediated_decay
rs4909945	11:10673739-10673739	splice_polypyrimidine_tract_variant,intron_variant,non_coding_transcript_variant	LOW	*MRVI1*	Transcript	processed_transcript
rs4909945	11:10673739-10673739	splice_polypyrimidine_tract_variant,intron_variant,non_coding_transcript_variant	LOW	*MRVI1*	Transcript	processed_transcript
rs4909945	11:10673739-10673739	splice_polypyrimidine_tract_variant,intron_variant,non_coding_transcript_variant	LOW	*MRVI1*	Transcript	processed_transcript
rs4909945	11:10673739-10673739	splice_polypyrimidine_tract_variant,intron_variant,non_coding_transcript_variant	LOW	*MRVI1*	Transcript	processed_transcript
rs4909945	11:10673739-10673739	5_prime_UTR_variant	MODIFIER	*MRVI1*	Transcript	protein_coding
rs4909945	11:10673739-10673739	5_prime_UTR_variant	MODIFIER	*MRVI1*	Transcript	protein_coding
rs4909945	11:10673739-10673739	splice_polypyrimidine_tract_variant,intron_variant	LOW	*MRVI1*	Transcript	protein_coding
rs4909945	11:10673739-10673739	splice_polypyrimidine_tract_variant,intron_variant	LOW	*MRVI1*	Transcript	protein_coding
rs4909945	11:10673739-10673739	5_prime_UTR_variant	MODIFIER	*MRVI1*	Transcript	protein_coding
rs4909945	11:10673739-10673739	5_prime_UTR_variant	MODIFIER	*MRVI1*	Transcript	protein_coding
rs4909945	11:10673739-10673739	intron_variant	MODIFIER	*MRVI1*	Transcript	protein_coding
rs4909945	11:10673739-10673739	intron_variant	MODIFIER	*MRVI1*	Transcript	protein_coding
rs4909945	11:10673739-10673739	5_prime_UTR_variant	MODIFIER	*MRVI1*	Transcript	protein_coding

rs4909945	11:10673739-10673739	5_prime_UTR_variant	MODIFIER	*MRVI1*	Transcript	protein_coding
rs4909945	11:10673739-10673739	splice_polypyrimidine_tract_variant,intron_variant	LOW	*MRVI1*	Transcript	protein_coding
rs4909945	11:10673739-10673739	splice_polypyrimidine_tract_variant,intron_variant	LOW	*MRVI1*	Transcript	protein_coding
rs1971819	2:203705787-203705787	intron_variant	MODIFIER	*ICA1L*	Transcript	protein_coding
rs1971819	2:203705787-203705787	intron_variant	MODIFIER	*ICA1L*	Transcript	protein_coding
rs1971819	2:203705787-203705787	intron_variant	MODIFIER	*ICA1L*	Transcript	protein_coding
rs1971819	2:203705787-203705787	intron_variant	MODIFIER	*ICA1L*	Transcript	protein_coding
rs1971819	2:203705787-203705787	intron_variant	MODIFIER	*ICA1L*	Transcript	protein_coding
rs1971819	2:203705787-203705787	intron_variant	MODIFIER	*ICA1L*	Transcript	protein_coding
rs1971819	2:203705787-203705787	intron_variant	MODIFIER	*ICA1L*	Transcript	protein_coding
rs1971819	2:203705787-203705787	intron_variant	MODIFIER	*ICA1L*	Transcript	protein_coding
rs1971819	2:203705787-203705787	intron_variant	MODIFIER	*ICA1L*	Transcript	protein_coding
rs1971819	2:203705787-203705787	intron_variant	MODIFIER	*ICA1L*	Transcript	protein_coding
rs1971819	2:203705787-203705787	intron_variant	MODIFIER	*ICA1L*	Transcript	protein_coding
rs1971819	2:203705787-203705787	intron_variant	MODIFIER	*ICA1L*	Transcript	protein_coding
rs1971819	2:203705787-203705787	intron_variant	MODIFIER	*ICA1L*	Transcript	protein_coding
rs1971819	2:203705787-203705787	intron_variant	MODIFIER	*ICA1L*	Transcript	protein_coding
rs1971819	2:203705787-203705787	intron_variant	MODIFIER	*ICA1L*	Transcript	protein_coding
rs1971819	2:203705787-203705787	intron_variant	MODIFIER	*ICA1L*	Transcript	protein_coding
rs1971819	2:203705787-203705787	intron_variant	MODIFIER	*ICA1L*	Transcript	protein_coding
rs1971819	2:203705787-203705787	intron_variant	MODIFIER	*ICA1L*	Transcript	protein_coding
rs1971819	2:203705787-203705787	non_coding_transcript_exon_variant	MODIFIER	*KRT8P15*	Transcript	processed_pseudogene
rs1971819	2:203705787-203705787	non_coding_transcript_exon_variant	MODIFIER	*KRT8P15*	Transcript	processed_pseudogene
rs1971819	2:203705787-203705787	intron_variant	MODIFIER	*ICA1L*	Transcript	protein_coding
rs1971819	2:203705787-203705787	intron_variant	MODIFIER	*ICA1L*	Transcript	protein_coding
rs1971819	2:203705787-203705787	intron_variant	MODIFIER	*ICA1L*	Transcript	protein_coding
rs1971819	2:203705787-203705787	intron_variant	MODIFIER	*ICA1L*	Transcript	protein_coding
rs1971819	2:203705787-203705787	intron_variant,NMD_transcript_variant	MODIFIER	*ICA1L*	Transcript	nonsense_mediated_decay
rs1971819	2:203705787-203705787	intron_variant,NMD_transcript_variant	MODIFIER	*ICA1L*	Transcript	nonsense_mediated_decay
rs1971819	2:203705787-203705787	intron_variant,NMD_transcript_variant	MODIFIER	*ICA1L*	Transcript	nonsense_mediated_decay
rs1971819	2:203705787-203705787	intron_variant,NMD_transcript_variant	MODIFIER	*ICA1L*	Transcript	nonsense_mediated_decay
rs1971819	2:203705787-203705787	intron_variant	MODIFIER	*ICA1L*	Transcript	protein_coding
rs1971819	2:203705787-203705787	intron_variant	MODIFIER	*ICA1L*	Transcript	protein_coding
rs1971819	2:203705787-203705787	intron_variant	MODIFIER	*ICA1L*	Transcript	protein_coding
rs1971819	2:203705787-203705787	intron_variant	MODIFIER	*ICA1L*	Transcript	protein_coding
rs1971819	2:203705787-203705787	intron_variant	MODIFIER	*ICA1L*	Transcript	protein_coding
rs1971819	2:203705787-203705787	intron_variant	MODIFIER	*ICA1L*	Transcript	protein_coding
rs1971819	2:203705787-203705787	intron_variant	MODIFIER	*ICA1L*	Transcript	protein_coding
rs1971819	2:203705787-203705787	intron_variant	MODIFIER	*ICA1L*	Transcript	protein_coding
rs1971819	2:203705787-203705787	intron_variant,non_coding_transcript_variant	MODIFIER	*ICA1L*	Transcript	retained_intron
rs1971819	2:203705787-203705787	intron_variant,non_coding_transcript_variant	MODIFIER	*ICA1L*	Transcript	retained_intron
rs1047891	2:211540507-211540507	missense_variant	MODERATE	*CPS1*	Transcript	protein_coding
rs1047891	2:211540507-211540507	missense_variant	MODERATE	*CPS1*	Transcript	protein_coding
rs1047891	2:211540507-211540507	missense_variant	MODERATE	*CPS1*	Transcript	protein_coding
rs1047891	2:211540507-211540507	non_coding_transcript_exon_variant	MODIFIER	*CPS1*	Transcript	retained_intron

CKD, chronic kidney disease; eGFR, estimated glomerular filtration rate; UACR, urinary albumin-to-creatinine ratio.

Supplementary Table 13. Fine-mapping 99% credible-set of index SNP from cross-trait meta-analysis between migraine and chronic kidney disease.

Cross-trait GWAS model	Index SNP	Credible-set SNPs	CHR	BP	P-CPASSOC	probNorm	cumSum
Migraine and CKD	rs1047891	rs1047891	2	211540507	6.13E-12	0.8744	0.8744
		rs715	2	211543055	4.51E-11	0.1239	0.9983
Migraine and eGFR	rs1047891	rs1047891	2	211540507	9.35E-65	0.9999	0.9999
	rs13099628	rs13099628	3	38927854	4.18E-10	0.0812	0.0812
		rs4320030	3	38928112	4.30E-10	0.0790	0.1603
		rs33985936	3	38936134	5.17E-10	0.0660	0.2263
		rs11919589	3	38935192	5.23E-10	0.0653	0.2915
		rs11915204	3	38908127	5.43E-10	0.0630	0.3545
		rs13099670	3	38936829	6.33E-10	0.0542	0.4087
		rs35752744	3	38926290	6.39E-10	0.0537	0.4624
		rs11924818	3	38922323	7.41E-10	0.0465	0.5089
		rs13080116	3	38907223	7.51E-10	0.0459	0.5548
		rs13079538	3	38906903	7.54E-10	0.0457	0.6005
		rs4371451	3	38906037	7.88E-10	0.0438	0.6442
		rs13088577	3	38929631	8.09E-10	0.0427	0.6869
		rs3923518	3	38886413	8.32E-10	0.0415	0.7284
		rs13095260	3	38930889	8.42E-10	0.0410	0.7695
		rs4462889	3	38884451	8.73E-10	0.0396	0.8091
		rs4676477	3	38881195	9.07E-10	0.0381	0.8472
		rs4676590	3	38881080	1.03E-09	0.0336	0.8808
		rs13079727	3	38915975	1.15E-09	0.0303	0.9111
		rs4527319	3	38880142	1.40E-09	0.0249	0.9360
		rs11928473	3	38897271	1.54E-09	0.0228	0.9588
		rs4284929	3	38889656	1.92E-09	0.0183	0.9771
		rs13062806	3	38877793	2.61E-09	0.0136	0.9908
	rs1566225	rs1566225	1	150415990	2.61E-15	0.0277	0.0277
		rs3850844	1	150413940	3.33E-15	0.0218	0.0495
		rs12736375	1	150411912	3.58E-15	0.0203	0.0699
		rs11205373	1	150404708	3.80E-15	0.0191	0.0890
		rs834234	1	150352494	3.85E-15	0.0189	0.1079
		rs11205375	1	150412474	3.86E-15	0.0189	0.1268
		rs12731296	1	150411787	3.96E-15	0.0184	0.1452
		rs7513182	1	150442612	4.34E-15	0.0168	0.1620
		rs834237	1	150363930	4.48E-15	0.0163	0.1783
		rs4451552	1	150455854	4.77E-15	0.0153	0.1937

rs10888583	1	150439877	5.03E-15	0.0145	0.2082
rs1260424	1	150361349	5.14E-15	0.0142	0.2224
rs2275246	1	150460168	5.19E-15	0.0141	0.2365
rs11205371	1	150398510	5.21E-15	0.0140	0.2506
rs35705743	1	150451832	5.23E-15	0.0140	0.2646
rs1313570	1	150342642	5.43E-15	0.0135	0.2781
rs11581786	1	150425580	5.47E-15	0.0134	0.2915
rs4926395	1	150405298	5.56E-15	0.0132	0.3046
rs1260420	1	150391345	5.77E-15	0.0127	0.3173
rs5011187	1	150422761	5.90E-15	0.0124	0.3298
rs7411534	1	150447269	5.93E-15	0.0124	0.3421
rs834235	1	150362154	5.95E-15	0.0123	0.3545
rs4926435	1	150419865	5.98E-15	0.0123	0.3667
rs11205370	1	150397567	6.07E-15	0.0121	0.3788
rs4970971	1	150468359	6.17E-15	0.0119	0.3907
rs832621	1	150392909	6.28E-15	0.0117	0.4024
rs6699093	1	150440451	6.34E-15	0.0116	0.4140
rs4970964	1	150458029	6.35E-15	0.0116	0.4255
rs11205382	1	150442466	6.72E-15	0.0109	0.4365
rs4970922	1	150458094	6.78E-15	0.0108	0.4473
rs1260419	1	150390524	6.99E-15	0.0105	0.4578
rs834242	1	150379858	6.99E-15	0.0105	0.4683
rs2133129	1	150355537	7.20E-15	0.0102	0.4785
rs1260458	1	150347454	7.27E-15	0.0101	0.4887
rs698922	1	150379309	7.29E-15	0.0101	0.4988
rs1313568	1	150344180	7.29E-15	0.0101	0.5088
rs6664703	1	150459087	7.31E-15	0.0101	0.5189
rs4446975	1	150456169	7.33E-15	0.0100	0.5289
rs863862	1	150352361	7.42E-15	0.0099	0.5389
rs834243	1	150339290	7.47E-15	0.0098	0.5487
rs828784	1	150335240	8.00E-15	0.0092	0.5579
rs1313569	1	150344171	8.05E-15	0.0091	0.5671
rs1097071	1	150332870	8.10E-15	0.0091	0.5762
rs834238	1	150366469	8.12E-15	0.0091	0.5852
rs1260404	1	150304150	8.16E-15	0.0090	0.5943
rs1694364	1	150321066	8.31E-15	0.0089	0.6031
rs1260408	1	150307270	8.33E-15	0.0089	0.6120
rs1932934	1	150449195	8.33E-15	0.0088	0.6208
rs2012751	1	150438266	8.42E-15	0.0088	0.6296
rs698921	1	150379517	8.57E-15	0.0086	0.6382

rs2264417	1	150368501	9.00E-15	0.0082	0.6464
rs1260457	1	150347374	9.01E-15	0.0082	0.6546
rs1694386	1	150377965	9.06E-15	0.0081	0.6627
rs9436009	1	150459426	9.11E-15	0.0081	0.6708
rs9436118	1	150462811	9.32E-15	0.0079	0.6788
rs1776272	1	150311042	9.69E-15	0.0076	0.6864
rs1260409	1	150308136	9.73E-15	0.0076	0.6940
rs1694380	1	150292341	9.81E-15	0.0075	0.7015
rs696617	1	150376311	9.92E-15	0.0075	0.7090
rs2454285	1	150290609	9.95E-15	0.0074	0.7164
rs1260385	1	150316385	1.00E-14	0.0074	0.7238
rs698914	1	150298750	1.01E-14	0.0073	0.7311
rs1776265	1	150323522	1.02E-14	0.0072	0.7384
rs8006	1	150280801	1.02E-14	0.0072	0.7456
rs580159	1	150276650	1.03E-14	0.0072	0.7528
rs1260403	1	150303666	1.03E-14	0.0072	0.7600
rs1262432	1	150371839	1.03E-14	0.0072	0.7672
rs834225	1	150300727	1.04E-14	0.0071	0.7743
rs698917	1	150319884	1.06E-14	0.0070	0.7813
rs1097069	1	150330190	1.08E-14	0.0068	0.7881
rs6684939	1	150440563	1.08E-14	0.0068	0.7949
rs1747927	1	150284940	1.09E-14	0.0068	0.8017
rs1694381	1	150292122	1.10E-14	0.0068	0.8085
rs578353	1	150287884	1.12E-14	0.0066	0.8151
rs1097066	1	150320717	1.13E-14	0.0065	0.8216
rs1260391	1	150373077	1.18E-14	0.0063	0.8279
rs696615	1	150338988	1.19E-14	0.0063	0.8342
rs488271	1	150284534	1.23E-14	0.0060	0.8402
rs471738	1	150276429	1.34E-14	0.0055	0.8457
rs11205359	1	150277387	1.35E-14	0.0055	0.8512
rs6678973	1	150398064	1.39E-14	0.0054	0.8566
rs834227	1	150383768	1.42E-14	0.0052	0.8618
rs12023277	1	150466737	1.45E-14	0.0051	0.8669
rs509345	1	150276022	1.56E-14	0.0048	0.8717
rs519126	1	150288859	1.57E-14	0.0047	0.8765
rs10888584	1	150476406	1.59E-14	0.0047	0.8811
rs1260401	1	150303269	1.60E-14	0.0046	0.8858
rs698918	1	150319762	1.65E-14	0.0045	0.8903
rs1776276	1	150293078	1.71E-14	0.0044	0.8947
rs12747669	1	150268622	1.77E-14	0.0042	0.8989

	rs543179	1	150274610	1.83E-14	0.0041	0.9030
	rs3125808	1	150314272	1.89E-14	0.0039	0.9069
	rs2762860	1	150273579	1.90E-14	0.0039	0.9109
	rs9436120	1	150473084	1.91E-14	0.0039	0.9148
	rs1260373	1	150331866	1.97E-14	0.0038	0.9186
	rs1815545	1	150479977	2.00E-14	0.0037	0.9223
	rs573351	1	150271080	2.06E-14	0.0036	0.9259
	rs471657	1	150276461	2.07E-14	0.0036	0.9295
	rs494041	1	150270026	2.15E-14	0.0035	0.9330
	rs3754217	1	150480373	2.19E-14	0.0034	0.9364
	rs3818978	1	150266338	2.21E-14	0.0034	0.9398
	rs12049177	1	150474337	2.22E-14	0.0034	0.9432
	rs496203	1	150270025	2.32E-14	0.0032	0.9464
	rs1260398	1	150279876	2.37E-14	0.0032	0.9496
	rs1694379	1	150292417	2.39E-14	0.0031	0.9527
	rs698920	1	150386023	2.48E-14	0.0030	0.9558
	rs1260407	1	150306125	2.49E-14	0.0030	0.9588
	rs4970963	1	150456389	2.51E-14	0.0030	0.9618
	rs834241	1	150379007	2.54E-14	0.0030	0.9647
	rs7554686	1	150265619	2.68E-14	0.0028	0.9675
	rs11590406	1	150327105	2.91E-14	0.0026	0.9701
	rs7553647	1	150472666	2.93E-14	0.0026	0.9727
	rs11811885	1	150268461	3.05E-14	0.0025	0.9751
	rs1776275	1	150269437	3.70E-14	0.0020	0.9772
	rs494952	1	150269932	3.79E-14	0.0020	0.9792
	rs9661040	1	150483236	4.36E-14	0.0017	0.9809
	rs1694390	1	150380780	4.40E-14	0.0017	0.9826
	rs1776273	1	150270209	4.59E-14	0.0017	0.9843
	rs4970979	1	150483840	5.08E-14	0.0015	0.9858
	rs12093148	1	150265173	5.81E-14	0.0013	0.9871
	rs875514	1	150484555	6.52E-14	0.0012	0.9883
	rs3754214	1	150478623	6.85E-14	0.0011	0.9894
	rs1776270	1	150369337	8.11E-14	0.0009	0.9903
	rs41272663	2	211302627	2.91E-12	0.3047	0.3047
	rs2287431	2	211320288	8.51E-12	0.1064	0.4111
	rs17822981	2	211355495	3.44E-11	0.0271	0.4382
rs41272663	rs13031561	2	211348330	3.47E-11	0.0269	0.4651
	rs12468052	2	211351479	3.48E-11	0.0268	0.4919
	rs56098529	2	211397176	4.15E-11	0.0225	0.5144
	rs72932418	2	211349092	4.17E-11	0.0225	0.5369

rs72932424	2	211350709	4.29E-11	0.0218	0.5587
rs28485712	2	211347806	4.59E-11	0.0204	0.5792
rs72932421	2	211349321	4.91E-11	0.0191	0.5983
rs10804184	2	211359008	5.50E-11	0.0171	0.6155
rs12463895	2	211351321	5.62E-11	0.0168	0.6322
rs6729280	2	211354906	6.53E-11	0.0145	0.6467
rs72932430	2	211352269	6.80E-11	0.0139	0.6607
rs72932437	2	211353402	6.84E-11	0.0138	0.6745
rs17771664	2	211353114	6.85E-11	0.0138	0.6883
rs6725979	2	211354552	6.96E-11	0.0136	0.7019
rs72932447	2	211355169	7.12E-11	0.0133	0.7152
rs72932427	2	211352180	7.25E-11	0.0131	0.7283
rs72934320	2	211362871	7.88E-11	0.0120	0.7404
rs1044708	2	211298180	7.99E-11	0.0119	0.7523
rs17772042	2	211368672	8.25E-11	0.0115	0.7638
rs72932449	2	211355390	8.32E-11	0.0114	0.7752
rs12619519	2	211364908	8.40E-11	0.0113	0.7865
rs72934330	2	211369994	8.56E-11	0.0111	0.7976
rs6711129	2	211399751	8.84E-11	0.0108	0.8084
rs10490318	2	211397725	8.94E-11	0.0106	0.8191
rs6725770	2	211354198	9.23E-11	0.0103	0.8294
rs4372818	2	211383444	9.34E-11	0.0102	0.8396
rs12611623	2	211391498	9.45E-11	0.0101	0.8497
rs6752320	2	211386936	9.73E-11	0.0098	0.8595
rs72932439	2	211353553	9.85E-11	0.0097	0.8692
rs16844534	2	211353821	1.04E-10	0.0092	0.8784
rs10804185	2	211428354	1.09E-10	0.0088	0.8872
rs150841467	2	211371045	1.11E-10	0.0086	0.8958
rs78421188	2	211374952	1.15E-10	0.0083	0.9041
rs72934339	2	211379580	1.38E-10	0.0070	0.9111
rs1812875	2	211290057	1.79E-10	0.0054	0.9165
rs4467201	2	211292054	1.84E-10	0.0053	0.9217
rs72934370	2	211407756	1.90E-10	0.0051	0.9268
rs3821136	2	211299687	2.15E-10	0.0045	0.9313
rs4673528	2	211311314	2.22E-10	0.0044	0.9357
rs2370956	2	211299869	2.49E-10	0.0039	0.9396
rs6435569	2	211289689	2.70E-10	0.0036	0.9432
rs12466705	2	211423777	2.72E-10	0.0036	0.9468
rs3770699	2	211304138	3.10E-10	0.0032	0.9500
rs17824552	2	211424880	3.16E-10	0.0031	0.9531

	rs11896108	2	211263835	3.86E-10	0.0026	0.9556
	rs1350463	2	211261619	4.15E-10	0.0024	0.9580
	rs11900468	2	211263526	4.58E-10	0.0022	0.9602
	rs11894867	2	211263532	4.97E-10	0.0020	0.9622
	rs2287432	2	211319824	5.24E-10	0.0019	0.9640
	rs2287424	2	211326209	5.41E-10	0.0018	0.9659
	rs6727968	2	211272011	5.92E-10	0.0017	0.9676
	rs3856339	2	211321473	6.15E-10	0.0016	0.9692
	rs12473150	2	211258845	6.23E-10	0.0016	0.9708
	rs3770695	2	211318591	6.98E-10	0.0014	0.9722
	rs4609985	2	211269157	7.12E-10	0.0014	0.9736
	rs7595022	2	211318778	8.86E-10	0.0011	0.9747
	rs3770689	2	211333956	9.14E-10	0.0011	0.9758
	rs10804182	2	211327877	9.24E-10	0.0011	0.9769
	rs3770694	2	211328499	9.66E-10	0.0010	0.9780
	rs4673529	2	211313186	1.01E-09	0.0010	0.9790
	rs7578688	2	211326855	1.01E-09	0.0010	0.9800
	rs2287425	2	211326134	1.01E-09	0.0010	0.9810
	rs6756422	2	211322945	1.01E-09	0.0010	0.9820
	rs6725303	2	211354027	1.02E-09	0.0010	0.9830
	rs3845633	2	211321200	1.07E-09	0.0009	0.9839
	rs1379837	2	211245928	1.08E-09	0.0009	0.9848
	rs10804183	2	211327901	1.10E-09	0.0009	0.9858
	rs6435568	2	211285002	1.10E-09	0.0009	0.9867
	rs2287430	2	211322121	1.12E-09	0.0009	0.9876
	rs2370949	2	211331073	1.12E-09	0.0009	0.9885
	rs2287429	2	211322394	1.13E-09	0.0009	0.9894
	rs2887899	2	211329956	1.16E-09	0.0009	0.9902
	rs62576116	9	119342218	8.05E-17	0.1651	0.1651
	rs62576078	9	119325576	1.61E-16	0.0835	0.2486
	rs12342512	9	119342293	1.81E-16	0.0743	0.3230
	rs4837616	9	119310999	3.77E-16	0.0361	0.3590
	rs62575439	9	119265926	4.95E-16	0.0276	0.3866
rs62576116	rs4837614	9	119310365	6.23E-16	0.0220	0.4086
	rs4837600	9	119296733	6.25E-16	0.0219	0.4305
	rs57101343	9	119274101	6.85E-16	0.0200	0.4505
	rs55904272	9	119305313	6.96E-16	0.0197	0.4702
	rs55904938	9	119292033	7.78E-16	0.0177	0.4878
	rs4836740	9	119296997	8.05E-16	0.0171	0.5049
	rs12002911	9	119268961	8.87E-16	0.0155	0.5204

rs34979631	9	119255297	9.64E-16	0.0143	0.5347
rs1830583	9	119284614	1.00E-15	0.0138	0.5485
rs4837579	9	119271465	1.12E-15	0.0123	0.5608
rs11999663	9	119284209	1.14E-15	0.0121	0.5729
rs58640933	9	119268192	1.27E-15	0.0109	0.5838
rs62574376	9	119291389	1.28E-15	0.0108	0.5946
rs62574369	9	119277178	1.41E-15	0.0098	0.6045
rs62574371	9	119281082	1.45E-15	0.0096	0.6140
rs1830582	9	119284304	1.49E-15	0.0093	0.6234
rs34278882	9	119257528	1.50E-15	0.0093	0.6326
rs74881632	9	119290687	1.50E-15	0.0092	0.6419
rs4837605	9	119299614	1.50E-15	0.0092	0.6511
rs4837604	9	119299346	1.67E-15	0.0083	0.6594
rs57691685	9	119280371	1.72E-15	0.0081	0.6675
rs4837589	9	119287922	1.72E-15	0.0081	0.6756
rs75148528	9	119283639	1.77E-15	0.0079	0.6834
rs59494978	9	119285336	1.84E-15	0.0075	0.6910
rs56042345	9	119471408	1.88E-15	0.0074	0.6984
rs9942928	9	119299839	1.93E-15	0.0072	0.7056
rs3891689	9	119258583	1.97E-15	0.0071	0.7127
rs62574375	9	119290976	1.98E-15	0.0070	0.7197
rs60203546	9	119473074	1.98E-15	0.0070	0.7267
rs76346015	9	119301290	2.02E-15	0.0069	0.7336
rs62574372	9	119282203	2.04E-15	0.0068	0.7405
rs62574187	9	119457995	2.44E-15	0.0057	0.7462
rs59325309	9	119293843	2.53E-15	0.0055	0.7517
rs57292742	9	119304002	2.65E-15	0.0053	0.7570
rs41308922	9	119449156	2.73E-15	0.0051	0.7621
rs57366433	9	119305993	2.95E-15	0.0048	0.7669
rs76973802	9	119450348	3.04E-15	0.0046	0.7715
rs55681291	9	119305347	3.04E-15	0.0046	0.7761
rs55695529	9	119262840	3.16E-15	0.0044	0.7805
rs56103734	9	119305424	3.58E-15	0.0039	0.7844
rs1441748	9	119474700	3.61E-15	0.0039	0.7883
rs7042951	9	119305421	3.88E-15	0.0036	0.7920
rs55842808	9	119305516	4.04E-15	0.0035	0.7955
rs62574193	9	119477732	4.14E-15	0.0034	0.7989
rs79413975	9	119262362	4.20E-15	0.0034	0.8022
rs55898437	9	119336054	4.58E-15	0.0031	0.8053
rs41308928	9	119306130	4.63E-15	0.0030	0.8083

rs62576117	9	119342410	4.64E-15	0.0030	0.8114
rs10983248	9	119342386	4.72E-15	0.0030	0.8144
rs62576118	9	119342438	4.80E-15	0.0029	0.8173
rs62576115	9	119342127	5.21E-15	0.0027	0.8200
rs55844217	9	119441560	5.29E-15	0.0027	0.8227
rs56014873	9	119343399	5.70E-15	0.0025	0.8252
rs10116835	9	119324583	5.73E-15	0.0025	0.8277
rs74715051	9	119429830	6.07E-15	0.0023	0.8300
rs16933693	9	119341583	6.31E-15	0.0022	0.8322
rs73655406	9	119328093	6.35E-15	0.0022	0.8345
rs4836758	9	119326719	6.42E-15	0.0022	0.8367
rs62574407	9	119312489	6.67E-15	0.0021	0.8388
rs73655416	9	119341343	6.76E-15	0.0021	0.8409
rs62576087	9	119329760	7.22E-15	0.0020	0.8429
rs59375457	9	119324581	7.27E-15	0.0020	0.8448
rs62576092	9	119332823	7.31E-15	0.0019	0.8468
rs60209746	9	119312475	7.58E-15	0.0019	0.8487
rs7869323	9	119341236	7.72E-15	0.0018	0.8505
rs58037465	9	119329779	7.98E-15	0.0018	0.8523
rs62576127	9	119366540	8.04E-15	0.0018	0.8541
rs62574401	9	119310066	8.08E-15	0.0018	0.8558
rs62576082	9	119327167	8.18E-15	0.0017	0.8576
rs73655405	9	119328087	8.58E-15	0.0017	0.8592
rs57297769	9	119440793	8.72E-15	0.0016	0.8608
rs4836774	9	119360508	9.09E-15	0.0016	0.8624
rs10118576	9	119317608	9.10E-15	0.0016	0.8640
rs58864523	9	119381297	9.11E-15	0.0016	0.8655
rs10120556	9	119316612	9.12E-15	0.0016	0.8671
rs10116236	9	119318872	9.13E-15	0.0016	0.8687
rs28411124	9	119315997	9.76E-15	0.0015	0.8701
rs61173206	9	119314201	9.90E-15	0.0014	0.8716
rs62576102	9	119339271	1.00E-14	0.0014	0.8730
rs41266675	9	119347032	1.00E-14	0.0014	0.8744
rs62576680	9	119382392	1.04E-14	0.0014	0.8758
rs4836759	9	119326760	1.05E-14	0.0014	0.8772
rs59983726	9	119324647	1.07E-14	0.0013	0.8785
rs12003311	9	119326174	1.08E-14	0.0013	0.8798
rs62576081	9	119325787	1.12E-14	0.0013	0.8811
rs7872812	9	119341544	1.13E-14	0.0013	0.8824
rs4836756	9	119326375	1.13E-14	0.0013	0.8836

rs61654164	9	119334290	1.14E-14	0.0013	0.8849
rs55920375	9	119324932	1.14E-14	0.0013	0.8862
rs4837622	9	119322646	1.15E-14	0.0012	0.8874
rs62576080	9	119325640	1.16E-14	0.0012	0.8886
rs56728425	9	119325489	1.17E-14	0.0012	0.8899
rs56367245	9	119333696	1.19E-14	0.0012	0.8911
rs4836761	9	119326823	1.19E-14	0.0012	0.8923
rs62574194	9	119479868	1.20E-14	0.0012	0.8935
rs4837634	9	119332516	1.20E-14	0.0012	0.8947
rs4837641	9	119338307	1.20E-14	0.0012	0.8958
rs60853238	9	119351967	1.21E-14	0.0012	0.8970
rs4836764	9	119331370	1.21E-14	0.0012	0.8982
rs73655411	9	119333192	1.22E-14	0.0012	0.8994
rs62574413	9	119318928	1.22E-14	0.0012	0.9006
rs62574414	9	119319025	1.22E-14	0.0012	0.9017
rs62574423	9	119323249	1.22E-14	0.0012	0.9029
rs61520570	9	119322506	1.22E-14	0.0012	0.9041
rs62574403	9	119311400	1.22E-14	0.0012	0.9053
rs58494876	9	119328378	1.23E-14	0.0012	0.9064
rs62576686	9	119408202	1.24E-14	0.0012	0.9076
rs55962567	9	119322251	1.24E-14	0.0012	0.9087
rs58637290	9	119325456	1.24E-14	0.0012	0.9099
rs4836755	9	119326282	1.25E-14	0.0012	0.9110
rs61700493	9	119322554	1.25E-14	0.0011	0.9122
rs62576133	9	119372733	1.25E-14	0.0011	0.9133
rs16933712	9	119343698	1.25E-14	0.0011	0.9145
rs62576086	9	119329158	1.27E-14	0.0011	0.9156
rs58577541	9	119318382	1.27E-14	0.0011	0.9167
rs9969846	9	119330272	1.27E-14	0.0011	0.9179
rs9969847	9	119330375	1.28E-14	0.0011	0.9190
rs56265170	9	119314382	1.28E-14	0.0011	0.9201
rs4836753	9	119321131	1.28E-14	0.0011	0.9212
rs57557118	9	119311726	1.28E-14	0.0011	0.9223
rs55966826	9	119398965	1.29E-14	0.0011	0.9234
rs73655414	9	119336613	1.29E-14	0.0011	0.9246
rs58531689	9	119322447	1.29E-14	0.0011	0.9257
rs58703270	9	119318782	1.30E-14	0.0011	0.9268
rs4836746	9	119310564	1.31E-14	0.0011	0.9279
rs60716541	9	119325586	1.32E-14	0.0011	0.9289
rs4837633	9	119332511	1.32E-14	0.0011	0.9300

rs55651724	9	119314186	1.32E-14	0.0011	0.9311
rs73655407	9	119329247	1.32E-14	0.0011	0.9322
rs60542839	9	119328532	1.33E-14	0.0011	0.9333
rs73655408	9	119329434	1.33E-14	0.0011	0.9344
rs9969799	9	119330515	1.33E-14	0.0011	0.9354
rs4836765	9	119331477	1.34E-14	0.0011	0.9365
rs1570197	9	119337861	1.36E-14	0.0011	0.9376
rs4836766	9	119334663	1.37E-14	0.0010	0.9386
rs62576101	9	119336678	1.38E-14	0.0010	0.9397
rs55673802	9	119323973	1.39E-14	0.0010	0.9407
rs57610989	9	119325522	1.40E-14	0.0010	0.9417
rs62574410	9	119317660	1.40E-14	0.0010	0.9427
rs16933729	9	119351849	1.41E-14	0.0010	0.9438
rs56848636	9	119322309	1.41E-14	0.0010	0.9448
rs1570199	9	119340133	1.42E-14	0.0010	0.9458
rs4836763	9	119331287	1.42E-14	0.0010	0.9468
rs58280438	9	119316813	1.42E-14	0.0010	0.9478
rs112965656	9	119394210	1.42E-14	0.0010	0.9488
rs55974177	9	119393423	1.43E-14	0.0010	0.9498
rs4836748	9	119314116	1.43E-14	0.0010	0.9508
rs4837621	9	119321202	1.43E-14	0.0010	0.9518
rs56023289	9	119349430	1.44E-14	0.0010	0.9528
rs73655468	9	119434113	1.45E-14	0.0010	0.9538
rs73655409	9	119329636	1.46E-14	0.0010	0.9548
rs41305463	9	119320107	1.46E-14	0.0010	0.9558
rs58077965	9	119333916	1.47E-14	0.0010	0.9568
rs4837620	9	119320945	1.47E-14	0.0010	0.9578
rs62576084	9	119328257	1.49E-14	0.0010	0.9587
rs59089694	9	119381049	1.49E-14	0.0010	0.9597
rs57243279	9	119340703	1.50E-14	0.0010	0.9607
rs73655413	9	119335554	1.50E-14	0.0010	0.9616
rs41266665	9	119330792	1.52E-14	0.0009	0.9626
rs59590954	9	119314131	1.54E-14	0.0009	0.9635
rs60933913	9	119333441	1.55E-14	0.0009	0.9644
rs62576091	9	119332813	1.55E-14	0.0009	0.9654
rs9969759	9	119327784	1.57E-14	0.0009	0.9663
rs1358860	9	119350446	1.58E-14	0.0009	0.9672
rs28688239	9	119339257	1.59E-14	0.0009	0.9681
rs60542122	9	119324994	1.59E-14	0.0009	0.9690
rs62576083	9	119327236	1.60E-14	0.0009	0.9699

	rs56232900	9	119335857	1.60E-14	0.0009	0.9708
	rs10121095	9	119329446	1.61E-14	0.0009	0.9717
	rs62574188	9	119464286	1.62E-14	0.0009	0.9726
	rs4837615	9	119310901	1.62E-14	0.0009	0.9735
	rs60772419	9	119438751	1.63E-14	0.0009	0.9743
	rs4836749	9	119314889	1.64E-14	0.0009	0.9752
	rs59443045	9	119324367	1.66E-14	0.0009	0.9761
	rs60949535	9	119336964	1.66E-14	0.0009	0.9770
	rs10121441	9	119346029	1.70E-14	0.0008	0.9778
	rs16933688	9	119337406	1.73E-14	0.0008	0.9786
	rs62574405	9	119311468	1.74E-14	0.0008	0.9795
	rs62574416	9	119319201	1.80E-14	0.0008	0.9803
	rs4836760	9	119326799	1.80E-14	0.0008	0.9811
	rs62574429	9	119324418	1.80E-14	0.0008	0.9819
	rs28410315	9	119338898	1.83E-14	0.0008	0.9827
	rs57454229	9	119441237	1.87E-14	0.0008	0.9834
	rs7872204	9	119345154	1.87E-14	0.0008	0.9842
	rs4836752	9	119315203	1.89E-14	0.0008	0.9850
	rs57816386	9	119441586	1.90E-14	0.0008	0.9857
	rs7867264	9	119343617	1.92E-14	0.0008	0.9865
	rs58653571	9	119340643	1.92E-14	0.0008	0.9872
	rs12347431	9	119336189	1.94E-14	0.0007	0.9880
	rs55671228	9	119405831	1.95E-14	0.0007	0.9887
	rs12341354	9	119348942	1.97E-14	0.0007	0.9894
	rs1570198	9	119339800	2.18E-14	0.0007	0.9901
	rs6776700	3	48496758	2.95E-15	0.0430	0.0430
	rs9826247	3	48497145	3.22E-15	0.0394	0.0824
	rs13091785	3	48494098	3.38E-15	0.0375	0.1199
	rs6442119	3	48440178	4.54E-15	0.0281	0.1480
	rs6442123	3	48500286	4.56E-15	0.0280	0.1759
	rs6770470	3	48435123	4.77E-15	0.0267	0.2026
	rs2242150	3	48505964	4.85E-15	0.0263	0.2289
rs6776700	rs6800475	3	48492541	4.87E-15	0.0262	0.2551
	rs1109227	3	48479207	5.06E-15	0.0252	0.2803
	rs13314659	3	48449149	5.49E-15	0.0233	0.3036
	rs9883759	3	48463618	5.61E-15	0.0228	0.3264
	rs7126	3	48485493	5.66E-15	0.0226	0.3490
	rs34761139	3	48427979	5.66E-15	0.0226	0.3716
	rs12487542	3	48426076	5.72E-15	0.0223	0.3939
	rs28824259	3	48425675	6.02E-15	0.0213	0.4152

rs6804774	3	48430617	6.06E-15	0.0211	0.4363
rs73074358	3	48427247	6.08E-15	0.0211	0.4574
rs6442120	3	48464503	6.44E-15	0.0199	0.4773
rs6796491	3	48430465	6.57E-15	0.0195	0.4968
rs9809843	3	48434398	6.65E-15	0.0193	0.5160
rs9864815	3	48434063	6.67E-15	0.0192	0.5352
rs1037773	3	48467902	6.69E-15	0.0192	0.5544
rs9862575	3	48438571	7.01E-15	0.0183	0.5727
rs1563736	3	48436794	7.21E-15	0.0178	0.5905
rs1975844	3	48442959	7.34E-15	0.0175	0.6080
rs9876891	3	48481434	7.40E-15	0.0173	0.6254
rs2279077	3	48474249	7.55E-15	0.0170	0.6424
rs9864371	3	48426766	7.64E-15	0.0168	0.6592
rs13324374	3	48484890	7.89E-15	0.0163	0.6755
rs9826195	3	48433635	8.21E-15	0.0157	0.6911
rs3774808	3	48481647	8.25E-15	0.0156	0.7067
rs3214041	3	48454468	8.56E-15	0.0150	0.7217
rs10470686	3	48449956	8.70E-15	0.0148	0.7365
rs6442118	3	48440047	8.89E-15	0.0145	0.7510
rs9883927	3	48463711	9.03E-15	0.0143	0.7653
rs11130170	3	48449897	9.20E-15	0.0140	0.7793
rs11130171	3	48462461	9.86E-15	0.0131	0.7924
rs13076076	3	48479039	1.01E-14	0.0128	0.8052
rs7618883	3	48498456	1.05E-14	0.0123	0.8175
rs2045554	3	48494542	1.09E-14	0.0119	0.8293
rs6810060	3	48434748	1.10E-14	0.0118	0.8411
rs2290822	3	48473204	1.18E-14	0.0110	0.8521
rs6794875	3	48455626	1.24E-14	0.0105	0.8625
rs6442124	3	48505302	1.32E-14	0.0098	0.8724
rs9881491	3	48487911	1.36E-14	0.0096	0.8819
rs9817615	3	48470756	1.64E-14	0.0079	0.8899
rs6784322	3	48422235	1.74E-14	0.0075	0.8974
rs7634377	3	48421258	1.82E-14	0.0072	0.9045
rs7639743	3	48482506	1.84E-14	0.0071	0.9116
rs1870444	3	48486773	2.22E-14	0.0059	0.9175
rs9311423	3	48420308	2.33E-14	0.0056	0.9231
rs1459249	3	48479618	2.39E-14	0.0055	0.9286
rs9311424	3	48420314	2.41E-14	0.0054	0.9340
rs7635522	3	48423656	2.45E-14	0.0053	0.9393
rs2362450	3	48461313	2.46E-14	0.0053	0.9446

		rs7653691	3	48419689	2.68E-14	0.0049	0.9495
		rs725309	3	48418708	3.33E-14	0.0040	0.9535
		rs6779262	3	48416186	3.64E-14	0.0036	0.9571
		rs9812647	3	48448914	3.67E-14	0.0036	0.9607
		rs725310	3	48418571	4.05E-14	0.0033	0.9639
		rs7630741	3	48419723	4.19E-14	0.0032	0.9671
		rs2885510	3	48418226	4.28E-14	0.0031	0.9702
		rs11797	3	48508585	4.28E-14	0.0031	0.9733
		rs4858793	3	48415007	5.05E-14	0.0026	0.9759
		rs922075	3	48489398	6.08E-14	0.0022	0.9781
		rs7636782	3	48421387	6.32E-14	0.0021	0.9802
		rs6442116	3	48415903	6.56E-14	0.0020	0.9822
		rs9838618	3	48487353	6.74E-14	0.0020	0.9842
		rs7636044	3	48484560	6.83E-14	0.0019	0.9861
		rs13069724	3	48478039	7.13E-14	0.0019	0.9880
		rs4858817	3	48416756	7.33E-14	0.0018	0.9898
		rs898225	3	48413179	7.48E-14	0.0018	0.9916
	rs9894634	rs9894634	17	1967501	6.16E-13	0.2208	0.2208
		rs6503222	17	1977862	9.02E-13	0.1519	0.3727
		rs9901671	17	1978484	9.63E-13	0.1426	0.5153
		rs2236374	17	1989637	1.16E-12	0.1187	0.6340
		rs8078625	17	1978334	1.46E-12	0.0950	0.7290
		rs4790311	17	1979188	1.46E-12	0.0946	0.8236
		rs9908259	17	1983106	2.28E-12	0.0612	0.8848
		rs9900967	17	2008278	2.62E-12	0.0534	0.9382
		rs2131704	17	1970898	5.67E-12	0.0250	0.9633
		rs9303241	17	1978963	1.64E-11	0.0088	0.9721
		rs3760230	17	1994071	2.01E-11	0.0073	0.9794
		rs9901806	17	1959822	3.53E-11	0.0042	0.9835
		rs9906546	17	1995614	3.92E-11	0.0038	0.9873
		rs4790310	17	1966920	4.81E-11	0.0031	0.9904
Migraine and UACR	rs1047891	rs1047891	2	211540507	1.06E-21	0.9936	0.9936
	rs1971819	rs1971819	2	203705787	1.03E-19	0.3650	0.3650
		rs10207567	2	203714973	1.22E-19	0.3075	0.6725
		rs12693975	2	203720745	1.79E-19	0.2118	0.8843
		rs934287	2	203708307	4.27E-19	0.0895	0.9737
		rs140244541	2	203808532	4.23E-17	0.0010	0.9747
		rs72934734	2	203739970	8.67E-17	0.0005	0.9752
		rs72934740	2	203741362	1.49E-16	0.0003	0.9754

rs146973310	2	203740270	1.50E-16	0.0003	0.9757
rs72934732	2	203739856	1.51E-16	0.0003	0.9760
rs72934738	2	203740861	1.54E-16	0.0003	0.9763
rs72934729	2	203737770	1.55E-16	0.0003	0.9765
rs72934737	2	203740798	1.58E-16	0.0003	0.9768
rs79539678	2	203740938	1.58E-16	0.0003	0.9770
rs72934735	2	203740010	1.59E-16	0.0003	0.9773
rs3845800	2	203734365	1.84E-16	0.0002	0.9775
rs4510208	2	203734866	1.91E-16	0.0002	0.9777
rs72934519	2	203939640	2.15E-16	0.0002	0.9779
rs114395475	2	203769112	2.18E-16	0.0002	0.9781
rs72934554	2	203987806	2.24E-16	0.0002	0.9783
rs72932731	2	203650410	2.27E-16	0.0002	0.9785
rs115654617	2	203893999	2.29E-16	0.0002	0.9787
rs72934551	2	203984117	2.30E-16	0.0002	0.9789
rs151316549	2	203931597	2.36E-16	0.0002	0.9790
rs115810193	2	203943168	2.41E-16	0.0002	0.9792
rs72936860	2	203783484	2.50E-16	0.0002	0.9794
rs6705330	2	203662197	2.57E-16	0.0002	0.9795
rs115953525	2	203744445	2.58E-16	0.0002	0.9797
rs4675310	2	203880834	2.63E-16	0.0002	0.9799
rs80087860	2	203673072	2.70E-16	0.0002	0.9800
rs72934510	2	203925360	2.74E-16	0.0002	0.9802
rs35212307	2	203765756	2.75E-16	0.0002	0.9803
rs72934513	2	203927587	2.75E-16	0.0002	0.9805
rs6722332	2	203745327	2.77E-16	0.0002	0.9806
rs2351524	2	203880992	2.79E-16	0.0001	0.9808
rs72934512	2	203926271	2.80E-16	0.0001	0.9809
rs6725887	2	203745885	2.81E-16	0.0001	0.9811
rs142013255	2	203967197	2.83E-16	0.0001	0.9812
rs142250318	2	203795762	2.85E-16	0.0001	0.9814
rs72934546	2	203980033	2.90E-16	0.0001	0.9815
rs72934518	2	203937908	2.94E-16	0.0001	0.9816
rs72934505	2	203916487	2.97E-16	0.0001	0.9818
rs78907692	2	203932176	3.03E-16	0.0001	0.9819
rs72926787	2	203818299	3.06E-16	0.0001	0.9821
rs114899426	2	203771652	3.07E-16	0.0001	0.9822
rs72936842	2	203773686	3.07E-16	0.0001	0.9823
rs72926783	2	203815740	3.08E-16	0.0001	0.9825

rs72934537	2	203969504	3.14E-16	0.0001	0.9826
rs77931721	2	203763076	3.15E-16	0.0001	0.9827
rs6723704	2	203738664	3.15E-16	0.0001	0.9829
rs116678869	2	203819471	3.16E-16	0.0001	0.9830
rs72934573	2	204005072	3.20E-16	0.0001	0.9831
rs78128841	2	203663975	3.21E-16	0.0001	0.9833
rs72936852	2	203775475	3.23E-16	0.0001	0.9834
rs72934745	2	203744454	3.23E-16	0.0001	0.9835
rs139333388	2	203952059	3.24E-16	0.0001	0.9836
rs72936834	2	203771260	3.26E-16	0.0001	0.9838
rs72932745	2	203662888	3.26E-16	0.0001	0.9839
rs72932741	2	203661048	3.26E-16	0.0001	0.9840
rs72932746	2	203663498	3.27E-16	0.0001	0.9841
rs72934563	2	203995405	3.28E-16	0.0001	0.9843
rs72936838	2	203772984	3.28E-16	0.0001	0.9844
rs72936830	2	203769803	3.29E-16	0.0001	0.9845
rs140750546	2	203863736	3.30E-16	0.0001	0.9847
rs72936875	2	203791912	3.31E-16	0.0001	0.9848
rs142603618	2	203768786	3.32E-16	0.0001	0.9849
rs72932590	2	203884308	3.32E-16	0.0001	0.9850
rs147100405	2	203720774	3.33E-16	0.0001	0.9852
rs7560547	2	203757916	3.37E-16	0.0001	0.9853
rs145538381	2	203892767	3.37E-16	0.0001	0.9854
rs115130739	2	203725677	3.42E-16	0.0001	0.9855
rs72932727	2	203649501	3.45E-16	0.0001	0.9857
rs148513392	2	203744610	3.45E-16	0.0001	0.9858
rs7603972	2	203780515	3.46E-16	0.0001	0.9859
rs115827549	2	203725678	3.47E-16	0.0001	0.9860
rs72934550	2	203983940	3.49E-16	0.0001	0.9861
rs72934767	2	203766563	3.50E-16	0.0001	0.9863
rs72936881	2	203794262	3.52E-16	0.0001	0.9864
rs7591653	2	203780479	3.55E-16	0.0001	0.9865
rs114527590	2	203787405	3.56E-16	0.0001	0.9866
rs6732078	2	203656554	3.58E-16	0.0001	0.9867
rs72934545	2	203975958	3.59E-16	0.0001	0.9868
rs72936856	2	203775712	3.59E-16	0.0001	0.9870
rs6435168	2	203656846	3.60E-16	0.0001	0.9871
rs72936862	2	203786812	3.61E-16	0.0001	0.9872
rs72932588	2	203883193	3.62E-16	0.0001	0.9873

	rs72936866	2	203787120	3.64E-16	0.0001	0.9874
	rs72932753	2	203670122	3.65E-16	0.0001	0.9875
	rs72936872	2	203790889	3.66E-16	0.0001	0.9876
	rs72926771	2	203801226	3.70E-16	0.0001	0.9878
	rs114393235	2	203795987	3.72E-16	0.0001	0.9879
	rs72934751	2	203747522	3.73E-16	0.0001	0.9880
	rs72932765	2	203679183	3.75E-16	0.0001	0.9881
	rs72936879	2	203792628	3.78E-16	0.0001	0.9882
	rs72932767	2	203679306	3.80E-16	0.0001	0.9883
	rs72936870	2	203789679	3.86E-16	0.0001	0.9884
	rs72936873	2	203791333	3.87E-16	0.0001	0.9885
	rs140168762	2	203897946	3.90E-16	0.0001	0.9886
	rs72932722	2	203647598	3.90E-16	0.0001	0.9887
	rs72936882	2	203794439	3.91E-16	0.0001	0.9889
	rs115194657	2	203829284	3.92E-16	0.0001	0.9890
	rs72932716	2	203642244	3.92E-16	0.0001	0.9891
	rs72926769	2	203798318	3.94E-16	0.0001	0.9892
	rs75141346	2	203685119	3.96E-16	0.0001	0.9893
	rs72932770	2	203680954	3.96E-16	0.0001	0.9894
	rs143911965	2	203650998	3.97E-16	0.0001	0.9895
	rs72932707	2	203639395	3.99E-16	0.0001	0.9896
	rs72934753	2	203750272	4.01E-16	0.0001	0.9897
	rs148812085	2	203877233	4.06E-16	0.0001	0.9898
	rs72936847	2	203774748	4.06E-16	0.0001	0.9899
	rs72932772	2	203682304	4.08E-16	0.0001	0.9900
	rs4909945	11	10673739	2.28E-19	0.2489	0.2489
	rs7940646	11	10669228	2.53E-19	0.2250	0.4739
	rs4910165	11	10674044	3.29E-19	0.1734	0.6473
	rs2098839	11	10676987	4.62E-19	0.1239	0.7712
rs4909945	rs4442541	11	10669172	5.13E-19	0.1117	0.8829
	rs1863243	11	10677373	8.44E-19	0.0683	0.9512
	rs10840457	11	10675738	2.75E-18	0.0213	0.9725
	rs2052692	11	10667641	3.80E-18	0.0155	0.9880
	rs6484437	11	10667275	4.96E-18	0.0119	0.9999

CHR, chromosome; BP, position; CKD, chronic kidney disease; eGFR, estimated glomerular filtration rate; UACR, urinary albumin-to-creatinine ratio.

Supplementary Table 14. Colocalization analysis of index SNPs from cross-trait meta-analysis between migraine and chronic kidney disease.

Cross-trait GWAS model	SNP	N_{SNPs}	PP H_0	PP H_1	PP H_2	PP H_3	PP H_4
Migraine and CKD	**rs1047891**	2706	3.30E-04	2.01E-03	2.95E-03	0.017	**0.978**
Migraine and eGFR	rs1566225	1976	1.68E-26	2.29E-25	6.81E-02	**0.932**	0.000
	rs41272663	2206	7.00E-60	4.24E-59	1.25E-03	0.007	**0.992**
	**rs1047891**	2706	7.00E-60	4.26E-59	1.25E-03	0.007	**0.992**
	rs13099628	2768	6.03E-14	1.83E-13	2.47E-01	**0.752**	0.001
	rs6776700	1215	2.08E-08	3.16E-07	5.46E-03	0.082	**0.912**
	rs62576116	2633	8.46E-17	1.01E-06	8.42E-11	**1.000**	0.000
	rs9894634	2737	3.10E-05	4.27E-05	1.68E-02	0.022	**0.961**
Migraine and UACR	rs1971819	1565	7.64E-12	1.62E-09	3.58E-04	0.075	**0.925**
	**rs1047891**	2638	2.55E-14	1.55E-13	1.30E-03	0.007	**0.992**
	rs4909945	3462	5.71E-14	1.87E-04	3.04E-10	**0.994**	0.006

PP, posterior probability; CKD, chronic kidney disease; eGFR, estimated glomerular filtration rate; UACR, urinary albumin-to-creatinine ratio.

Supplementary Table 15. Multivariable Mendelian randomization analysis between migraine, blood pressure, and urinary albumin-to-creatinine ratio.

Outcome	Exposure	N_{SNPs}	Beta	Se	Beta (95%CI)	P
UACR	Migraine	298	0.008	0.007	0.008 (-0.006-0.022)	0.29
	SBP		0.010	0.001	0.010 (0.008-0.012)	1.08E-20
	Migraine	326	0.019	0.008	0.019 (0.003-0.034)	1.67E-02
	DBP		0.009	0.002	0.009 (0.005-0.012)	1.62E-06
	Migraine	525	0.009	0.007	0.009 (-0.006-0.023)	0.24
	SBP		0.011	0.002	0.011 (0.007-0.015)	5.17E-07
	DBP		-0.003	0.003	-0.003 (-0.009-0.004)	0.44

UACR, urinary albumin-to-creatinine ratio; SBP, systolic blood pressure; DBP, diastolic blood pressure.
